# Supplementary material for: An L-threonine transaldolase is required for L-threo-β-hydroxy-α-amino acid assembly during obafluorin biosynthesis
Source: Nat Commun. 2017 Jun 26;8:15935. doi: 10.1038/ncomms15935 (PMC5490192; doi:10.1038/ncomms15935)
Supplement: Supplementary Information [file ncomms15935-s1.pdf]

Type of file: pdf
Size of file: 0 KB
Title of file for HTML: Supplementary Information
Description: Supplementary Figures, Supplementary Tables, Supplementary Notes, Supplementary References.

Type of file: pdf
Size of file: 0 KB
Title of file for HTML: Peer Review File
Description:

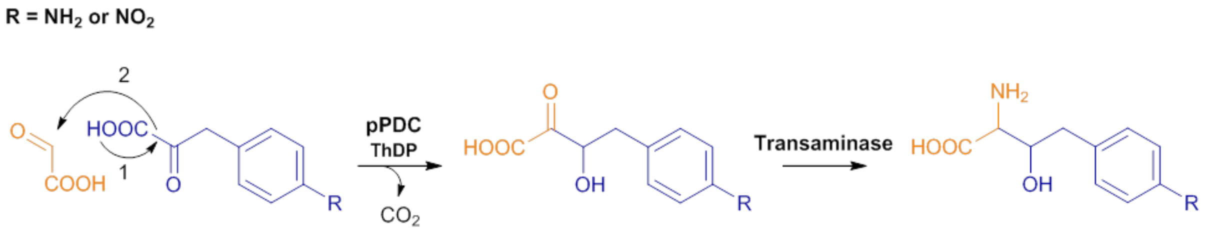

Supplementary Figure 1 | Biosynthesis of the β-hydroxy-α-amino acid moiety of obafluorin as proposed by Herbert and Knaggs[1].

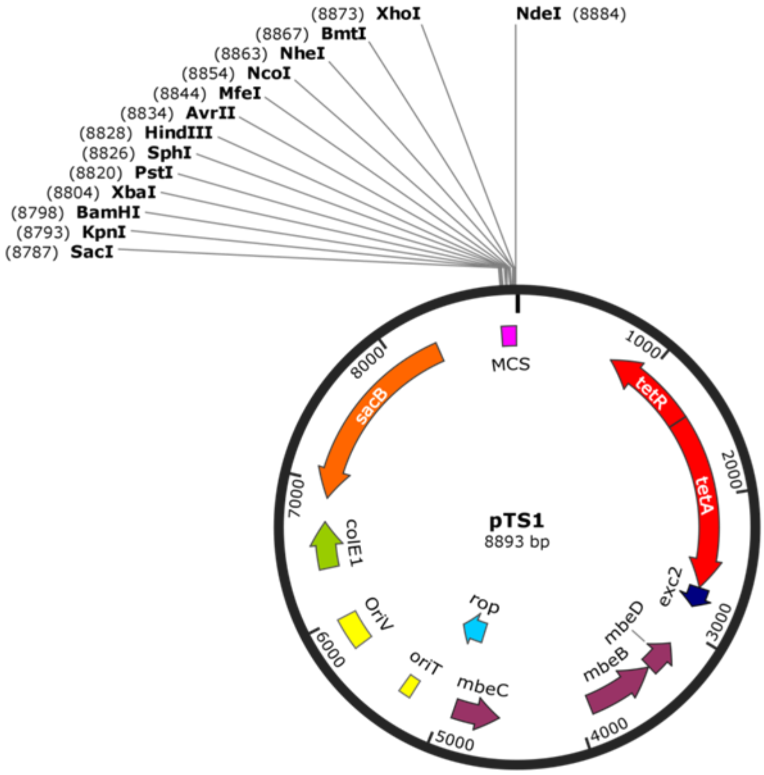

Supplementary Figure 2 | pTS1 suicide vector map. (Created with SnapGene®).

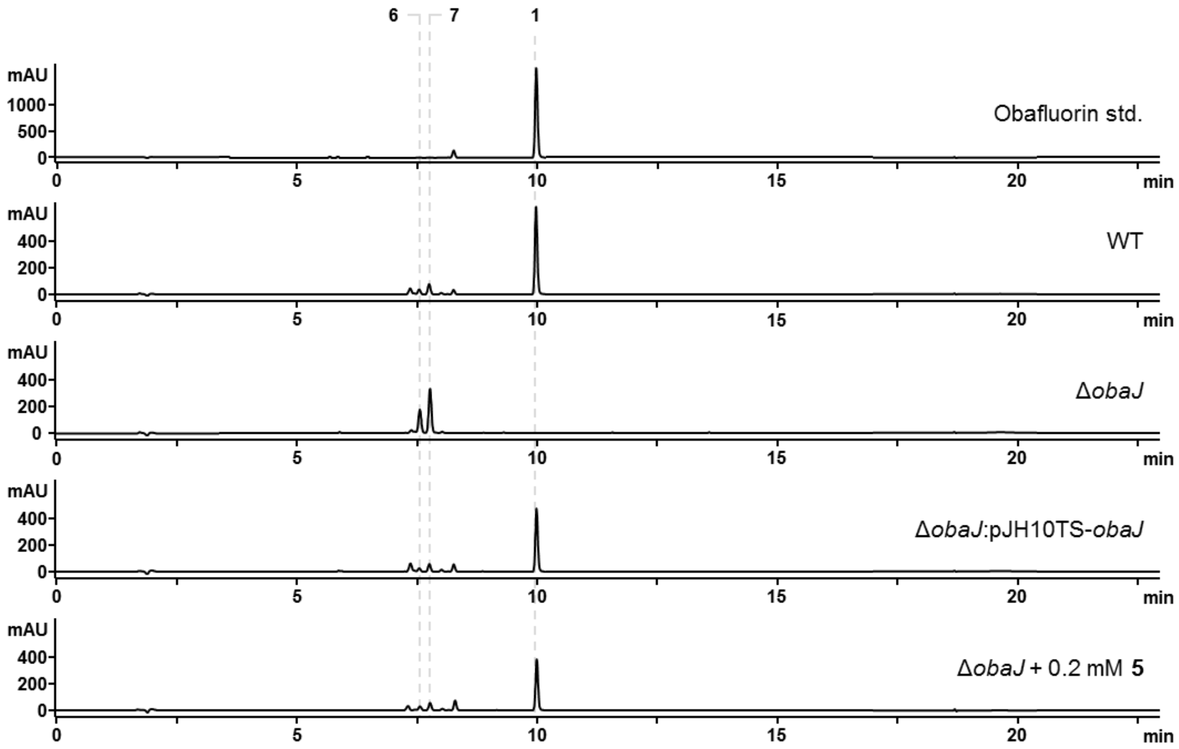

Supplementary Figure 3 | HPLC profiles for *obaJ* mutagenesis and complementation experiments at 270 nm. Numbered peaks refer to key products and shunt metabolites identified in Fig. 1.

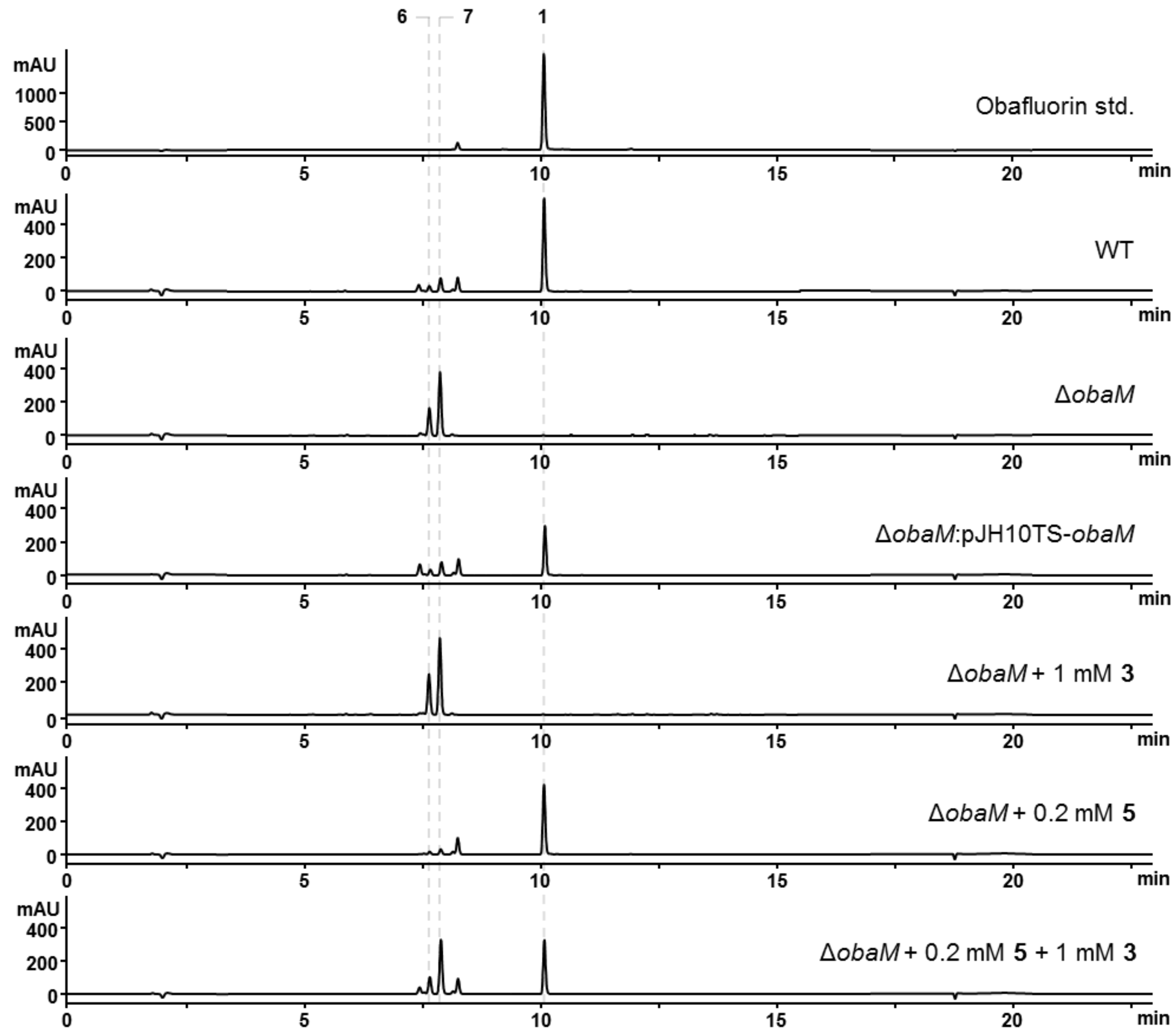

Supplementary Figure 4 | HPLC profiles for *obaM* mutagenesis and complementation experiments at 270 nm. Numbered peaks refer to key products and shunt metabolites identified in Fig. 1.

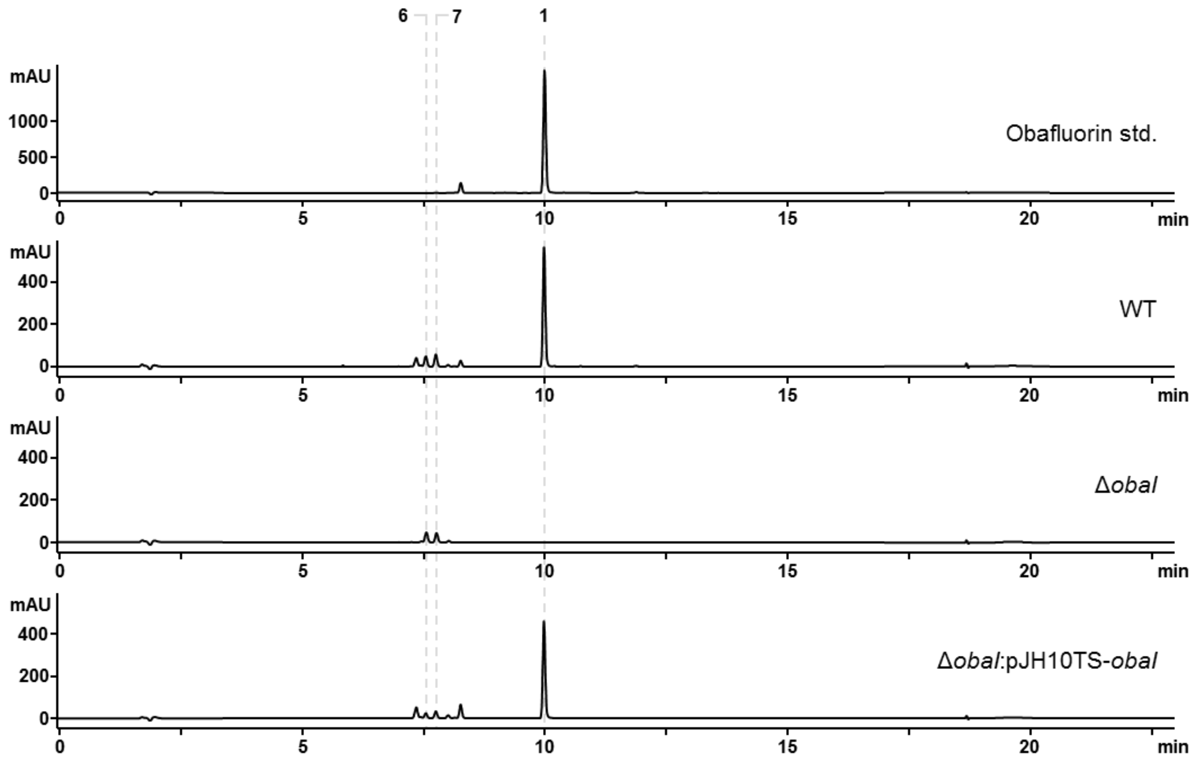

Supplementary Figure 5 | HPLC profiles for *obaC* mutagenesis and complementation experiments at 270 nm. Numbered peaks refer to key products and shunt metabolites identified in Fig. 1.

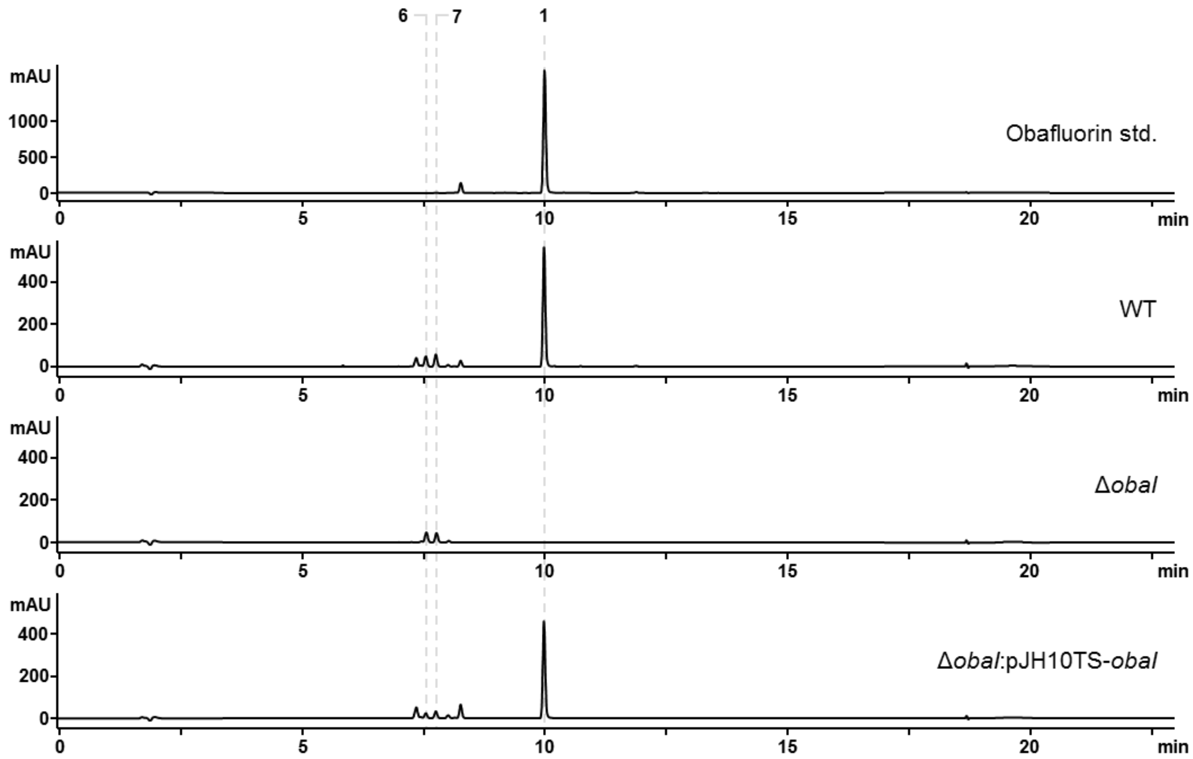

Supplementary Figure 6 | HPLC profiles for *obaI* mutagenesis and complementation experiments at 270 nm. Numbered peaks refer to key products and shunt metabolites identified in Fig. 1.

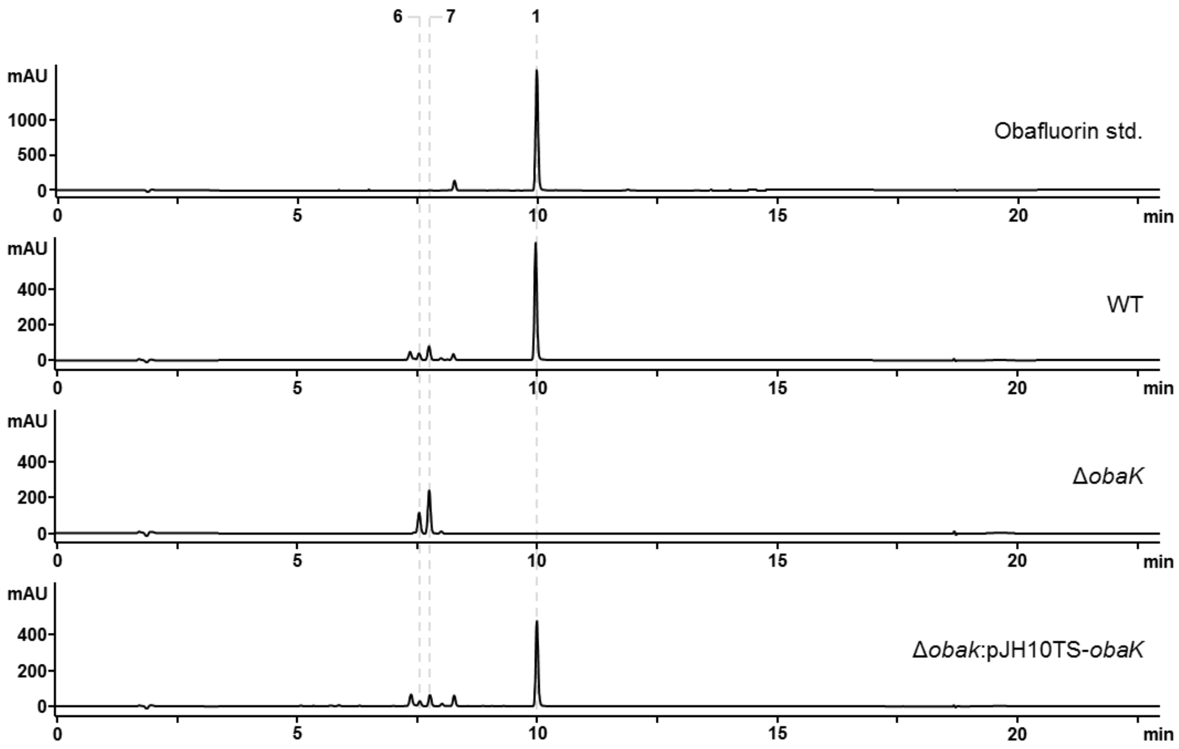

Supplementary Figure 7 | HPLC profiles for *obaK* mutagenesis and complementation experiments at 270 nm. Numbered peaks refer to key products and shunt metabolites identified in Fig. 1.

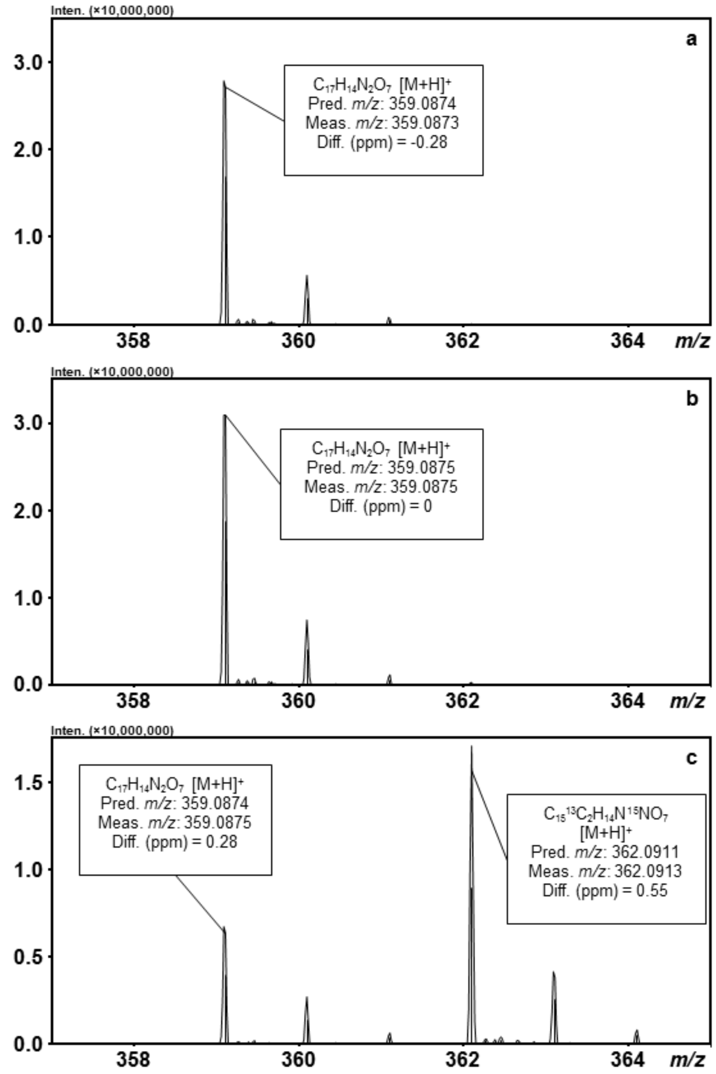

Supplementary Figure 8 | LCMS profiles for WT *P. fluorescens* production culture L-threonine feeding experiments. (**a**) ddH_2O negative control. (**b**) Fed 2 mM unlabelled L-threonine. (**c**) Fed 2 mM [U-$^{13}C_4$,$^{15}N_1$]L-threonine.

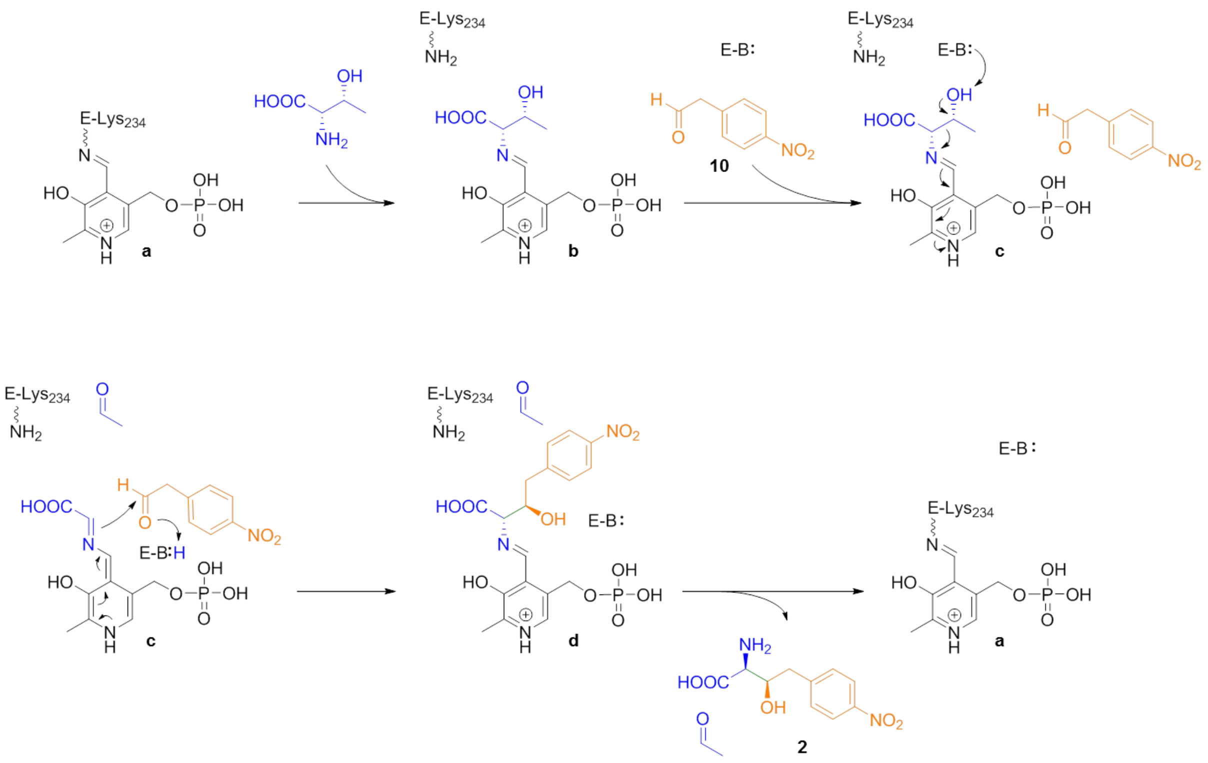

Supplementary Figure 9 | Proposed biochemical mechanism of ObaG L-TTA activity. Holo-ObaG (**a**) binds L-threonine to generate the external aldimine (**b**), and **10** to form a ternary complex. The bound L-threonine β-hydroxyl group is deprotonated by an enzyme base before nucleophilic attack on the *re* face in an aldol-type reaction to generate intermediate (**d**). **2** and acetaldehyde are then released to regenerate the holo-enzyme (**a**).

```
PaGlyA1     1 MFSKHDQIRGYDDELLAAMDAEEARQEDHLELIASENYTSKRVMQAQGSGLTNKYAEGYP
PfGlyA      1 MFSRDLTIAKYDADLFAAMEQEAVRQEEHIELIASENYTSPAVMEAQGSVLTNKYAEGYP
EcGlyA      1 MLKREMNIADYDAELWQAMEQEKVRQEEHIELIASENYTSPRVMQAQGSQLTNKYAEGYP
BsGlyA      1 ----MKYLPQQDPQVFAAIEQERKRQHAKIELIASENFVSRAVMEAQGSVLTNKYAEGYP
ObaG        1 MSNVKQQTAQIVDWLSSTLGKDHQYREDSLSLTANENYPSALVRLTSGSTAGAFYHCSFP

PaGlyA1    61 -----GKRYYGGCEHVDKVERLAIDRARQLFGADYANVQPHSGSSANAAVYLALLNAGDT
PfGlyA     61 -----GKRYYGGCEYVDVVEQLAIDRAKELFGADYANVQPHAGSQANSAVYLALLQGGDT
EcGlyA     61 -----GKRYFGGCEYVDIVEQLAIDRAKELFGADYANVQPHSGSQANFAVYTALLEPGDT
BsGlyA     57 -----GRRYYGGCEYVDIVEELARERAKQLFGAEHANVQPHSGAQANMAVYFTVLEHGDT
ObaG       61 FEVPAGEWHFPEPGHMNAIADQVRDLGKTLIGAQAFDWRPNGGSTAEQALMLAACKPGEG

PaGlyA1   116 ILGMSLAHGGHLTHGAKVSSSGKLYNAVQYGLDTATGLIDYDEVERLAVEH-KPKMIVAG
PfGlyA    116 ILGMSLAHGGHLTHGASVSSSGKLYNAVQYGID-ANGLIDYDEVERLAVEH-KPKMIVAG
EcGlyA    116 VLGMNLAHGGHLTHGSPVNFSGKLYNIVPYGID-ATGHIDYADLEKQAKEH-KPKMIIGG
BsGlyA    112 VLGMNLSHGGHLTHGSPVNFSGVQYNFVAYGVDPETHVIDYDDVREKARLH-RPKLIVAA
ObaG      121 FVHFAHRDGGHFALESLAQKMG--IEIFHLPVNPTSLLIDVAKLDEMVRRNPHIRIVILD

                                                                    ▼
PaGlyA1   175 FSAYSKTLDFPRFRAIADKVGALLFVDMAHVAGLVAAGLYPNPIPF-ADVVTTTTHKTLR
PfGlyA    174 FSAYSQIIDFPRFRAIADKVGAYLFVDMAHVAGLVAAGVYPNPVPY-ADVVTTTTHKTLR
EcGlyA    174 FSAYSGVVDWAKMREIADSIGAYLFVDMAHVAGLVAAGVYPNPVPH-AHVVTTTTHKTLA
BsGlyA    171 ASAYPRIIDFAKFREIADEVGAYLMVDMAHIAGLVAAGLHPNPVPY-AHFVTTTTHKTLR
ObaG      179 QSFKLRWQPLAEIRSVLPDS-CTLTYDMSHDGGLIMGGVFDSPLSCGADIVHGNTHKTIP

PaGlyA1   234 GPRGGLILAR-ANEEIEKKLNSAVFPGAQGGPLMHVIAAKAVCFKEALEPGFKDYQAQVI
PfGlyA    233 GPRGGLILAR-ANAEIEKKLNSAVFPGAQGGPLEHVIAAKAICFKEALQPEFKTYQQQVV
EcGlyA    233 GPRGGLILAKGGSEELYKKLNSAVFPGGQGGPLMHVIAGKAVALKEAMEPEFKTYQQQVA
BsGlyA    230 GPRGGMILCQ---EQFAKQIDKAIFPGIQGGPLMHVIAAKAVAFGEALQDDFKAYAKRVV
ObaG      238 GPQKGYIGFKSAQHPLLVDTSLWVCPHLQSNCHAEQLPPMWVAFKEMELFG-RDYAAQIV

PaGlyA1   293 RNAKAMAEVFIGRGYDVVSGG---TDNHLMLISLVRQGLTGKEADAALGRVGITVNKNAV
PfGlyA    292 KNAQTMASVFIERGFDVVSGG---TENHLFLLSLIKQDISGKDADAALGKAFITVNKNSV
EcGlyA    293 KNAKAMVEVFLERGYKVVSGG---TDNHLFLVDLVDKNLTGKEADAALGRANITVNKNSV
BsGlyA    287 DNAKRLASALQNEGFTLVSGG---TDNHLLLVDLRPQQLTGKTAEKVLDEVGITVNKNTI
ObaG      297 SNAKTLARHLHELGLDVTGESFGFTQTHQVHFAVGDLQKALDLCVNSLHAGGIRSTNIEI

PaGlyA1   350 PNDPQSPFVTSGIRIGTPAITTRGLQEAQSRELAGWICDILDHLGDADVEAKVATQVAGL
PfGlyA    349 PNDPRSPFVTSGLRFGTPAVTTRGFKEAECKELAGWICDILADLNNEAVIDAVREKVKAI
EcGlyA    350 PNDPKSPFVTSGIRVGTPAITRRGFKEAEAKELAGWMCDVLDSINDEAVIERIKGKVLDI
BsGlyA    344 PYDPESPGVTSGIRIGTAAVTTRGFGLEEMDEIAAIIGLVLKNVGSEQALEEARQRVAAL
ObaG      357 PGKPG----VHGIRLGVQAMTRRGMKEKDFEVVARFIADLYFKKTEPAKVAQQIKEFLQA

PaGlyA1   410 CADFPVYR--------------------
PfGlyA    409 CKKLPVYGA-------------------
EcGlyA    410 CARYPVYA--------------------
BsGlyA    404 TD--------------------------
```

Supplementary Figure 10 | Sequence alignment of authentic and putative SHMT amino acid sequences. ObaG from the obafluorin gene cluster is aligned (ClustalX2) with predicted SHMTs from *Pseudomonas fluorescens* SBW25 (PfGlyA, WP_015885982.1) and *P. aeruginosa* PA01 (PaGlyA1, NP_254102.1) whole-genome sequences, and validated SHMTs with solved structures from *Escherichia coli* (EcGlyA, 1EQB_B) and *Bacillus stearothermophilus* (BsGlyA, 2VMV_A). ▼ indicates the conserved lysine residue required for internal aldimine formation with PLP.

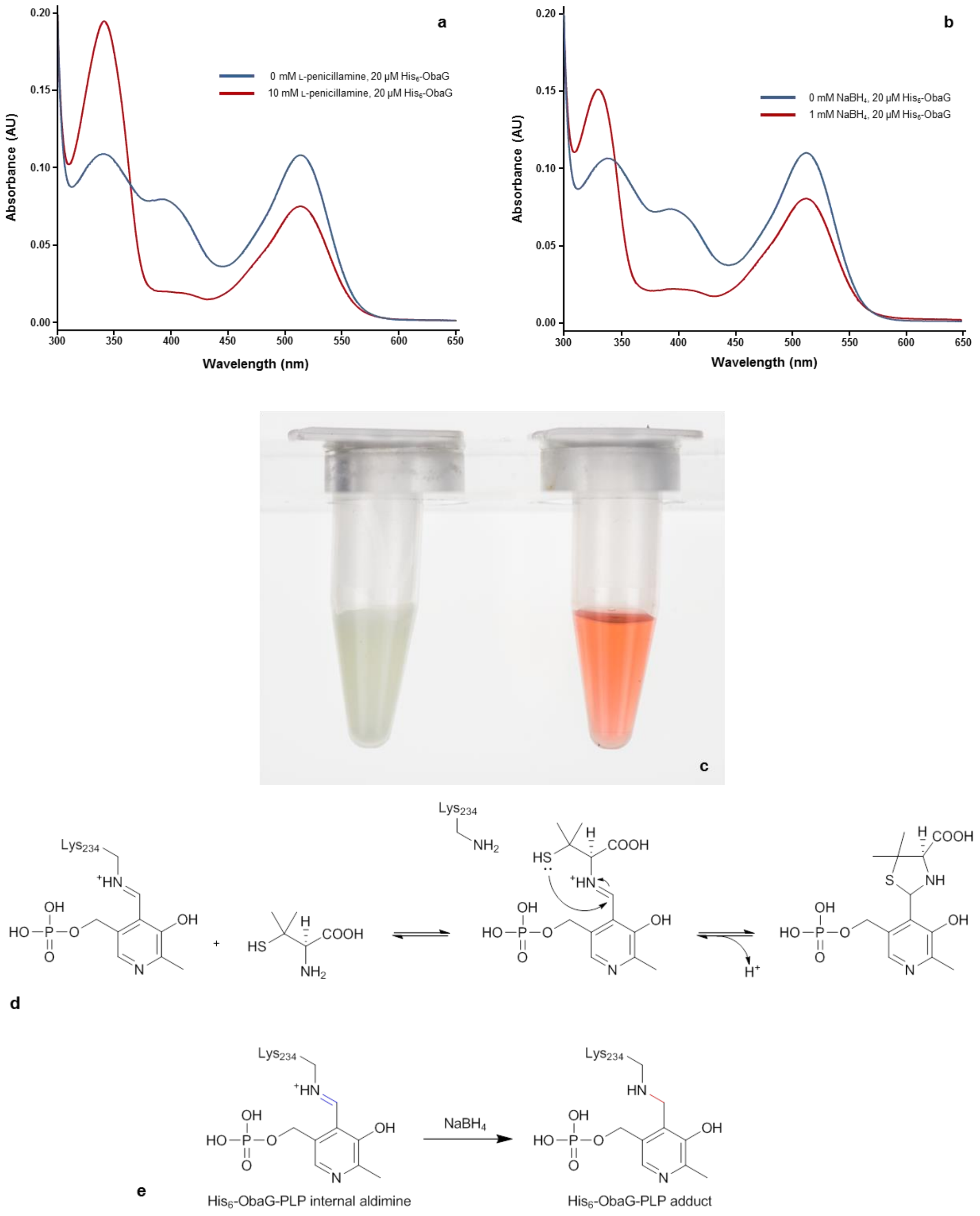

Supplementary Figure 11 | Experimental confirmation that ObaG is a PLP-dependent enzyme. (**a**) UV/Vis spectrum of 20 µM His$_6$-ObaG (blue) showing peaks at 340 nm and 390 nm, representing the enolimine and ketoenamine forms of the PLP cofactor respectively. Addition of 10 mM L-penicillamine (red) led to formation of a thiazolidine adduct (340 nm peak) and concomitant loss of the 390 nm peak following 30 min. (**b**) UV/Vis spectrum of 20 µM His$_6$-ObaG (blue). Addition of 1 mM $NaBH_4$ (red) led to formation of a covalent His$_6$-ObaG-PLP adduct (330 nm peak) and concomitant loss of the 390 nm ketoenamine peak following 15 min on ice. (**c**) The enzyme is salmon pink/pink in solution (right), but following boiling (left), the pink colour is lost and instead a feint green/yellow can be seen, indicating release of the PLP cofactor. (**d**) L-penicillamine reacts with PLP via an external aldimine intermediate to form a thiazolidine adduct. (**e**) Reduction of the internal aldimine formed between the enzyme catalytic lysine residue and PLP to form a secondary amine adduct.

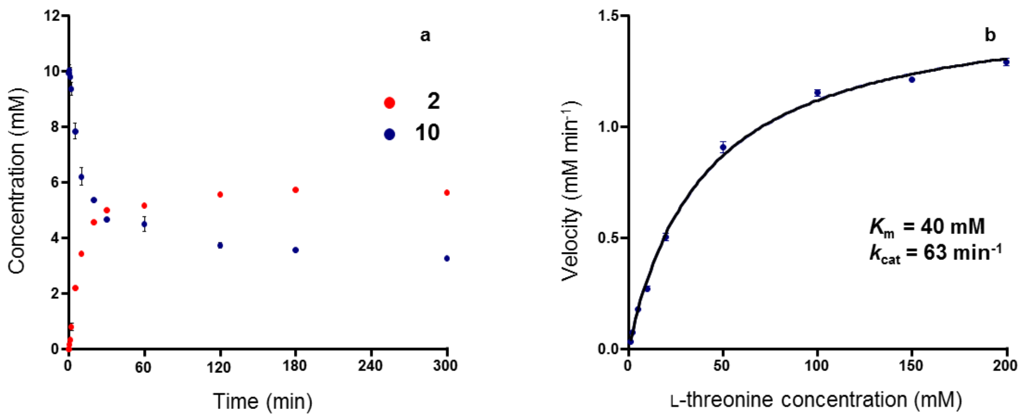

Supplementary Figure 12 | Further biochemical characterisation and kinetic analysis of His$_6$-ObaG. (**a**) Time course of the discontinuous L-TTA activity assay monitoring depletion of substrate **10** and generation of product **2**. (**b**) Single-substrate kinetic analysis of the L-TTA reaction with 10 mM **10** and variable L-threonine (1-200 mM). All data are mean values of three (**a**) or five (**b**) independent experiments and error bars represent the s.e.m. **2** and **10** are identified in Fig. 1.

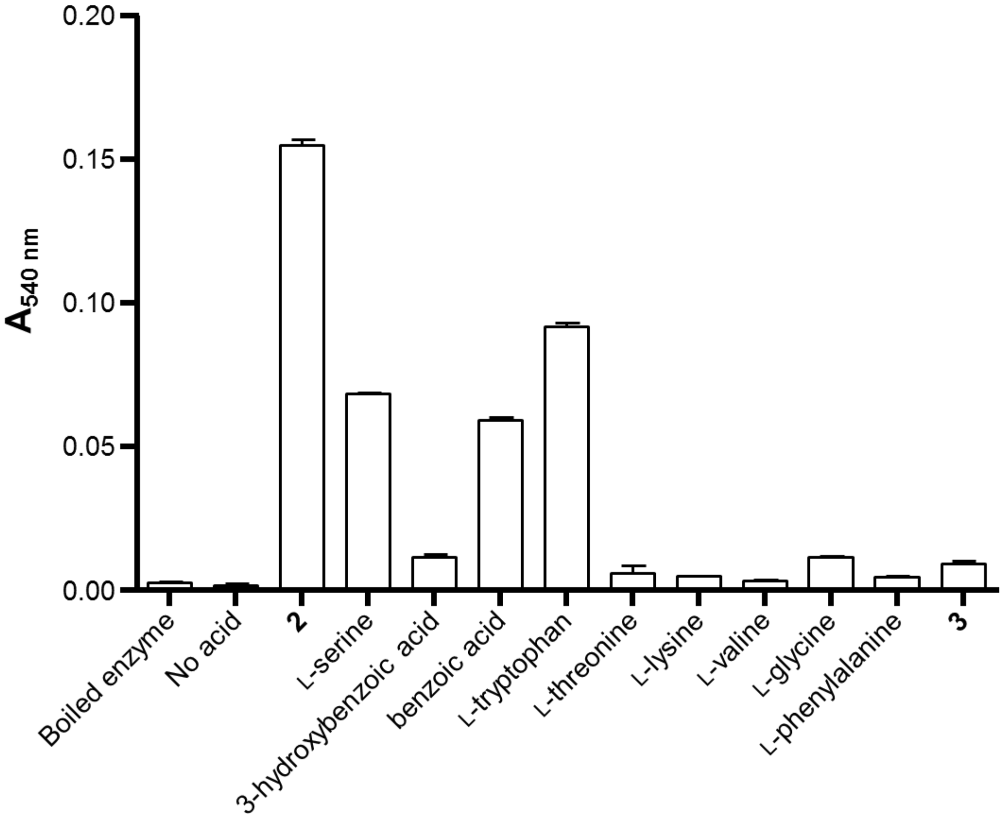

Supplementary Figure 13 | Hydroxylamine-trapping assay results. His_6-ObaI adenylation domain specificity was assayed using a range of amino and carboxylic acid substrates. All data are mean values of three independent experiments and error bars represent the s.e.m. Numbered substrates are identified in Fig. 1.

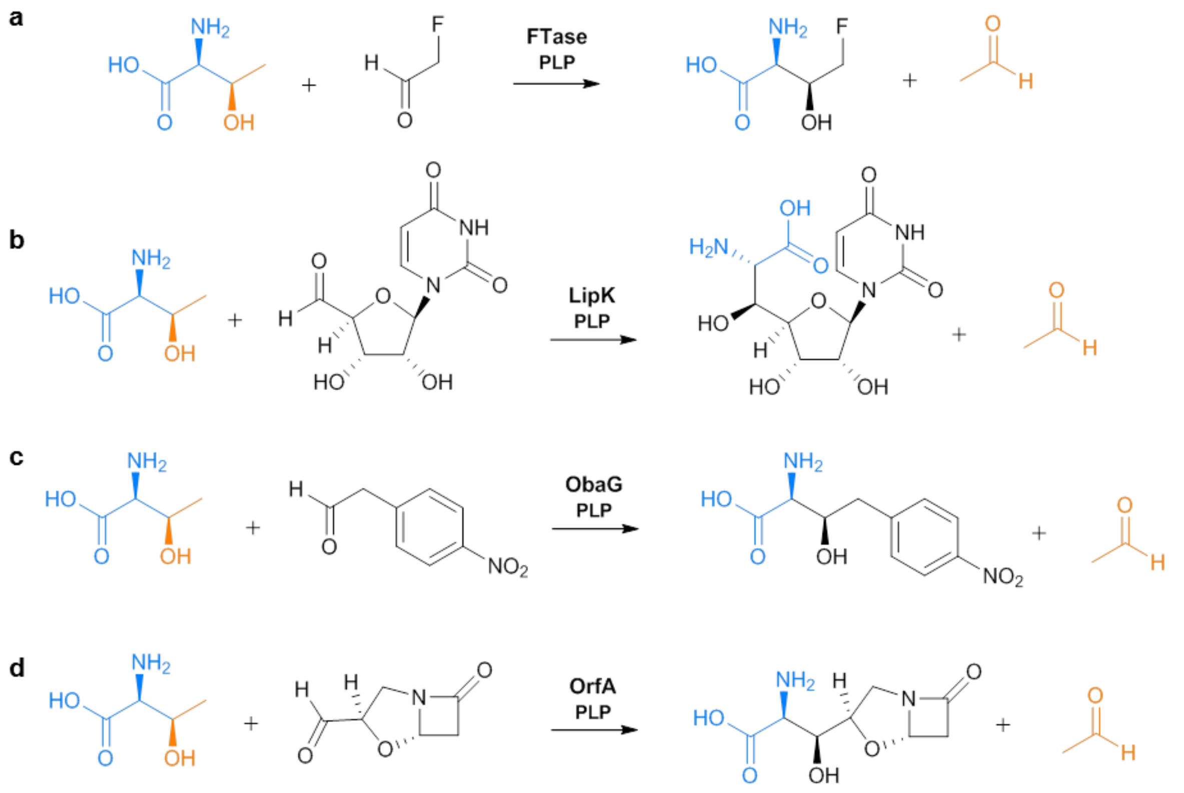

Supplementary Figure 14 | L-TTA reactions characterised or described in the literature and this work. (**a**) FTase, (**b**) LipK, (**c**) ObaG, and (**d**) OrfA.

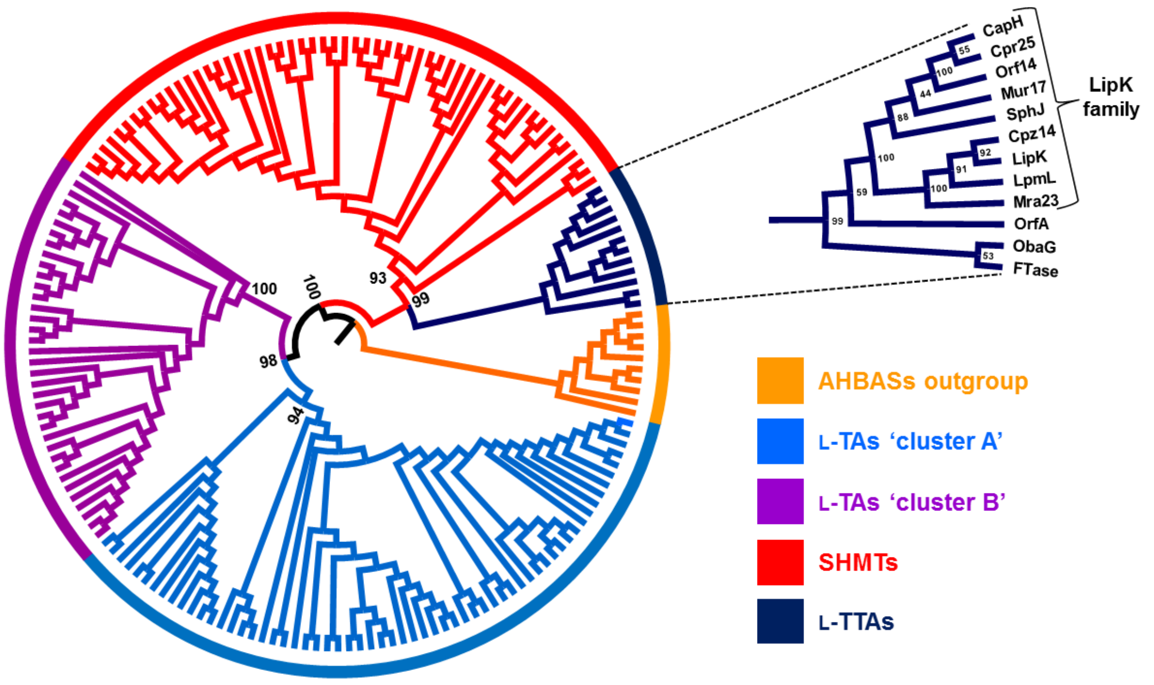

Supplementary Figure 15 | Maximum likelihood tree of L-TAs, SHMTs and L-TTAs version 2. A set of AHBAS amino acid sequences serve as the outgroup. RAxML likelihood values at the root nodes for SHMT and L-TA cluster clades are annotated. The clade comprising the L-TTA ObaG characterised in this work has been expanded and annotated with RAxML likelihood values and the identity of amino acid sequences represented. Initial 'conservative' trimming performed following alignment in ClustalX2.

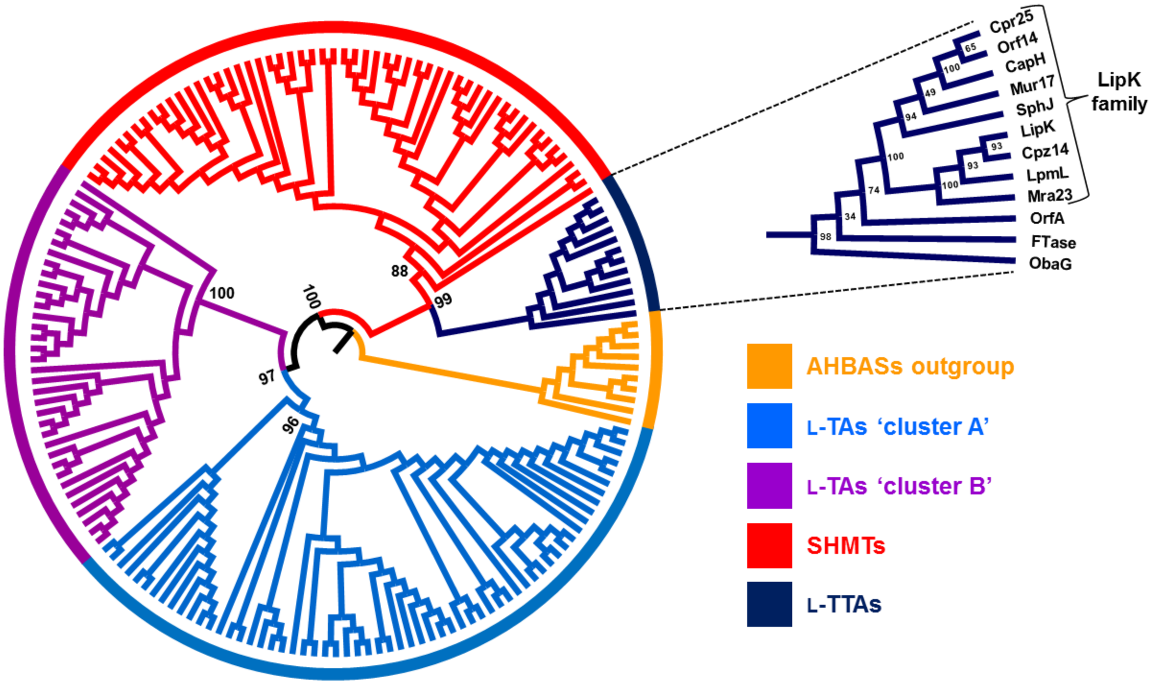

Supplementary Figure 16 | Maximum likelihood tree of L-TAs, SHMTs and L-TTAs version 3. A set of AHBAS amino acid sequences serve as the outgroup. RAxML likelihood values at the root nodes for SHMT and L-TA cluster clades are annotated. The clade comprising the L-TTA ObaG characterised in this work has been expanded and annotated with RAxML likelihood values and the identity of amino acid sequences represented. Initial trimming performed following alignment in MUSCLE.

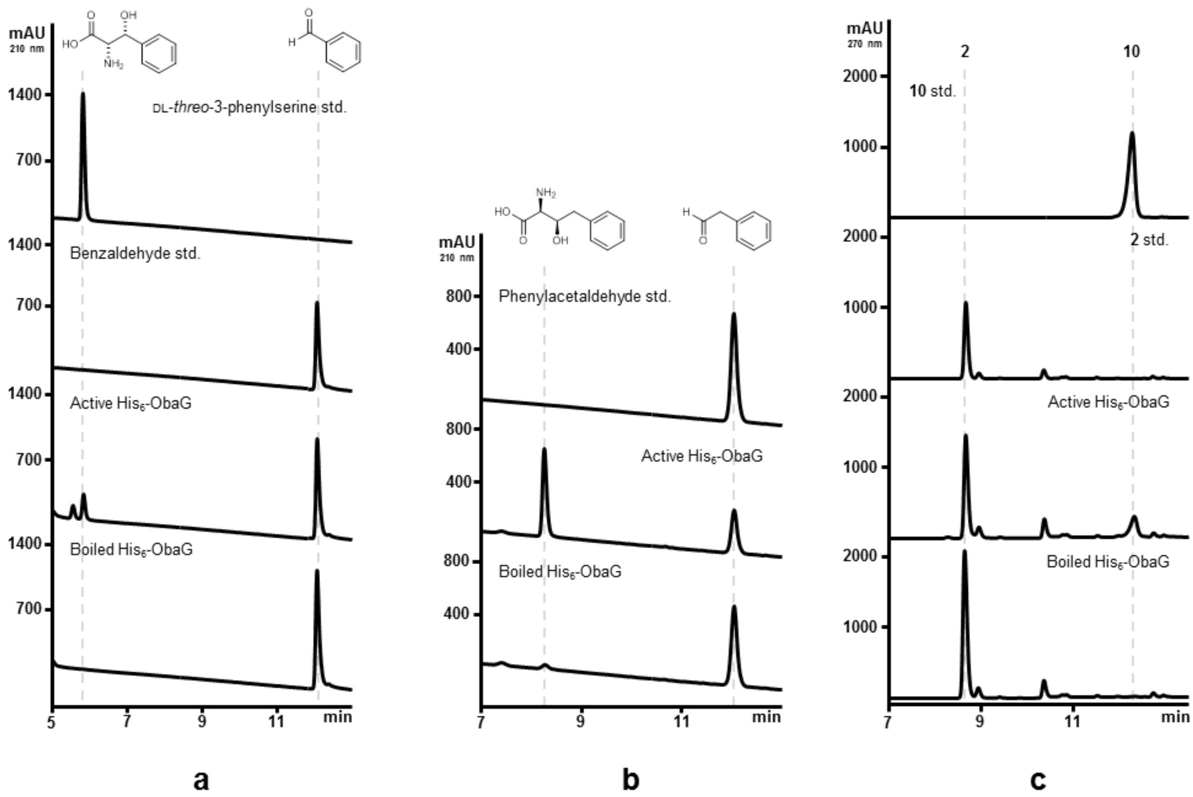

Supplementary Figure 17 | HPLC profiles for His$_6$-ObaG L-TTA assays with L-threonine and alternative aldehyde substrates, and the reverse L-TTA reaction with 2 and acetaldehyde. (**a**) His$_6$-ObaG L-TTA activity assay with benzaldehyde and L-threonine as substrates. (**b**) His$_6$-ObaG L-TTA activity assay with phenylacetaldehyde and L-threonine as substrates. (**c**) His$_6$-ObaG reverse L-TTA activity assay with **2** and acetaldehyde as substrates. Structures refer to the substrate and product of each reaction, and numbered peaks refer to compounds identified in Fig. 1.

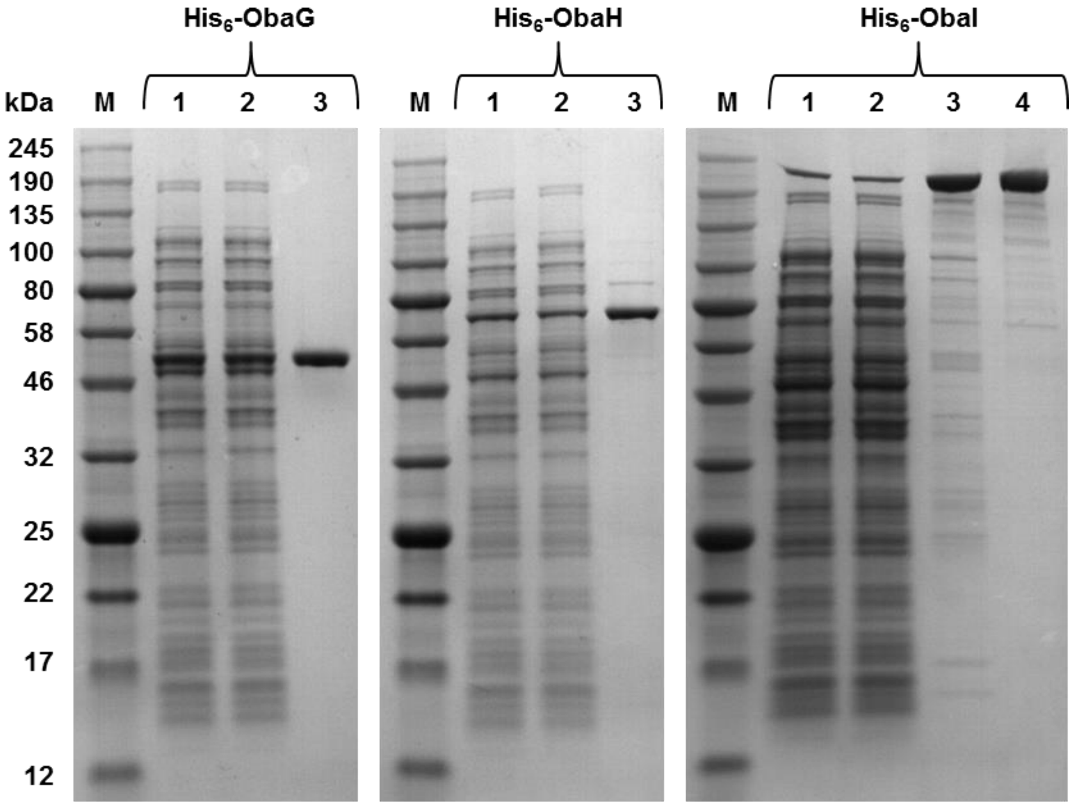

Supplementary Figure 18 | 12 % SDS PAGE gel of His$_6$-ObaG to I purification steps. 1 = Cell lysate, 2 = Chitin wash eluent, 3 = Ni-affinity purified sample, 4 = Size exclusion purified sample, and M = Colour Prestained Protein Standard, Broad Range (NEB). The expected molecular weights for His$_6$-ObaG, H and I are 50.8, 64.2 and 211.5 kDa respectively.

```
LipK     1 ---------MTVGAGGKTSADADPLMLVRAIADADRRAAHALNLVPSENRISPLASLPLA
FTase    1 MPSSVNRTSRTEPAGHHREFPLSLAAIDELVAEEEAEDARVLHLTANETVLSPRARAVLA
ObaG     1 ------------MSNVKQQTAQIVDWLSSTLGKDHQYREDSLSLTANENYPSALVRLTSG

LipK    52 SDFYNRYFFN-------TDGDPLFWEFRGGEDIAHIEALGAAALRRMASARYCNVRPISG
FTase   61 SPLTSRYLLEHLDMRGPSPARLGNLLLRGLDRIGTIEESATEVCRRLFGARYAEFRCLSG
ObaG    49 STAGAFYHCS-----FPFEVPAGEWHFPEPGHMNAIADQVRDLGKTLIGAQAFDWRPNGG

LipK   105 MSAMILTVAALSPPGSTVVSVDQNSGGHYATPALLGRLGRRSRLLNCK-DGE-VDESELA
FTase  121 LHAMQTTFAALSRPGDTVMRVATKDGGHFLTELICRSFGRRSCTYVFD-DTMTIDLERTR
ObaG   104 STAEQALMLAACKPGEGFVHFAHRDGGHFALESLAQKMGIEIFHLPVNPTSLLIDVAKLD

LipK   163 EVLAPG-DVALVYVDVQNCVRVPDFRRMSDVIREVSPGTRLYVDASHYLGLVLGGLLANP
FTase  180 EVVEKE-RPSLLFVDAMNYLFPFPIAELKAIAGDVP----LVFDASHTLGLIAGGRFQDP
ObaG   164 EMVRRNPHIRIVILDQSFKLRWQPLAEIRSVLPDSCT---LTYDMSHDGGLIMGGVFDSP

                        ▼
LipK   222 LDCGADAFGGSTHKSFPGPHKGVIFTN--AEDVDESLR-SAQFDLVSSHHFAETLALSLA
FTase  235 LREGADLLQANTHKTFFGPQKGIILGN--DRSLMEELGYTLSTGMVSSQHTASTVALLIA
ObaG   221 LSCGADIVHGNTHKTIPGPQKGYIGFKSAQHPLLVDTSLWVCPHLQSNCHAEQLPPMWVA

LipK   279 ALEVEDRMGDYARATNDNARRLAGALADAGFRVYGDSATGYTDTHQVWVELDGVAAAYAL
FTase  293 LHEMWYDGREYAAQVIDNARRLAGALRDRGVPVVAE-ERGFTANHMFFVDTRPLGSGPAV
ObaG   281 FKEMELFGRDYAAQIVSNAKTLARHLHELGLDVTGE-SFGFTQTHQVHFAVGDLQKALDL

LipK   339 SNR-LAEGGIRVNLQSSMPGMSGVHLRLGSNEVTFEGAGPQAIEELAGALVTARER----
FTase  352 IQR-LVRAGVSANRAVAFNHLD--TIRFGVQEITRRGYDHDDLDEAADLVAAVLLERQEP
ObaG   340 CVNSLHAGGIRSTNIEIPGKPGVHGIRLGVQAMTRRGMKEKDFEVVARFIADLYFKKTEP

LipK   394 -ALGPRTVHEIRGRFGAPFYTDPEKLKVEAGL----------------------------
FTase  409 ERIRPRVAELVGRRRTVRYTGDPASAAGPPARERYAPPTAPAGHPARPRWIGVRLTPLPE
ObaG   400 AKVAQQIKEFLQAFPLAPLAYSFDNYLDEELLAAVYQGAQR-------------------

LipK       ------------------------------------------------------------
FTase  469 PVTEAECAGAQRLGRLAGAFPHQIDSSGNVSFTSTDGRLFVTGSGTYIKDLAPGDFVELT
ObaG       ------------------------------------------------------------

LipK       ------------------------------------------------------------
FTase  529 GAEGWTLHCRGDGPPSAEAYLHHLLRERVGARYVVHNHCIPGRALETSGALVIPPKEYGS
ObaG       ------------------------------------------------------------

LipK       ----------------------------------------------
FTase  589 VALAEAVADACQDSQVMYVRRHGLVFWAHSYDECLALIEDVRRITG
ObaG       ----------------------------------------------
```

Supplementary Figure 19 | Sequence alignment of biochemically characterised L-TTAs. ObaG from the obafluorin gene cluster is aligned (ClustalX2) with 4-fluorothreonine transaldolase from *Streptomyces cattleya* (WP_014151017.1)[7] and LipK from *S.* sp. SANK 60405 (BAJ05887.1)[8]. FTase comprises an *N*-terminal SHMT-like domain and a *C*-terminal phosphate binding domain similar to those of bacterial aldolases and empimerases. ObaG shares 29 % identity (88 % query cover) to the *N*-terminal domain of FTase, and 33 % identity (67 % query cover) with LipK. ▼ indicates the conserved lysine residue required for internal aldimine formation with PLP.

Protein/Gene	Size (Da/bp)	Proposed function	Conserved domains (Based on Pfam and NCBI conserved domains)	GenBank accession ID of protein homologue (query cover/identity %)
Orf1/*orf1*	54742.50/1509	Transcriptional regulator	ACT domain; PAS domain; Sigma-54 interaction domain; PAS domain; HTH domain	WP_065949357.1 (100/100)
Orf2/*orf2*	13798.36/384	Glycine cleavage system H-protein	Biotinyl lipoyl domain	WP_065949356.1 (100/100)
Orf3/*orf3*	102206.59/2838	Glycine dehydrogenase (Glycine cleavage system P-protein)	PLP-dependent aspartate aminotransferase (fold-type I) domain	WP_065949355.1 (100/99)
Orf4/*orf4*	48961.74/1377	L-serine ammonia lyase	L-serine dehydratase alpha and beta chains	WP_065949354.1 (100/99)
Orf5/*orf5*	40424.23/1125	Aminomethyltransferase (Glycine cleavage system T-protein)	Folate-binding domain; glycine cleavage T-protein *C*-terminal barrel domain	WP_065949353.1 (100/99)
Orf6/*orf6*	7678.65/213	Cold-shock protein	DNA-binding domain	WP_065949352.1 (100/100)
Orf7/*orf7*	18377.98/489	Transmembrane protein	RDD domain	WP_065949351.1 (93/100)*
Orf8/*orf8*	38252.36/1059	Quinolinate synthetase	Quinolinate synthetase A protein	WP_065949350.1 (100/100)
ObaA/*obaA*	26341.56/714	Transcriptional regulator	Autoinducer-binding domain ; DNA-binding HTH domain	WP_065949349.1 (100/99)
ObaB/*obaB*	18690.35/510	*N*-acylhomoserine lactone synthase	*N*-acyltransferase domain	WP_065949348.1 (100/100)
ObaC/*obaC*	36033.87/951	*N*-oxygenase	Ferritin-like domain	WP_065936855.1 (100/99)
ObaD/*obaD*	22678.28/600	4-amino-4-deoxychorismate synthase component II	GATase-1 domain	CRM14850.1 (100/100)
ObaE/*obaE*	54701.47/1461	4-amino-4-deoxychorismate synthase component I	Anthranilate synthase component I, *N*-terminal region; chorismate binding domain	CRM14833.1 (100/100)
ObaF/*obaF*	46268.45 /1263	Bifunctional 4-aminochorismate mutase/4-aminoprephenate dehydrogenase	Rossman-fold NADB domain; chorismate mutase type II	WP_065949346.1 (100/100)
ObaG/*obaG*	48592.95/1323	L-Threonine transaldolase	PLP-dependent aspartate aminotransferase (fold-type I) domain	WP_065949345.1 (100/100)
ObaH/*obaH*	62078.40/1704	4-nitrophenylpyruvate decarboxylase	ThDP-dependent enzyme pyrimidine-binding domain; ThDP-dependent enzyme central domain (2-fold Rossman fold); ThDP-binding domain	WP_065949344.1 (100/100)
ObaI/*obaI*	209339.56/5733	Dimodular nonribosomal peptide synthase	Condensation domain (C_2); AMP-binding domain (A_2); PP-binding domain (PCP_2); Thioesterase domain (TE_2); MbtH-like domain; AMP-binding domain (A_1)	WP_065949343.1 (100/99)
ObaJ/*obaJ*	23689.61/645	Isochorismatase	Cysteine-hydrolase domain	WP_065949342.1 (100/100)
ObaK/*obaK*	10073.63/267	Aryl carrier protein	PP-binding domain (ArCP1)	WP_065949341.1 (100/100)
ObaL/*obaL*	26832.77/771	2,3-dihydro-2,3-dihydroxybenzoate dehydrogenase	Rossman-fold NADB domain	WP_065949340.1 (100/99)
ObaM/*obaM*	44615.02/1197	DAHP synthase	DAHP synthetase class II domain	WP_065949339.1 (89/100)*
ObaN/*obaN*	52693.61/1419	Isochorismate synthase	Chorismate-binding domain	WP_065949338.1 (100/99)
Orf9/*orf9*	72153.72/1914	Threonine tRNA ligase	TGS domain; tRNA SAD domain; HMG-box DNA-binding domain; threonyl-tRNA synthetase class II core catalytic domain; threonyl anticodon-binding domain	WP_065949337.1 (100/100)
Orf10/*orf10*	17007.34/471	Polyketide cyclase	PYR1-like SRPBCC domain	WP_065949336.1 (100/99)
Orf11/*orf11*	NA/73	tRNA-lysine (TTT)	NA	NA
Orf12/*orf12*	24252.64/648	7-carboxy-7-deazaguanine synthase	4e-4s single cluster domain; radical SAM superfamily domain	WP_065949335.1 (100/99)
Orf13/*orf13*	21974.62/630	Tol/Pal system protein	*N*-terminal trimerisation domain; BamD superfamily domain	WP_065949334.1 (100/100)
Orf14/*orf14*	45916.31/1260	Tol/Pal system translocation protein	TolB amino-terminal domain; WD40-like beta propeller repeat (×3)	WP_065887603.1 (100/100)
Orf15/*orf15*	38686.91/1074	TolA-like protein	TonB family *C*-terminal domain	WP_034115767.1 (100/100)

Supplementary Table 1 | Identified protein coding sequences (PCSs) for the obafluorin biosynthetic gene cluster. * indicates where we have identified a different start codon for a particular PCS.

Plasmid/Strain	Genotype/description	Reference
**Plasmid**		
pME3087	Suicide vector; *ColE1* replicon, *IncP-1* , *Mob* ; Tc^R	2
pTS1	pME3087 modified with *sacB* counter-selection and an expanded multiple cloning site	This work
pJH10	Vector for complementation studies by *in trans* expression in *P. fluorescens* ATCC 39502 Δ strains pOLE1 with *IncC1* deleted, EcoRI-SacI polycloning site, Tc^R from pDM1.2	5
pJH10TS	pJH10 modified with an expanded cloning site	This work
pET28a(+)	Expression vector; $Kan^{r,}$ the transcription of the cloned gene is driven by the T7 RNA polymerase and controlled by the LacI repressor, *ColE1* replicon	Novagen
**Strain**		
E. coli DH5α	Host for general cloning; F^- *endA1 glnV44 thi-1 recA1 relA1 gyrA96 deoR nupG* φ80*dlacΔ(lacZ)M15* Δ(*lacIZYA-argF*)U169 *hsdR* 17(r_K^- m_K^+) λ–	Lab stock
E. coli S17-1 λ(pir)	Donor strain for conjugation between *E. coli* and *P. fluorescens* ATCC 39502; *recA thi pro hsd* (R^- M^+)RP4: 2- Tc::Mu- Km::Tn7 λpir SM^R Tp^R	Lab stock
E. coli NiCo21(DE3) pLysS	Expression strain; *can::CBD fhuA2 [lon] ompT gal (λ DE3) [dcm] arnA::CBD slyD::CBD glmS6Ala ΔhsdS λ DE3 = λ sBamHIo ΔEcoRI-B int::(lacI::PlacUV5::T7 gene1) i21 Δnin5* (pLysS; Cm^R, p15A replicon, T7 lysozyme coding seq.)	New England Biolabs
P. fluorescens ATCC 39502	WT obafluorin producing strain	This work
P. fluorescens Δ*obaC*	*P. fluorescens* ATCC 39502 with an in-frame truncation in the *obaC gene*	This work
P. fluorescens Δ*obaC* pJH10TS-*obaC*	Δ*obaC* strain carrying a WT copy of the *obaC* gene as a *Bmt* I-*Kpn* I fragment cloned into pJH10TS for complementation by expression *in trans*	This work
P. fluorescens Δ*obaF*	*P. fluorescens* ATCC 39502 with an in-frame truncation in the *obaF gene*	This work
P. fluorescens Δ*obaF* pJH10TS-*obaF*	Δ*obaF* strain carrying a WT copy of the *obaF* gene as a *Bmt* I-*Kpn* I fragment cloned into pJH10TS for complementation by expression *in trans*	This work
P. fluorescens Δ*obaG*	*P. fluorescens* ATCC 39502 with an in-frame truncation in the *obaG gene*	This work
P. fluorescens Δ*obaG* pJH10TS-*obaG*	Δ*obaG* strain carrying a WT copy of the *obaG* gene as a *Bmt* I-*Kpn* I fragment cloned into pJH10TS for complementation by expression *in trans*	This work
P. fluorescens Δ*obaH*	*P. fluorescens* ATCC 39502 with an in-frame truncation in the *obaH gene*	This work
P. fluorescens Δ*obaH* pJH10TS-*obaH*	Δ*obaH* strain carrying a WT copy of the *obaH* gene as a *Bmt* I-*Xba* I fragment cloned into pJH10TS for complementation by expression *in trans*	This work
P. fluorescens Δ*obaI*	*P. fluorescens* ATCC 39502 with an in-frame truncation in the *obaI gene*	This work
P. fluorescens Δ*obaI* pJH10TS-*obaI*	Δ*obaI* strain carrying a WT copy of the *obaI* gene as a *Bmt* I-*Xba* I fragment cloned into pJH10TS for complementation by expression *in trans*	This work
P. fluorescens Δ*obaJ*	*P. fluorescens* ATCC 39502 with an in-frame truncation in the *obaJ gene*	This work
P. fluorescens Δ*obaJ* pJH10TS-*obaJ*	Δ*obaJ* strain carrying a WT copy of the *obaJ* gene as a *BmtI* -*Kpn* I fragment cloned into pJH10TS for complementation by expression *in trans*	This work
P. fluorescens Δ*obaK*	*P. fluorescens* ATCC 39502 with an in-frame truncation in the *obaK gene*	This work
P. fluorescens Δ*obaK* pJH10TS-*obaK*	Δ*obaK* strain carrying a WT copy of the *obaK* gene as a *Bmt* I-*Kpn* I fragment cloned into pJH10TS for complementation by expression in *trans*	This work
P. fluorescens Δ*obaL*	*P. fluorescens* ATCC 39502 with an in-frame truncation in the *obaL gene*	This work
P. fluorescens Δ*obaL* pJH10TS-*obaL*	Δ*obaL* strain carrying a WT copy of the *obaL* gene as a *Bmt* I-*Xba* I fragment cloned into pJH10TS for complementation by expression *in trans*	This work
P. fluorescens Δ*obaM*	*P. fluorescens* ATCC 39502 with an in-frame truncation in the *obaM gene*	This work
P. fluorescens Δ*obaM* pJH10TS-*obaL*	Δ*obaM* strain carrying a WT copy of the *obaM* gene as a *Bmt* I-*Xba* I fragment cloned into pJH10TS for complementation by expression *in trans*	This work
E. coli NiCo21(DE3) pLysS pET28a(+)-*obaG*	Strain expressing the ObaG protein with an *N* -terminal hexahistidine tag	This work
E. coli NiCo21(DE3) pLysS pET28a(+)-*obaH*	Strain expressing the ObaH protein with an *N* -terminal hexahistidine tag	This work
E. coli NiCo21(DE3) pLysS pET28a(+)-*obaI*	Strain expressing the ObaI protein with a *N* -terminal hexahistidine tag	This work

Supplementary Table 2 | Strains and plasmids used in this study.

Primer	Sequence 5'-3'	Function
obaC KOF1 FW	TCGC**GGTACC**GCATAG**TCTAGA**CTGTCGAGAGCCATCACCCATCG	Each pair of KOF (KnockOut Fragment) primers is designed to amplify one upstream (1) and one downstream (2) product, overlapping the *N*- and *C*-termini of each *oba gene* coding sequence. KOF1 is cloned as an *Xba*I-*Avr*II fragment, and KOF2 as an *Avr*II-*Bmt*I, into the suicide vector pTS1.
obaC KOF1 RV	TCGG**GGATCC**CCGACT**CCTAGG**CACGAGAATGCGTGGGGTTGAGC	
obaC KOF2 FW	TGAC**GGATCC**CCGTCA**CCTAGG**CGACTGCTCAACCAGATCGGC	
obaC KOF2 RV	CATG**AAGCTT**CTGGAC**GCTAGC**GGCTGCTCGCAGTGGTAATGG	
obaF KOF1 FW	TCGC**GGTACC**GCATAG**TCTAGA**GGTTGAACATGACGAAGACCGGC	
obaF KOF1 RV	TCGG**GGATCC**CCGACT**CCTAGG**CAGTTGATTGGCCAGTAGCTGGC	
obaF KOF2 FW	TGAC**GGATCC**CCGTCA**CCTAGG**GCCTGCCAGATCGAATACGACG	
obaF KOF2 RV	CATG**AAGCTT**CTGGAC**GCTAGC**GAACGACTCACCGGTCACATCC	
obaG KOF1 FW	TCGC**GAGCTC**GCATAG**TCTAGA**GACACCCTGTCGATCAAGAGCG	
obaG KOF1 RV	TCGG**GGTACC**CCGACT**CCTAGG**GCTCAACCAATCGACGATCTGGG	
obaG KOF2 FW	TCGC**GAGCTC**CCGTCA**CCTAGG**CTGGACGAGGAGTTACTGGCG	
obaG KOF2 RV	CATG**AAGCTT**CTGGAC**GCTAGC**GCGATATGACTGGCGATGCCC	
obaH KOF1 FW	CATCG**GGATCC*****CCTAGG***CTCCTCGGAGGTCAGGTTGATGT	
obaH KOF1 RV	CCATG**AAGCTT*****GCTAGC***CATCGATGAAGCCTGCCAGATC	
obaH KOF2 FW	CATGG**GGTACC*****TCTAGA***CTGATAGGTGAGGTGTTCGCCG	
obaH KOF2 RV	CATCG**GGATCC*****CCTAGG***GTGAAAGGCCAGCAGCACTTGC	
obaI KOF1 FW	CGGC**GGTACC**GATCGA**CCTAGG**CTGCAGGGCGTGCTGAA	
obaI KOF1 RV	GTCC**AAGCTT**CATGTC**GCTAGC**CGGTGCACCAGGTCTGTG	
obaI KOF2 FW	GCCG**GGTACC**GCATGA**TCTAGA**GACGTAGTGGCAATGCTCTTGGCC	
obaI KOF2 RV	CGGC**GGTACC**GCATGA**CCTAGG**GATCAGGTTCATCTGGTCCCGTCG	
obaJ KOF1 FW	TCGC**GGTACC**GCATAG**TCTAGA**CAACTTCACGCTCAGTTCGCCG	
obaJ KOF1 RV	TCGG**GGATCC**CCGACT**CCTAGG**CCAGTCCAACGGTTTGCGTTGC	
obaJ KOF2 FW	TGAC**GGATCC**CCGTCA**CCTAGG**CATATGGCCAGTTGCTCCAGC	
obaJ KOF2 RV	CATG**AAGCTT**CTGGAC**GCTAGC**GACAGCAGAAAGGCCACTACC	
obaK KOF1 FW	TCGC**GGTACC**GCATAG**TCTAGA**CTCAAGTCGTTCGTTCGCCAGC	
obaK KOF1 RV	TCGG**GGATCC**CCGACT**CCTAGG**CTCTTGAGGCGACTCGAGTTCG	
obaK KOF2 FW	TGAC**GGATCC**CCGTCA**CCTAGG**GTGAGCTTCATGGAGCTTGCC	
obaK KOF2 RV	CATG**AAGCTT**CTGGAC**GCTAGC**CTACCGCAGCAATATCCTGCGG	
obaL KOF1 FW	TCGC**GGTACC**GCATAG**TCTAGA**GGCTTAACTGGCACCTGAACC	
obaL KOF1 RV	TCGG**GGATCC**CCGACT**CCTAGG**CAATGCCTGGGCGTTGTTGTCC	
obaL KOF2 FW	TGAC**GGATCC**CCGTCA**CCTAGG**CAGGATATTGCTGCGGTAGTGGC	
obaL KOF2 RV	CATG**AAGCTT**CTGGAC**GCTAGC**CTGCGGTATTGGCAATCAGGAGG	
obaM KOF1 FW	CGTCGG**TCTAGA**CAGACGAACAACGCTCACCC	
obaM KOF1 RV	CGTCGG**CCTAGG**GTCGTCTTCACTGACCAGCC	
obaM KOF2 FW	GCTAGT**TCTAGA**CGTCGG**CCTAGG**GAAGACGCAGTGCCCTTCTTCG	
obaM KOF2 RV	GCTAGT**AAGCTT**CGTCGG**GCTAGC**GCAGGTCGTTGTCCTTGCG	
pJH10TS-*obaC* FW	CCTGAA**GCTAGC**ATGCCTGAATCTCAATTGCTC	Each pair of pJH10TS primers is designed to clone the entire coding sequence of each *oba* gene as either a *Bmt*I-*Kpn*I or *Bmt*I-*Xba*I fragment into pJH10TS.
pJH10TS-*obaC* RV	GAGTCC**GGTACC**TCAAGTCGTGACATAGTTGCG	
pJH10TS-*obaF* FW	CCTGAA**GCTAGC**ATGAGCCTTTCCTCCCCGCATC	
pJH10TS-*obaF* RV	GAGTCC**GGTACC**TCATTGGGTTTTCTCTGCGCTTGAGG	
pJH10TS-*obaG* FW	CCTGAA**GCTAGC**ATGAGCAATGTCAAACAACAAACCGCCC	
pJH10TS-*obaG* RV	GAGTCC**GGTACC**TCATCGTTGGGCTCCCTGATACACC	
pJH10TS-*obaH* FW	CCTGAA**GCTAGC**ATGACCAACCTCCCCTCCACG	
pJH10TS-*obaH* RV	GAGTCC**TCTAGA**GAGGTCATGCCTTGGGCAAGTG	
pJH10TS-*obaI* FW	CCTACG**GCTAGC**ATGTCAGCCTCATTCACCACTGC	
pJH10TS-*obaI* RV	GAGTCC**TCTAGA**TTATTGGCCCAGTTGTACTCGC	
pJH10TS-*obaJ* FW	CCTGAA**GCTAGC**ATGGCAATCCCGAAAATCATGG	
pJH10TS-*obaJ* RV	GAGTCC**GGTACC**TCATCGTGCCTTCCCCAG	
pJH10TS-*obaK* FW	CCTGAA**GCTAGC**ATGACCCAGGGCAAGCTGATCTACG	
pJH10TS-*obaK* RV	GAGTCC**GGTACC**TCATGGCTGCTCGCACTCCGTC	
pJH10TS-*obaL* FW	CCTGAA**GCTAGC**ATGAACCGTTTCGAACATCAGTCGATTGTCG	
pJH10TS-*obaL* RV	GAGTCC**TCTAGA**GTCACTCAAAGGTGGCCCCACCG	
pJH10TS-*obaM* FW	CCTGAA**GCTAGC**GTGAGTCGCCTATCGCC	
pJH10TS-*obaM* RV	GAGTCC**GGTACC**TCATCGAGACTCCCGATAGGC	
pET28a(+)-*obaG* FW	CGTAAC**CATATG**AGCAATGTCAAACAACAAACCG	Each pair of pET28a(+) primers is designed to clone the entire coding sequence of each oba gene as *Nde*I-*Xho*I fragment into pET28a(+). *obaH* was cloned in two steps using an internal *Bam*HI site due to an internal *NdeI* site between the *Bam*HI site and *Xho*I in the *obaH* PCS.
pET28a(+)-*obaG* RV	GAGTCC**CTCGAG**TCATCGTTGGGCTCCCTGATACAC	
pET28a(+)-*obaH* FW	GTCAGC**CATATG**ACCAACCTCCCCTCCACG	
pET28a(+)-*obaH* RV	CACCGC**CTCGAG**TCATGCCTTGGGCAAGTGCTGC	
pET28a(+)-*obaI* FW	CGTAAC**CATATG**TCAGCCTCATTCACCACTGC	
pET28a(+)-*obaI* RV	GAGTCC**CTCGAG**TTATTGGCCCAGTTGTACTCGC	
pTS1 MCS FW	AGCTT**CCTAGG**TAGT**CAATTG**GTCA**CCATGG**TTG**GCTAGC**ACCT**CTCGAG**GCAT**CATATG**	Sense and antisense oligonucleotides for cloning an expanded MCS into pME3087.
pTS1 MCS RV	AGCT**CATATG**ATGC**CTCGAG**AGGT**GCTAGC**CAA**CCATGG**TGAC**CAATTG**ACTA**CCTAGG**A	
sacB FW	AGTCCG**GAATTC**GGAATACGGTTAGCCATTTG	Primers for amplifying sacB from pFLP2, for cloning into pTS1.
sacB RV	AGTCCG**GAATTC**GGATCGATCCTTTTTAACCC	
pJH10TS MCS FW	AATTCTAGT**CAATTG**GTCA**TTAATTAA**CTGC**GCTAGC**ACCT**CTCGAG**GCAT**CATATG**GCA	Sense and antisense oligonucleotides for cloning an expanded MCS into pJH10.
pJH10TS MCS RV	AATTTGC**CATATG**ATGC**CTCGAG**AGGT**GCTAGC**GCAG**TTAATTAA**TGAC**CAATTG**ACTAG	

Supplementary Table 3 | Oligonucleotides used in this study.

Organism	GenBank Accession number	Taxonomy	Function	Phylogeny ref.
Pseudomonas fluorescens ATCC 39502	NA	Proteobacteria	L-TTA (ObaG)	1
Pseudomonas fluorescens ATCC 39502	NA	Proteobacteria	L-TA	2
Pseudomonas fluorescens ATCC 39502	NA	Proteobacteria	L-TA	3
Pseudomonas aeruginosa PA01	NP_254100.1	Proteobacteria	L-TA	4
Pseudomonas aeruginosa PA01	NP_249593.1	Proteobacteria	L-TA	5
Pseudomonas fluorescens SBW25	WP_015886246.1	Proteobacteria	L-TA	6
Pseudomonas fluorescens SBW25	WP_015885469.1	Proteobacteria	L-TA	7
Pseudomonas putida KT2440	NP_742488.1	Proteobacteria	L-TA	8
Pseudomonas protegens Pf-5	WP_011063906.1	Proteobacteria	L-TA	9
Pseudomonas protegens Pf-5	WP_011062763.1	Proteobacteria	L-TA	10
Pseudomonas syringae pv. *tomato* str. DC3000	NP_790259.1	Proteobacteria	L-TA	11
Pseudomonas syringae pv. *tomato* str. DC3000	NP_791665.1	Proteobacteria	L-TA	12
Escherichia coli K12 substr. MG1655	P75823.1	Proteobacteria	L-TA	13
Pseudomonas sp. NCIMB 10558	O50584.1	Proteobacteria	L-TA	14
Ruegeria pomeroyi DSS-3	AAV96391.1	Proteobacteria	L-TA	15
*Bordetella parapertussis*12822	CAE38557.1	Proteobacteria	L-TA	16
Bordetella bronchiseptica RB50	CAE35697.1	Proteobacteria	L-TA	17
Caulobacter crescentus CB15	NP_421887.1	Proteobacteria	L-TA	18
Pseudomonas stutzeri	WP_003292028.1	Proteobacteria	L-TA	19
Desulfobulbus japonicus	WP_028581067.1	Proteobacteria	L-TA	20
Shewanella pealeana	WP_012155441.1	Proteobacteria	L-TA	21
Yersinia enterocolitica	WP_005171133.1	Proteobacteria	L-TA	22
Azorhizobium caulinodans ORS 571	WP_012170545.1	Proteobacteria	L-TA	23
Bosea sp. 117	WP_029351492.1	Proteobacteria	L-TA	24
Methylobacterium sp. GXF4	WP_007566405.1	Proteobacteria	L-TA	25
Burkholderia sp. H160	WP_008921553.1	Proteobacteria	L-TA	26
Geobacter bremensis	WP_026841789.1	Proteobacteria	L-TA	27
Aeromonas jandaei DK-39	O07051.1	Proteobacteria	L-TA	28
Mycobacterium liflandii	WP_015356249.1	Actinobacteria	L-TA	29
Brevibacterium linens	WP_009882170.1	Actinobacteria	L-TA	30
Streptomyces hygroscopicus	WP_014669227.1	Actinobacteria	L-TA	31
Frankia sp. CN3	WP_027140959.1	Actinobacteria	L-TA	32
Micromonospora sp. M42	EWM68484.1	Actinobacteria	L-TA	33
Actinomadura oligospora	WP_026414616.1	Actinobacteria	L-TA	34
Sciscionella marina	WP_020495043.1	Actinobacteria	L-TA	35
Streptomyces sp. SPB78	WP_009070954.1	Actinobacteria	L-TA	36
Glutamicibacter arilaitensis	WP_013349325.1	Actinobacteria	L-TA	37
Demetria terragena	WP_018158110.1	Actinobacteria	L-TA	38
Streptomyces coeruleorubidus NRRL 18370	ADN26256.1	Actinobacteria	L-TA	39
Streptomyces sp. DSM 5940	ADY76671.1	Actinobacteria	L-TA	40
Streptomyces roseosporus NRRL 15998	EFE75624.1	Actinobacteria	L-TA	41
Streptomyces sp. SS	WP_017236989.1	Actinobacteria	L-TA	42
Listeria monocytogenes EGD-e	3PJ0_A	Firmicutes	L-TA	43
Exiguobacterium sibiricum 255-15	3LWS_A	Firmicutes	L-TA	44
Bacillus thuringiensis	WP_000854011.1	Firmicutes	L-TA	45
Bacillus cereus	WP_002014149.1	Firmicutes	L-TA	46
Virgibacillus halodenitrificans	CDQ33605.1	Firmicutes	L-TA	47
Brevibacillus massiliensis	WP_019119750.1	Firmicutes	L-TA	48
Planomicrobium glaciei CHR43	ETP70229.1	Firmicutes	L-TA	49
Thermincola potens	WP_013121253.1	Firmicutes	L-TA	50
Robinsoniella sp. KNHs210	WP_027294918.1	Firmicutes	L-TA	51
Caloramator australicus	WP_008908414.1	Firmicutes	L-TA	52
Desulfotomaculum nigrificans	WP_051410853.1	Firmicutes	L-TA	53
Caldisalinibacter kiritimatiensis	EOD01392.1	Firmicutes	L-TA	54
Thermotoga maritima MSB8	NP_229542.1	Thermotogae	L-TA	55
Capsaspora owczarzaki ATCC 30864	XP_004348623.2	Filasterea	L-TA	56
Phaeodactylum tricornutum CCAP 1055/1	XP_002184057.1	Heterokontophyta	L-TA	57
Calothrix sp. PCC 7507	WP_015127083.1	Cyanobacteria	L-TA	58
Nostoc sp. PCC 7107	WP_015112230.1	Cyanobacteria	L-TA	59

Scytonema hofmanni PCC 7110	WP_017742536.1	Cyanobacteria	L-TA	60
Halothece sp. PCC 7418	WP_015227610.1	Cyanobacteria	L-TA	61
Spirulina subsalsa	WP_017307090.1	Cyanobacteria	L-TA	62
Microcoleus sp. PCC 7113	WP_015182894.1	Cyanobacteria	L-TA	63
Trichodesmium erythraeum	WP_011611295.1	Cyanobacteria	L-TA	64
Rubidibacter lacunae	WP_022605981.1	Cyanobacteria	L-TA	65
Synechococcus sp. PCC 7335	EDX85769.1	Cyanobacteria	L-TA	66
Synechococcus sp. PCC 7336	WP_017325528.1	Cyanobacteria	L-TA	67
Leptolyngbya sp. PCC 7375	WP_006513919.1	Cyanobacteria	L-TA	68
Pleurocapsa sp. PCC 7327	AFY78919.1	Cyanobacteria	L-TA	69
Nodosilinea nodulosa	WP_017302462.1	Cyanobacteria	L-TA	70
Gloeobacter violaceus	WP_011142473.1	Cyanobacteria	L-TA	71
Leishmania major Friedlin	XP_003721570.1	Euglenozoa	L-TA	72
Naumovozyma dairenensis CBS 421	XP_003668398.1	Ascomycota	L-TA	73
Kazachstania naganishii CBS 8797	CCK71098.1	Ascomycota	L-TA	74
Fusarium verticillioides 7600	EWG42458.1	Ascomycota	L-TA	75
Talaromyces marneffei ATCC 18224	XP_002151879.1	Ascomycota	L-TA	76
Candida albicans	O13427.1	Ascomycota	L-TA	77
Saccharomyces cerevisiae S288C	NP_010868.1	Ascomycota	L-TA	78
Eremothecium gossypii ATCC 10985	NP_985913.2	Ascomycota	L-TA	79
Saccharomyces cerevisiae W3031B	AAA72430.1	Ascomycota	L-TA	80
Penicillium aethiopicum IBT 5753	D7PHZ0.1	Ascomycota	L-TA	81
Rhodotorula toruloides NP11	EMS19212.1	Basidiomycota	L-TA	82
Phytophthora parasitica	ETM34049.1	Heterokontophyta	L-TA	83
Phytophthora infestans T30-4	XP_002907740.1	Heterokontophyta	L-TA	84
Halalkalicoccus jeotgali	WP_008418842.1	Euryarchaeota	L-TA	85
Halobacterium sp. DL1	WP_009487011.1	Euryarchaeota	L-TA	86
Thaumarchaeota archaeon SCGC AB-539-E09	EMR73220.1	Euryarchaeota	L-TA	87
Natronolimnobius innermongolicus	WP_007259344.1	Euryarchaeota	L-TA	88
Haloferax sp.	WP_004065464.1	Euryarchaeota	L-TA	89
Haloferax volcanii DS2	WP_004045358.1	Euryarchaeota	L-TA	90
Salinarchaeum sp. Harcht-Bsk1	WP_020446474.1	Euryarchaeota	L-TA	91
Halorubrum lipolyticum	WP_008003501.1	Euryarchaeota	L-TA	92
Thermoplasmatales archaeon SCGC AB-539-C06	WP_004556422.1	Euryarchaeota	L-TA	93
Arabidopsis thaliana Col-0	OAP13027.1	Plantae	L-TA	94
Arabidopsis thaliana Col-0	OAP06494.1	Plantae	L-TA	95
Mus musculus	NP_082195.2	Mammalia	L-TA	96
Pseudomonas putida 24-1	BAD91544.1	Proteobacteria	L-phenylserine aldolase	97
Streptomyces cattelya NRRL 8057	WP_014151017.1	Actinobacteria	L-Fluorothreonine transaldolase	98
Streptosporangium amethystogenes SANK 60709	BAM98975.1	Actinobacteria	L-Thr:uridine-5'-aldehyde transaldolase	99
Sphaerisporangium sp. SANK 60911	BAO20189.1	Actinobacteria	L-Thr:uridine-5'-aldehyde transaldolase	100
Amycolatopsis sp. SANK 60206	AKC92637.1	Actinobacteria	L-Thr:uridine-5'-aldehyde transaldolase	101
Streptomyces sp. SANK 62799	BAJ19052.1	Actinobacteria	L-Thr:uridine-5'-aldehyde transaldolase	102
Streptomyces griseus SANK 60196	BAI23322.1	Actinobacteria	L-Thr:uridine-5'-aldehyde transaldolase	103
Pseudomonas fluorescens ATCC 39502	NA	Proteobacteria	SHMT	104
Pseudomonas fluorescens ATCC 39502	NA	Proteobacteria	SHMT	105
Pseudomonas aeruginosa PA01	NP_254102.1	Proteobacteria	SHMT	106
Pseudomonas aeruginosa PA01	NP_251134.1	Proteobacteria	SHMT	107
Pseudomonas aeruginosa PA01	NP_253292.1	Proteobacteria	SHMT	108
Pseudomonas fluorescens SBW25	WP_015886244.1	Proteobacteria	SHMT	109
Pseudomonas fluorescens SBW25	WP_015885982.1	Proteobacteria	SHMT	110
Pseudomonas putida KT2440	NP_742832.1	Proteobacteria	SHMT	111
Pseudomonas putida KT2440	NP_742489.1	Proteobacteria	SHMT	112
Pseudomonas protegens Pf5	WP_011063571.1	Proteobacteria	SHMT	113
Pseudomonas protegens Pf5	WP_011063904.1	Proteobacteria	SHMT	114
Pseudomonas syringae pv. *tomato* str. DC3000	NP_792241.1	Proteobacteria	SHMT	115
Pseudomonas syringae pv. *tomato* str. DC3000	NP_794383.1	Proteobacteria	SHMT	116
Pseudomonas syringae pv. *tomato* str. DC3000	NP_790310.1	Proteobacteria	SHMT	117
Escherichia coli K12 substr. MG1655	NP_417046.1	Proteobacteria	SHMT	118
Pseudomonas plecoglossicida	WP_013970518.1	Proteobacteria	SHMT	119

Psychromonas ingrahamii	WP_011769815.1	Proteobacteria	SHMT	120
Methylobacterium extorquens AM1	AAA64456.1	Proteobacteria	SHMT	121
Burkholderia pseudomallei	3ECD_A	Proteobacteria	SHMT	122
Salmonella typhimurium	3GBX_A	Proteobacteria	SHMT	123
Campylobacter jejuni	3N0L_A	Proteobacteria	SHMT	124
Rickettsia rickettsii str. Sheila Smith	4J5U_A	Proteobacteria	SHMT	125
Burkholderia cenocepacia J2315	4N0W_A	Proteobacteria	SHMT	126
Escherichia coli SE11	B6I5C4.1	Proteobacteria	SHMT	127
Xanthomonas campestris pv. *vesicatoria* str. 85-10	Q3BXI8.1	Proteobacteria	SHMT	128
Lactobacillus acidophilus NCFM	Q5FMC0.1	Proteobacteria	SHMT	129
Streptomyces clavuligerus	ACJ04032.1	Actinobacteria	SHMT	130
Mycobacterium leprae TN	NP_302318.1	Actinobacteria	SHMT	131
Mycobacterium tuberculosis H37Rv	CCP43846.1	Actinobacteria	SHMT	132
Mycobacterium tuberculosis H37Rv	CCP42793.1	Actinobacteria	SHMT	133
Mycobacterium tuberculosis	3H7F_A	Actinobacteria	SHMT	134
Corynebacterium glutamicum ATCC 13032	NP_600221.1	Actinobacteria	SHMT	135
Streptomyces coelicolor A3(2)	NP_629503.1	Actinobacteria	SHMT	136
Bacillus subtilis subsp. *subtilis* str. 168	NP_391571.1	Firmicutes	SHMT	137
Geobacillus stearothermophilus	1KKJ_A	Firmicutes	SHMT	138
Streptococcus thermophilus	4WXG_A	Firmicutes	SHMT	139
Lactococcus lactis subsp. *lactis* Il1403	NP_266757.1	Firmicutes	SHMT	140
Candidatus Desulforudis audaxviator MP105C	B1I6M4.1	Firmicutes	SHMT	141
Thermoanaerobacter sp. X514	B0K631.1	Firmicutes	SHMT	142
Hydrogenobacter thermophilus TK-6	BAI70276.1	Aquificales	SHMT	143
Thermus thermophilus	WP_011228734.1	Deinococcus-Thermus	SHMT	144
Synechococcus elongatus PCC 6301	Q5N2P9.2	Cyanobacteria	SHMT	145
Thermosynechococcus elongatus BP-1	Q8DH33.1	Cyanobacteria	SHMT	146
Plasmodium falciparum 3D7	4O6Z_A	Apicomplexa	SHMT	147
Plasmodium vivax Sal-1	XP_001613892.1	Apicomplexa	SHMT	148
Saccharomyces cerevisiae S288C	P37291.2	Ascomycota	SHMT	149
Talaromyces marneffei ATCC 18224	XP_002151718.1	Ascomycota	SHMT	150
Aspergillus niger	CAK48606.1	Ascomycota	SHMT	151
Homo sapiens	3OU5_A	Mammalia	SHMT	152
Oryctolagus cuniculus	1LS3_A	Mammalia	SHMT	153
Mus musculus	AAK15040.1	Mammalia	SHMT	154
Homo sapiens	1BJ4_A	Mammalia	SHMT	155
Paracoccus sp. AJ110402	BAG31000.1	Proteobacteria	MSHMT	156
Aminobacter sp. AJ110403	BAG31001.1	Proteobacteria	MSHMT	157
Streptomyces vinaceusdrappus NRRL 2363	AEF16058.1	Actinobacteria	G/SHMT	158
Streptomyces sp. Sp080513GE-23	BAP16701.1	Actinobacteria	A/MSHMT	159
Streptomyces sp. Sp080513GE-23	BAP16692.1	Actinobacteria	A/MSHMT	160
Streptomyces sp. SANK 60405	BAJ05887.1	Actinobacteria	SHMT	161
Streptomyces sp. NRRL 30471	ADZ45329.1	Actinobacteria	SHMT	162
Streptomyces sp. SN-1061M	ADC96660.1	Actinobacteria	SHMT	163
Streptomyces sp. MK730-62F2	ACQ63622.1	Actinobacteria	GHMT	164

Supplementary Table 4 | Source organisms and GenBank Accession numbers for SHMT, L-TA, L-TTA and AHBAS amino acid sequences used in phylogenetic analyses.

SUPPLEMENTARY NOTE 1 | pTS1 suicide vector construction. The pTS1 vector (Figure S2; Table S2) (GenBank accession no. KX931445) was constructed by introducing a novel polylinker sequence into the multiple cloning site (MCS) of the ColE1-based suicide vector pME3087[2] (GenBank accession no. KX931444) to facilitate greater ease of cloning. Sense and antisense oligonucleotides (Table S3) comprising six additional restriction sites (*Avr*II-*Mfe*I-*Nco*I-*Bmt*I-*Xho*I-*Nde*I) were ligated together and cloned into the pME3087 *Hin*dIII site, which was retained upstream and removed downstream of the MCS. *Xba*I, *Avr*II and *Bmt*I do not cut within the obafluorin BGC and so could always be used for cloning PCS flanking sequences for knockouts, also allowing easy subcloning between knockout vectors to create different combinations of knockout flanking sequences subsequently. The vector was further modified by introduction of the *sacB* gene and upstream promoter region, amplified as a 2009 bp *Eco*RI fragment from the site-specific excision vector pFLP2[3]. The *Bacillus subtilis sacB* gene encodes a levansucrase that is fatal to cells transformed with it when in the presence of sucrose, and is a powerful tool for counter-selection[4]. Only successful clones possessing the *sacB* fragment oriented in the direction of transcription away from the pME3087 MCS were selected to prevent unwanted read-through. An unwanted *Nde*I site introduced in the *sacB Eco*RI fragment was removed by partial digestion and religation following treatment with Klenow DNA Polymerase (Invitrogen), leaving a single site in the MCS.

SUPPLEMENTARY NOTE 2 | pJH10TS expression vector construction. *Δoba* strains were genetically complemented using pJH10TS (Table S2), a modified *Eco*RI-*Mfe*I-*Pac*I-*Bmt*I-*Xho*I-*Nde*I-*Kpn*I-*Cla*I-*Xba*I-*Sac*I) version of pJH10[5], kindly supplied by Prof. Christopher Thomas (University of Birmingham). Sense and antisense oligonucleotides (Table S3) comprising five additional restriction sites (*Mfe*I-*Pac*I-*Bmt*I-*Xho*I-*Nde*I) were ligated together and cloned into the pJH10 *Eco*RI site, which was retained upstream and deleted downstream of the MCS. pJH10 is a broad range IncQ expression vector that has been shown to be constitutively expressed in *P. fluorescens*[6].

Supplementary Note 3. Trimmed sequences used in the Maximum Likelihood phylogeny (Figure 5.). Number corresponds to phylogeny reference number allocated in Supplementary Table 4.

```
>1
VDWLSSTLGKDHQYREDSLSLTANENYPSALVRLTSGSTAGAFYHCSFPFEVPAGEWHFP
EPGHMNAIADQVRDLGKTLIGAQAFDWRPNGGSTAEQALMLAACKPGEGFVHFAHRDGGH
FALESLAQKMGIEIFHLPVNPTSLLIDVAKLDEMVRRNPHIRIVILDQSFKLRWQPLAEI
RSVLPDSCTLTYDMSHDGGLIMGGVFDSPLSCGADIVHGNTHKTIPGPQKGYIGFKSAQH
PLLVDTSLWVCPHLQSNCHAEQLPPMWVAFKEMELFGRDYAAQIVSNAKTLARHLHELGL
DVTGESFGFTQTHQVHFAVGDLQKALDLCVNSLHAGGIRSTNIEIPGKPGVHGIRLGVQA
MTRRGMKEKDFEVVARFIADLYFKKTEPAKVA

>2
IDLRSDTVTQPTPGMLDAMASAVSGDDVYGEDPSVNQLEAELARRLGFDAALFVPTGTMS
NLLALMAHCERGEEYIVGQQAHTYKYEGGGAAVLGSIQPQPLEVQADGSLDLSQVLDAIK
PDDFHFARTRLLALENTMQGKVLPMTYLAKARAFTREHGLALHLDGARIYNAAVKLGVDA
REIAGHFDSVSVCLSKGLGAPVGSVLCGSTALIAKARRLRKMVGGGMRQAGSLAAAGLYA
LDHQVQRLADDHANAQWLGDELRQAGYTVEPVQTNMVYVQMGDRAQALKSFAAERGIKLS
AAPRLRMVTHLDVSRAQIEQVVQTFVAFSQK

>3
MTDKSQQFASDNYSGICPEAWAAMEQANQGHQRAYGDDEWTHRAADGFRNLFETDCEVFF
AFNGTAANSLALSSLCQSYHSVICSETAHVETDECGAPEFFSNGSKLLTARTENGKLTPE
SIREIALKRQDIHYPKPRVVTLTQATEVGSVYTPEEIRAISVTCKELGLNLHMDGARFSN
ACAFLGCSPADLTWKAGVDVLCFGGTKNGMAVGEAILFFNHKLAEDFDYRCKQAGQLASK
MRFLSAPWVGLLENDAWLKHARHANHCAQLLSSLVADIPGVELMFPVQANGVFLQLSEPA
IAALTAKGWRFYTFIGKGGARFMCAWDTEEERVRELAADIREVMNA

>4
MTDHTQQFASDNYSGICPEAWAAMAEANRGHERAYGDDQWTARASDYFRQLFETDCEVFF
AFNGTAANSLALAALCQSYHSVICSETAHVETDECGAPEFFSNGSKLLLAQTEVGKLTPA
SIRDIALKRQDIHYPKPRVVTLTQATEVGTVYRPDELKAISATCKELGLHLHMDGARFSN
ACAFLGCSPAELSWKAGVDVLCFGGTKNGMAVGEAILFFNRDLAEDFDYRCKQAGQLASK
MRFLAAPWVGVLQDDAWLRYADHANRCARLLAELVADVPGVSLMFPVEANGVFLQLSEPA
IEALRARGWRFYTFIGEGGARFMCSWDTDIERVRELARDIRLVMGA

>5
IDLRSDTVTQPTAGMREAMAAAELGDDVYGEDPTVNRLEAELAARLGFAAALFVPTGTMS
NLLGLMAHCERGDEYIVGQQAHTYKYEGGGAAVLGSIQPQPIDGEADGSLHLDKVAAAIK
ADDFHFARTRLLALENTMQGKVLPLDYLAAARAFTRARGLALHLDGARLYNAAVKLGVDA
SEITRHFDSVSVCLSKGLGAPVGSVLCGSVELIGKARRWRKMVGGGMRQAGLLAAAGLYA
LDHQVARLADDHANAARLGDGLRELGYAVEPVQTNMVYVDVGERAVALRDFLAERGVRIS
AAARLRLVTHLDVSAESIGQVLDAFAAFRRS

>6
MTDKSQQFASDNYSGICPEAWAAMEQANQGHQRAYGDDEWTHRAADGFRNLFETDCEVFF
AFNGTAANSLALSSLCQSYHSVICSETAHVETDECGAPEFFSNGSKLLTARTENGKLTPE
SIREIALKRQDIHYPKPRVVTLTQATEVGSVYTPEEIRAISVTCKELGLNLHMDGARFSN
ACAFLGCSPADLTWKAGVDVLCFGGTKNGMAVGEAILFFNHKLAEDFDYRCKQAGQLASK
```

MRFLSAPWVGLLENDAWLKHARHANHCAQLLSSLVADIPGVELMFPVQANGVFLQLSEPA
IAALTAKGWRFYTFIGKGGARFMCAWDTEEERVRELAADIRKVMEA

>7
IDLRSDTVTQPTPGMLDAMATAASGDDVYGEDPSVNRLEAELAKRLGFAAALFVPTGTMS
NLLALMAHCERGEEYIVGQQAHTYKYEGGGAAVLGSIQPQPLDVQADGSLDLDQVLAAIK
PDDFHFARTRLLALENTMQGKVLPLEYLANARAFTREHGLALHLDGARLYNAAVKLGVDA
RAIAQHFDSVSVCLSKGLGAPIGSVLCGSTALIAKARRLRKMVGGGMRQAGSLAAAGLYA
LDHQVQRLADDHANALWLGDALREAGYSVEPVQTNMVYVQIGDRADALKAFAAERGIKLS
VAPRLRMVTHMDVSRAQMERVLQAFVEFSPK

>8
MTDKSQQFASDNYSGICPEAWVAMEKANRGHDRAYGDDQWTERASEYFRNLFETDCEVFF
AFNGTAANSLALASLCQSYHSVICSETAHVETDECGAPEFFSNGSKLLTAASVNGKLTPQ
SIREVALKRQDIHYPKPRVVTITQATEVGTVYRPDELKAISATCKELGLNLHMDGARFTN
ACAFLGCSPAELTWKAGVDVLCFGGTKNGMAVGEAILFFNRQLAEDFDYRCKQAGQLASK
MRFLSAPWVGLLEDGAWLRHGNHANHCAQLLASLVSDLPGVELMFPVEANGVFLQMPEHA
IEALRGKGWRFYTFIGSGGARFMCSWDTEEARVRELAADIRTIIGG

>9
MTDKSQQFASDNYSGICPEAWAAMEQANHGHERAYGDDQWTARASDHFRKLFETDCEVFF
AFNGTAANSLALSSLCQSYHSVICSETAHVETDECGAPEFFSNGSKLLIAGTQNGKLTPE
SIREIALKRQDIHYPKPRVVTLTQATEVGSVYTPEEVRAISATCKELGLNLHMDGARFSN
ACAFLGCTPAELTWKAGVDVLCFGGTKNGMAVGEAILFFNHKLAEDFDYRCKQAGQLASK
MRFLSAPWVGLLENDAWLKHARHANHCAQLLAQLVADIPGVELMFPVQANGVFLQLSEPA
IAALTAKGWRFYTFIGKGGARFMCSWDTEEDRVRELAADIRTVMSA

>10
IDLRSDTVTQPTPGMLDAMASAPTGDDVYGEDPTVNRLEGELARRLGFAAALFVPSGTMS
NLLALMAHCERGDEYIVGQQAHTYKYEGGGAAVLGSIQPQPLELQADGSLDLVQVAAAIK
PDDFHFARTRLLALENTMQGKVLPLAYLAQARAFTREHGLALHLDGARLYNAAVKLGVDA
RVIAEHFDSVSVCLSKGLGAPVGSVLCGSVELIAKARRLRKMVGGGMRQAGGLAAAGLYA
LDHQVQRLADDHAHAQLLADGLRRVGYSVEPVQTNMVYVEMGDRAEALKAFAAERGIKLS
AASRLRLVTHLDVSAGQIEHVLSTFAEFSRN

>11
MTDQSQQFASDNYSGICPEAWAAMEQANKGHQRAYGDDQWTARASDDFRRLFETDCEVFF
AFNGTAANSLALSSLCQSYHSVICSETAHVETDECGAPEFFSNGSKLLTARSPGGKLTPE
SIREVALKRQDIHYPKPRVVTLTQATEVGGVYQPEELKAISATCKELGLHLHMDGARFSN
ACAFLDASPAELTWKSGVDVLCFGGTKNGMAVGEAILFFDHDLAVDFDYRCKQAGQLASK
MRFLSAPWVGLLENDAWLKHARHANRCAQLLADLVKDIAGVELMFPVQANGVFLQLSEPA
IAALTARGWRFYTFIGNGGARFMCSWDTEEDRVRELAADIRLVMQG

>12
IDLRSDTVTLPTAGMLDAMAHAPVGDDVYGEDPTVNLLEATLAERLGFDAALFVPSGTMS
NLLALMAHCERGDEYIVGQQAHTYKYEGGGAAVLGSIQPQPLDVQADGSLSLQQVLDTIK
PDDFHFARTRLLALENTMQGKVLPLEYLAAARKLTLEKGLALHLDGARLYNAAVKLGVDA
REITRHFDSVSICLSKGLGAPIGSVLCGSQPLMVKARRLRKMLGGGMRQAGGLAAAGLYA
LDHQVQRLADDHANAAFLAESLSGLGYSVEPVQTNMVYVQIGDRAAALKAFCAERGIVLT
AASRLRMVTHLNVSRAQAEQVIAAFAAFEHP

>13
IDLRSDTVTRPSRAMLEAMMAAPVGDDVYGDDPTVNALQDYAAELSGKEAAIFLPTGTQA
NLVALLSHCERGEEYIVGQAAHNYLFEAGGAAVLGSIQPQPIDAAADGTLPLDKVAMKIK
PDDIHFARTKLLSLENTHNGKVLPREYLKEAWEFTRERNLALHVDGARIFNAVVAYGCEL
KEITQYCDSFTICLSKGLGTPVGSLLVGNRDYIKRAIRWRKMTGGGMRQSGILAAAGIYA
LKNNVARLQEDHDNAAWMAEQLREAGADVMRQDTNMLFVRVGEENAAALGEYMKARNVLI
NASPIVRLVTHLDVSREQLAEVAAHWRAFLAR

>14
MTDQSQQFASDNYSGICPEAWAAMEKANHGHERAYGDDQWTARAADHFRKLFETDCEVFF
AFNGTAANSLALSSLCQSYHSVICSETAHVETDECGAPEFFSNGSKLLTARSEGGKLTPA
SIREVALKRQDIHYPKPRVVTITQATEVGSVYRPDELKAISATCKELGLNLHMDGARFSN
ACAFLGCTPAELTWKAGIDVLCFGGTKNGMAVGEAILFFNRKLAEDFDYRCKQAGQLASK
MRFLSAPWVGLLEDGAWLRHAAHANHCAQLLSSLVADIPGVELMFPVEANGVFLQMSEPA
LEALRNKGWRFYTFIGSGGARFMCSWDTEEARVRELAADIRAVMSA

>15
MFFASDNSGPVHPEILAGLTDANQGYAMAYGADTQMQAVQEKIRRIFEAPDAAVYLVATG
TAANSLALATLSQPWQTIFCSPVAHIHEDECNAPEFYSGGAKLTLVPGGDRMTPEALRAS
ITGEETRGVHGPQRGPVSITQVTERGGVYSIAELQALCAVAKAYGLPVHLDGARFANALV
ALNASPAEMTWKAGVDAVSFGGTKNGCMGVEAVIFFDPKHAWEFELRRKRGAHLFSKHRY
LSAQMLAYLSDDLWLRSARRANDNCARLAEGLRAAGAEFLHEPQANIVFASFPRAIHRRL
MGAGASYHLWGAELEGANEDEMLACRMVCDWSIGTDQIDRFLSL

>16
IDLRSDTVTRPSAAMRQAMAAAPVGDDVMGDDPSVRRLQDEVAARAGKEAGLFFPSGTQS
NLAALMAHCARGDEYLVGQQAHTYKFEGGGAAVLGSIQPQPVDHAADGSLPLDRLAAALK
PGGDPHFARTRLLALENTFQGRVMPAGYVAQATGWAREHGLSTHLDGARVFNAAVASGVP
VQQLCEPFDTVSICFSKGLGAPVGSVLVGSRALIEQAHRWRKMLGGGMRQAGILAAACLH
ALHHHVERLALDHDNAARLAAGLDGIDGVRVLGQHTNMVFAEFDPARCESLTTALRGQGI
LMRAVYGGPTRLVTHLDVDADGIDRVVDAVRGHCAAAR

>17
IDLRSDTVTRPSAAMRQAMAAAPVGDDVMGDDPSVRRLQDEVAARAGKEAGLFFPSGTQS
NLAALMAHCARGDEYLVGQQAHTYKFEGGGAAVLGSIQPQPVDHAADGSLPLDRLAAALK
PGGDPHFARTRLLALENTFQGRVMPAGYVAQATGWAREHGLSTHLDGARVFNAAVASGVP
VQQLCEPFDTVSICFSKGLGAPVGSVLVGSRALIEQAHRWRKMLGGGMRQAGILAAACLH
ALHHHVERLALDHDNAARLAAGLDGIDGVRVLGQHTNMVFAEFDPARCESLTTALRGQGI
LMRAVYGGPTRLVTHLDVDADGIDRVVDAVRGHCAAAR

>18
MTQTAPRYDFASDNVAGAMPEVMEALIAANAGTASGYGTDHVSRAAADRIRAALDADAQV
RFTASGTAANAFALTLLAQPHEAVLAHEHAHICTDETGAPGFFGQGVGLIGLPGASGKME
LAALEAALAQPDVSYRQPAAALSLTTATEYGTVYSEDHLRALIAPVKAKGYGVHLDGARL
ANAVAGGFDLKSIAKMGVDILVMGGTKAGSTPTEAVVFLNPDHAKRLDARLKHAGQLISK
GRFLAAPWLGLLGENGQTAPWAARAAHANAMAQKLAALMPVPIKHPVEANGIFVEMDELA
LERLRGEGWFVYRFLDGTVRFMCSWATTPEMVEDLGAALKRVA

>19

IDLRSDTVTQPTTAMREAMLGAETGDDVYAEDPTVSRLECRLAADLGFAAGLFVPSGTMS
NLLGLMAHCERGDEYIVGQQAHTYKYEGGGAAVLGSIQPQPIEMEADGTLDLARVEAAIK
PDNFHFARSRLLALENTMQGKVLPLEYLAAARELSQRHGLALHLDGARLFSAAVKLGCDA
REITRHFDTVSVCLSKGLGAPVGSVLCGSDAFIAKARRLRKMVGGGMRQAGILAAAGLYA
LEHNIKRLGDDHRRAEWLGNELAALGFSVEPVQTNMVYVDMGDQAAALTAFCAQRGIRLT
SGSQLRLVTHMDVHDEHVPVVVSAFAEFAENMS

>20
IDLRSDTVTPPTDAMRRVMADAVVGDDVYGEDPTIRELEEYCADLLGKEAALFTTSGTQG
NLLGIMVHCQRGDEYIVGQQAHAYRYEAGGAAILASVQPQPLNFESDGTLDLKKVTAAIK
PDDVHFARTRLLCLENTHNGMALPLGYVQEAAKVAAMNQLKLHLDGARLFNAAVFLDVPV
KQLVQGVDTVSVCLSKGLGAPVGSLLCGSYQAIHEARKWRKMLGGGLRQAGILAAAGLYA
LQQHVSRLQEDHTHAALLAQGLTGIPGIEVQFGKLQTNMVFIKTSEQHARGLSQYLKEHN
ILIGTGNPIRLVTHLGISGKDIEQVIACCRAFFKN

>21
IDLRSDTVTQPCQNMRALMASAETGDDVYGEDPSVKQLESYAAELLNKQAAVFCPSGTQS
NLMGLLSHCGRGDEYIVGNVAHTYLYEGGGAAVLGSIQPQPLTLQADATMDLSELEKAIK
PKDVHYARTKLICLENTHGGLPLPDGYTQNVKAIAQRHGLSMHLDGARLFNAVVAGNKSA
ATLTADFDSVSICLSKGLGAPVGSLLVGSNKFIDEARHWRKMLGGGMRQAGIIAAAGLYA
LQNNVSRLSDDHRRASELANALGQIDGVSIPLGQANTNMLYVSVSQAMRERLAERAANYN
ILLPAGEQMRLVTHLNINDDDLKAIIELFQQAA

>22
IDLRSDTVTQPDAAMRQAMANAEVGDDVYGDDPTVNALEAQAARLSGKEAALFLPTGTQA
NLVALLTHCQRGEEYIVGQKAHNYLYEAGGAAVLGSIQPQPIDANDDGTLPLDKVLAAIK
PDDIHFAQTRLLSLENTHSGKVLPLSYLQQAWALTREQKLALHIDGARIFNAAVALNVPL
SEITQYCDTLTICLSKGLGAPVGSLLCGSAEYIKRARRWRKMTGGGMRQAGILAAAGLYA
LEHNVARLKDDHDNALWLEQQLRALGVEIVAPGAQTNVLYIQQSSELAAKLGPWMRERGV
LISAGPITRMITHININRTDLEKVVALWREFLSEQR

>23
QQSQPQQFASDNYAGICPEAWAAMAEANEGSAVAYGDDDWTHRASDAFRALFQTNCEVFF
AFNGTAANSLALASLCQSYHSVICAASAHVETDECGAPEFFSNGSKLLVATTDGGKLTPD
AVLALATGRSDIHYPKPRAVTITQPTETGQVYTLDEIAALSATCRDLGLRLHMDGARFAN
ACAALGCSPAEMTWKVGVDVLCFGGTKNGMAVGEAILFFDRALALDFDYRCKQAGQLASK
MRFLSAPWVGVLESGAWLRNAAHGNACARRLAAAIADVSGIEPMFPVEANAVFLRAPEAV
LQGLRENGWRFYTFIGGGARFMFAWDADLARVDQLAADIRAIAA

>24
MTDAQQFASDNYAGICPEAWAAMEAANRGSAPAYGDDEWTTQASDAFRALFETPCEVFFA
FNGTAANSLALASLCQSYHSVICASSAHVETDECGAPEFFSNGSKLLIVPTPDGKLTPDA
VRALAVSRTDIHFPKPRVVTITQPTETGQVYTVEEVRALSAMCRELGLKLHMDGARFAHA
CAALGCAPADITWKAGVDVLCFGGTKNGMAVGEAVLFFDPALAQDFDYRCKQAGQLASKM
RFLSAPWVGLLGSGAWLANARHANDCAARLAAAVAGLPEIELMFPVEANGVFLRAPEPVL
EALRAKGWRFYTFIGGGARFMFAWDADPVRVDALADDIRAACAG

>25
QHDDSQQFASDNYSGICPEAWSAMEAANRGHAPAYGEDAWTARAADAFRRLFETECEVFF
AFNGTAANALALAALCQSYHSVICADSAHVETDECGAPEFFSNGSKLLTARTEGGKLTPE

AIRGLATNRSDIHFPRPRVVTITQPTETGQVYTLAELRALSATCRDLGLALHMDGSRFAN
ACASLGCSPADMTWRAGIDVLCFGGTKNGMHAGEAVVFFDPKLAEDFGFRCKQAGQLASK
MRFLSAPWVGMLESGAWLRNGAHGNACARRFADAVAGLPGIRALFPVEANAVFLAMPAVT
MERLRARGWRFYTFIGGGARFMFAWDAETARVDALVADLRALAAE

>26
MQHFASDNYAGICPEALDALIAANNSGHEPAYGDDSWTNQVCDRLRDLFQTDCEVFFVFN
GTAANSLALASLCQSYHSVICHELAHIETDECGGPEFFSGGSKLLTAPDIGGKLTPDAIE
SIVTRRADIHYPKPKVVTLTQSTEVGTVYRVEEVRAIAAIAKRRHLKVHMDGARFANAVA
ALDVHPSEITWRAGVDVLCFGGTKNGLPVGEAVVFFDRALADDFAYRLKQAGQLASKMRF
ISAPWLGLLDNDVWLRNARHANAMARLLETRLQEIPGVSIMFPSESNAVFAQLPPPAAKA
MRARGWKFYEFIGAGGCRLMCAWDTQPDTVERFAAEVRELCAA

>27
MKHHFASDNYAGICNEAWAAMAEANRGMASSYGDDYWTAEACEKIRELFETDCEVFFVFN
GTAANSLALASLCQSYHSIVCHEMAHIETDECGASEFFSNGTKVLLVPGENGKIDLDAVE
HTIHKRTDIHYPKPKALSITQATELGTLYSVQELQAIGELAKKHSLRVHMDGARFANAVA
SLNVAPKEISWQAGVDVLTFGGTKNGFAIGEAVVFFNKELAFEFDYRCKQAGQLASKMRF
LTAPWIGMLESGAWLKNAAHANNCARLLENEIRKIPQVRVMFPSQANSVFLEMAPEALEA
LRGRGWHFYTFIGSGGARFMCSWDTDTAEVANLVADIKASVA

>28
IDLRSDTVTQPTDAMRQCMLHAEVGDDVYGEDPGVNALEAYGADLLGKEAALFVPSGTMS
NLLAVMSHCQRGEGAVLGSAAHIYRYEAQGSAVLGSVALQPVPMQADGSLALADVRAAIA
PDDVHFTPTRLVCLENTHNGKVLPLPYLREMRELVDEHGLQLHLDGARLFNAVVASGHTV
RELVAPFDSVSICLSKGLGAPVGSLLVGSHAFIARARRLRKMVGGGMRQAGILAQAGLFA
LQQHVVRLADDHRRARQLAEGLAALPGIRLDLAQVQTNMVFLQLTSGESAPLLAFMKARG
ILFSGYGELRLVTHLQIHDDDIEEVIDAFTEYLGA

>29
IDLRSDTVTRPSQAMLAAMTAAEVGDDVWGDDPTVLSLQVAVADRAGKEAGLFFPSGTQS
NLAAVMAHCERGDEYLVGQLAHSYKYEGGGAAVLGSIQPQPIENAPDGTLPLEKIADAIK
PIDNHFARTRLLALENTINGRVLPEDYVQEAVALVRSRGLSAHLDGARVCNASVASGRPV
ADLCAPYDTVSICFSKGLGAPVGSVLVGSTLLIERAHRWRKVLGGGMRQSGILAAACLYA
LEHNVERLADDHANARHLAEGLSSIEPVKVLSHATNMVFAQFPEADRAPLEAWLKQRGIL
TQMLYASRFVTHCDVSRNDVDTFVESVGDYFAQRR

>30
VDFRSDTVTTPSAGMRRAMADAEVGDAVYDEDPTTNRLESMAAEILGKEAGLFFPSATQA
NLAGIMAHCGRGDEYIVGQTAHAYRDEGGGAAVLGSVQPQPLPNEPDGIIDLDAITSAIK
PDDFHNPITRLLTLENTFNGLPLPADYIEGATELARSHGLATHLDGARVMNAAVATGADP
SAIADRFDSVSLCLSKGLGAPVGALLVGSKELVGRAKRIRKTLGGGMRQTGVLAAAGIFA
LERNVDRMREDHDRAKRLAEIFADFPELGAGEARTNMVFLSPDGVDMDAFSTFFADRDII
TGSAHGKLRWVTHLDIDDEAIERVRRACKEFFGR

>31
VELRSDTFTLPTARMLEEMTRAPLGDDVYGEDPTARELEELAASLLGKEDACFMPSGTMG
NLAAILAHCPRGTKALAGAESDIYVYEAGGASYCGGVIVEPVANAADGTMALADIEAAFP
EDPEDPQFALPALLCVENPQNRCGGRVLPLEYLAGVQALARARKVAVHLDGARIFNAALA
LDVPVRVIAGYADSVQFCLSKGLSAPVGSMVVGSADFIRSVRRIRKMLGGGMRQAGVLAA

AGLVALREMTARLGDDHRTARRLAEGLAALPGVVVEPVETNMVFFRVDTPHLTQAEFIER
SWNEGVRLAELGRGRIRAATHAGVSEAGVDRALSVFRDILAPAA

>32
IDLRSDTVTRPTADMRRAMAEAEVGDDVYGEDPSVRALEEHTADLLGHEAAVFVPSGTMG
NFCALRASAEIGTEIIADAEAHIVTYELGGLAALGGVQTRTLPGLAGPLDLAELAAQIRP
RTAPKTYNMVPTSVVAVENTQARAGGRIWPLDRLERLREITDDAGVVLHCDGARIWNAAV
GLGVEPRRLGELFGTLSVCLSKGLGAPVGSLVVGDAEHIDRARVWRKRLGGGMRQAGVLA
AAGLYALRHHLDRLADDHRRAAELAATLADAAPGRVEPKLVETNMIFVSVPDAEDFAARA
AAAGVLVGTSGPTSLRLLTHLDVDDDDIRAAGAVLARLLTA

>33
IDLRTDTVTRPTPGMREAMAAAEVGDDVYGEDPTVTALEAEVAALFGHEAALFAPSGSMA
NQIALQLHVPPGDELLCDADAHVVTYEVGAAAAYGGISSRTWPAVGADLDPDVVAGMIRP
DGYFAVPTRAIAVEQTHNRGGGGVIPLPTLRRMREVADEHGVALHCDGARIWHAHVADRV
PLAGYGELFDTLSVCLSKGLGAPVGSLVVGSAEKIARARVLRKRMGGGMRQVGILAAAGR
YALAHHVERLAEDHAKAARLAEAVAPHGVLASVVRTNIVPLDLTKHPLDAHALAAAAREA
GLLISVLGPRTARLVTHLDVTDEQIDSAATILDTALRA

>34
LRAVHHGFGSDNHAGVHPEVMAALAEANTGYQIAYGSDVWTERLDRVIERHFGPQATAFP
VFNGTGANVVGLQAMCERWSSVICAESAHINQDECGASEHVGGLKLIPVPAENGKLTPEA
VARRATGFGNVQHSQPAVVSITQVTEVGTVYTPDEIVALASCAHERGMFLHMDGARLANA
AVSLDVPLRAITTDVGVDVLSFGGTKNGMMLGEMVVVLNQDAAHGSAYLRKSSMQLASKM
RFLSAQFVALLDGDLWRRNARHANAMARLLAEQVRNIPGVEIVRPVDANAVFARLPERAA
EHLRERFSFHGHPSEEVRWMCAFDTTEADVTAFTTALHKSMTA

>35
HDPEYRGFASDNYSGVHPEIMAALATANGGHQIAYGEDDYTEAVQQVFQSHFGPGAEAFP
VFNGTGANVVGLQSLTERWQSVICTETAHIHVDECGAPERMGGLKLLTVSTPDGKLTPEL
VDTEAYGFDDEHRAQPRVVSITQSTELGTCYTPAEVAALCEHAHARGMVVHMDGARLSNA
AAHLGVPFAEFTTEAGVDVLSFGGTKNGMMFGEAVVVLNHEAARGLPYLRKMSMQLASKM
RFVSAQFDALLSGDLWLRSATHANAMAARLAGTVRDVPGLRLTREPQANEVFAILPKDVT
ARLQKRFRFYTWDEETGEVRWVTTFDTTEADIDTFAAAVREELQA

>36
HDPAIRGFASDNYAGAHPEVLAALAVANGGHQVAYGGDAYTEHLQVIMRDLFGPRAEAFP
VFNGTGANVVSLQAVTDRWGAVICADSAHIHVDEGGAPERVGSLKLLTVPTPDGKLTPEL
IDREAYGWDDEHRAMPQAVSITQSTELGTLYTPDEIAAICAHAHERGMKVHLDGSRLANA
AAALGVPVKAFTTDAGVDLLSFGGTKNGMVFGEAVVVLDPDGIRHMKHLRKLSMQLASKM
RFVSAQFEALLADGLWLRSAAHANAMAQRLAAGVRGIDGVEILYPVQANGVFARLPHEVT
ERLQKRYRFYYWDEAAGSVRWMCAFDTTEDDVDGFLAALREEMTR

>37
HDISVRGFASDNYSGVHPRVMDAIARANGGHQIAYGEDHYTEELGNVARNVFGPEAQIFP
VFNGTGANVTALQSLLPRWGAVICASSAHINVDENGAPERVGGFKLLQVDTPDGKLTPEL
IDKEAWGWGDEHRAQPLAVSITQATELGTIYTPEEIKAITDHAHKLGMNVHLDGSRLSNA
AASLNVPLRALTTDAGVDIVSLGGTKNGILLGEGILTLRSELAADLKYLRKMNMQLSSKM
RFISAQLIELYGTELWRELASHSNAMAAKLSQAVGDIEGVELVYPTQANGVFAQLPTEIS
DQLREHFRFYDWDRAAGQVRWMCSFDTTEEDVEAFISKLRELCGA

>38
HDTESRGFASDNYAGIHPEILTAIERANGGHQVAYGGDDYTAALQDVIRSHFGEAAQAFP
VFNGTGANVVALQAVTDRWDGVVCTTAAHINVDECAAPERMGGLKLLQVETSDGKLRPDQ
LDAFALKHDDEHAAQARVVSITQTTELGTCYSVEEIAAVSRRAHDLGMVVHVDGSRLSNA
AASLGVGFREMVTDTGVDLLSFGGTKNGLMVGEAVIVLNPDAVRGMKFLRKQTMQLASKM
RFISAQLMALLSDDLYLRNASHANAMAQRLAEGVRDLPGVSIARPVQSNSVFPVLPHEVS
RRLMESFLFYFWDETAGEVRWMCAFDTTEADVDAFVAAVRQEIDC

>39
IEMRSDTFTLPTEQMVSAMTQAVLGDDVYGEDPTANRLEELAAKSVGKPAACLMPSGTMA
NLAALLVHVPRGGKVLVGNESDIYLYEAGGASVCGGIVYEPIPTRPDGTLALDDLAAAFP
PDPDDPQFALPGLICVENTHNRMGGRVLSQAYLAELKRFATGHGIPVHMDGARIFNAAVA
TGVAAEQIAAHADSIQFCLSKGLSAPIGSILAGEADFIEKARRIRKMLGGGMRQAGVFAA
AGLVALTSMIDRLAEDHQRAAQLAAGLAEVDGIDVDPSSVTTNIVLFRVTANGLDDDRFL
RAVEQRGLVMGEFGHGRIRAVTHRGLSSADVSAAVAIVADVVREAS

>40
IEMRSDTFTLPTEEMLSAMGRATLGDDVYGEDPTVNHLEELAAKSVGKPAACLMPSGTMA
NLAALLVHVPRGGKVLVGDESDIYLYEAGGASVCGGMVYEPIPTRPDGTLALDDLAAAFP
PDPDDPQFALPGLICVENTHNRMGGRVLGQAYLAELKAFAAVHGVPVHMDGARIFNAAVA
SGLPAEEIAAHADSVQFCLSKGLSAPIGSILAGSAEFIGKARRVRKMLGGGMRQAGVFAA
AGTVALTDMVDRLAEDHRLAARFAAGLAEVDGIDVAPGSVTTNIVLFRVTAEGLDAARFL
EDARQRGLAMGEFGHGRIRAVTHRGLGPADVPAAVAIVADVVRAAR

>41
IEMRSDTFTLPTEEMLSAMGRATLGDDVYGEDPTVNHLEELAAKSVGKPAACLMPSGTMA
NLAALLVHVPRGGKVLVGDESDIYLYEAGGASVCGGMVYEPIPTRPDGTLALDDLAAAFP
PDPDDPQFALPGLICVENTHNRMGGRVLSQAYLAELKAFAAVHGVPVHMDGARIFNAAVA
SGFPAEEIAAHADSVQFCLSKGLSAPIGSILAGSAEFVGKARRVRKMLGGGMRQAGVFAA
AGTVALTDMVDRLAEDHRLAARFAAGLAEVDGIDVAPGSVTTNIVLFRVTAEGLDAARFL
EDARQRGLAMGEFGHGRIRAVTHRGLGPADVPAAVAIVADVVRAAR

>42
IEMRSDTFTLPTEQMMSAMSRAALGDDVYGEDPTVNGLEELAAKSPSGTMANLAALMVHV
PRGGKVLVGNESDIYLYEAGGASVCGGIMYEPIRTQPDGTLALDDLAAALPPDPEDSQFA
LPGLICVENTHNRMGGRPLSQEYLAELRRFATRHGLPVHMDGARVFNAAVATGVPAAEIA
AHADSLQFCLSKGLSAPIGSILAGEADFIEKARRVRKMLGGGMRQAGVFAAAGTVALTST
VDRLADDHRLAARFAAGLAEIDGIDVDPDAVTTNIILFRVTAPGLDDDRFLRAVQERGLA
MGEFGHGRIRAVTHRGLDAEDVSAAVAIVADVVREAR

>43
GMTNTLKTSYQKTPYKLGGNGPRNVGVLTEALQNIDDNLESDIYGNGAVIEDFETKIAKI
LGKQSAVFFPSGTXAQQIALRIWADRKENRRVAYHPLSHLEIHEQDGLKELQQITPLLLG
TANQLLTIDDIKSLREPVSSVLIELPQREIGGQLPAFEELEKISEYCHEQGISLHLDGAR
LWEITPFYQKSAEEICALFDSVYVSFYXGIGGIAGAILAGNDDFVQEAKIWKRRYGGDLI
SLYPYILSADYYFEKRIGKXAEYFEAAKGLAERFNSCSGVKTVPEVPVSNXFHVYFENSA
DEIGAILTKIQDETGVGISGYLQEKSADVCAFEVSVGDAFAEIPAKNLELVFRC

>44

GMNRLRTSFQQTTGQISGHGKRNVGVLKTAFAAVADEMASDQYGTGAIIEPFEQKFADVL
GMDDAVFFPSGTMAQQVALRIWSDETDNRTVAYHPLCHLEIHEQDGLKELHPIETILVGA
ADRLMTLDEIKALPDIACLLLELPQREIGGVAPAFSELETISRYCRERGIRLHLDGARLF
EMLPYYEKTAAEIAGLFDSIYISFYKGLGGIAGAILAGPAAFCQTARIWKRRYGGDLISL
YPYIVSADYYYELRKDRMGQYYEQAKQLAEQFNALPGVHTTPEVPVSNMFHLHFDGQAAD
ISPKLEQVQEETGLGFVGYLVDKDGYCSTEISVGDAYGELDQQTRDAGFAR

>45
INYLSDTITLPTEEMLKAIQNAELGDDVYGQDKTVNELEGYAANMLGMEAACFMPSGTMA
NLASILAHCPRGSTVLVGDESDIYIYEAGGASICGGIMYQPIKTQPDGRLLLSDLEAAFP
NDVKDPQFALPALICLENTHNRCGGKVLPLKYLKEIKEFAMRRNIPVHMDGARVFNAAVY
LGIEVKEIAQYVDSIQFCLSKGLSAPIGSMVVGSRKYIEEVRRIRKMLGGGMRQVGIIAA
PAMVALQTMTGRLQDDHIRAKRLAIGLSSIDGIEVDASEIQTNIVMFRITDPNYDWVKFL
KKTEEKGLVFSEMGYGRMRAVVHRHIKESDIEHTIKIIADLLNKSS

>46
IELRSDTFTLPTVEMLKAIIHAPLGDDVYQEDPTVNELETLVAKMMGKEAAILMPSGTMA
NLAALMAYCPRGSKVLVGNETDIYIYEAGGASVCGGIMYEPIPTQADGRLLIKDLARAFP
EDPTDPQFSLPSLICLENPHNRMGGRVLPLSYLKEVQMFSRQKEIPIHMDGARIFNAAIA
MDIPVKEIAQYADSVQFCLSKGLSAPIGSMIAGKKDFIQKVYRIRKMLGGGMRQAGIIAA
PALIALKQMNDRLTEDHVHARQLAEGLAQIEGIECDVDSVDTNIVFFQVIDKRYTWKTFI
EKAREHDLNIAELGHGRIRAVTHSGVNTQDINQALKIIKQIMQDNN

>47
SDCTPRFLASDNTSGICPEALDYLLRANASDDLAYGNDAWTQKAADRFRRFFDYDCDVFF
VFNGTAANSLTLASMGQSYHSVICHELAHIETDECGGPEFFSNGAKLLTCSGADGKLTPE
GIESLVTRRSDIHYPKPRTISLTQATEVGTVYTRDELMAIRAMADKHGLHVHMDGARFAN
ACASLEATPAELTWQAGVDALCFSGTKNGLPMGEAVVFFNHALAEDFGYRCKQAGQLSSK
MRFITAPWLGLLESGAWLRNAEHANGMARYLAAGFAALPGAELMFPAQANSVFVTLPVAV
LETLRQRGWTFYTFIGAGGARFVCAWNTTQALLDRLLDDARDALSA

>48
MRKSFASDNYSGIHPEVLESIIRANTGHVPSYGKDPYSEAAILKCKEHFGENIEVFFVFN
GTGANVLGLQAITRPYQSIICSDQAHLNVDECGAPERFTGCKLLTVPTADGKITPDQIEQ
HLIGFGDQHHSQPKVVSLTQSTEFGTVYRPEEIKAIADLAHEHGLLLHMDGSRLANAAAY
LGVPLKALTADVGVDVLSFGGTKNGLMIGEAVIFFDPDLARDFMYIRKQGMQLGSKLRFI
AAQFEALLSNDLWLQNARHSNRMASLLASRIEKIPGVRITQKVEANAVFAVIPRECIATL
SERYYFHVWSERESLVRWMTAFDTTEDEIDQFAEDIAQALSS

>49
MTHKMFASDNNSGIHPAILEAIAKANSGHAPAYGSDSYTADAIRKFKEHFSEDCEVALVF
NGTGANVTGLKAMVRSHQAIICTDGAHIHADEGGAAEGFIGGKLLTVPSADGKLTVDGIA
EQLHHIGNQQRSQPKAVSISQCTELGTVYTIEEIKEITSFAHANGLYVHMDGARLSNAAA
HLGCSFKEMTVDAGIDMLAFGGTKNGLMLGEAVVCFNKELASELTYIRKQGMQLGSKMRF
IAAQFERLLTDDLWHKNASQANRMAQLLAAGLQKFPDAALTQPVESNAVFISLPKEQIGQ
LQKTYAFALWNAAINEIRLVTSFDITEEDIKEFLSHVEKAYS

>50
MISIKISKNFASDNCSGVHPEILQAICEANQGHTLAYGDDVYTASALSKFREHFGKDIEV
FFVFNGTGANVLSLQAATDSFNAVICADTAHINCDECGAPEKFTGCKLLTIPSANGKITV

DQVEKFLHLAGNQHHSQPKAISITQSTELGTVYQPEEIQSIADFAHTHDMFLHVDGTRLA
NAAASLNLTLREISRDVGVDILSFGGTKNGMMYGEAVVIFNKNLHKNFSYIRKQGMQLAS
KMRFIAAQFEALLSNDLWLRNARHTNKLAKLLAEEVSKIPQIKITQPVQANAVFAIVPPQ
FIPALQQEYFFYVWNKETSEVRWMISFDTTEEDVMDFVRLIRQTIS

>51
MNGKRGFESDNCSSVHPRVMQALMDVNCGHVPGYGYDPVTAEADRIFNHLFDRETDVFFT
FNGTGTNCAALAHLVTPWQSILCADSAHINSAETGAPERIANAKLVPVPSIDGKISPRSL
EDAVGGWQSEHVPKPRVLSLTQVTDDGTVYTPGELKNLCKIAHDHNMIVHMDGARLANAV
AANQNDLTGTTWGAGVDVLSFGGTKNGLMFGEAVVFFDHKLAENFKYTRKSCGQLPSKMR
YIAAQFIEILKDGLWLEMAGHANRMAMMLYEKMSGLPVFRTPFVPQANELFAHVDERIKW
QLCDAFPFQHFGPEPGISRFVTSFDTTEEDLNALIACAEDLTHS

>52
MRSFASDNNARVHHEILEAIIRANDGDVISYGDDVYTQRAIEKFKEIFGKDIDVYFVYNG
TGANVLALTAITKPFNAIIASPMSHINVDECGAVEKSSGCKIITVPSSDGKIRIDDIKKL
LHAKGVEHHSQPKVISITQSTEVGTVYTVEEIKEITKFAHENDMYVHMDGARIANAAASL
NKSLREITKDAGVDVLSFGGTKNGMMFGEAVVFFNRELSKDFKFVRKQGTQLHSKMRYIS
AQFEALLSNDLWLKNAKHANEMAQYLEEKVKEIDEIKLTQRVEANAVFAIMPRKAIEKLQ
EKYFFYIWDEEKDEVRWMTSFDTKKEDIDLFVEEIKRAMRD

>53
VDLRSDTITLPTDEMRAAMAKAVVGDDVYGEDPTVNQLEQEAAALVGKEAALFVPSGTMG
NQIAVLTHTGRGDEVILDSEAHIYYYEVGGPAMLAGVQLQPVNGLLSDKGPELLKAALRS
EDIHFPHTSLVCLENTFNRGGGTILPPNIMSEIYAIAKQRGLQVHVDGARIFNAAVSLGI
DVREFTQYCDSVMFCLSKGLAAPVGSLLAGSKDFITRARKYRKALGGGMRQAGILAAAGL
VALKSIDRLAEDHANAKRLAAGLAELPGLHIDPARVQTNIVIVEVKGRLTAAELVNQLAE
RGVKCSTFGPNLIRLVTHKDVAAEDIEYALAAAKEILC

>54
VDFRSDTITKPTEEMRKAMYEAEVGDDVYKEDPTIKKLERVAADIVGKEAALFVPSGTMG
NQLAVLTHTERGQEVILEDWSHICRFEVGGIAFLSGVQAKTVKGTRGVMSLKDVNEAIVL
DNDIHHPQTGLICLENTHNMAGGVVVPIDVMNNIYNLAKENKLPVHLDGARVFNAAVYLG
CEVKEITKYTDSVMFCLSKGLSAPVGSILAGSEAFVEKARRYRKMFGGGMRQAGVIAAAG
LVALDKMIDRLKEDHDNIKLLASKLNQIDGIEIDNESVQTNILMINIESSKYSSKELVEE
MKKRGILTSVITDSIIRFVTHRHITKDNINYAVDKINEIMN

>55
IDLRSDTVTKPTEEMRKAMAQAEVGDDVYGEDPTINELERLAAETFGKEAALFVPSGTMG
NQVSIMAHTQRGDEVILEADSHIFWYEVGAMAVLSGVMPHPVPGKNGAMDPDDVRKAIRP
RNIHFPRTSLIAIENTHNRSGGRVVPLENIKEICTIAKEHGINVHIDGARIFNASIASGV
PVKEYAGYADSVMFCLSKGLCAPVGSVVVGDRDFIERARKARKMLGGGMRQAGVLAAAGI
IALTKMVDRLKEDHENARFLALKLKEIGYSVNPEDVKTNMVILRTDNLKVNAHGFIEALR
NSGVLANAVSDTEIRLVTHKDVSRNDIEEALNIFEKLFRKFS

>56
DTRSDTVTKPTPGMLQAMLNAAVGDDVYDEDPSIHKLEAFVAQLTGKEAALFCATGTMTN
QLAIRSQIGALESLICDIRAHIFRYECGGVAYHSQAHIIPLQPPNGGNITLEMIEAQVLT
FNVHHAVTKLIEIENTLGGAVFPYDEIVRISTYAKQHGIKMHLDGARIWNASIATGIPLK
QYCDHFDSVSLCLSKGLGAPIGSVLVGSRELIHRARHFRKLFGGGWRQAGLLAAGGQYAI

EHQWTRMADDHANAKQFAELITKAGHMTVTNKVETNMVFVLLNPELRKKGVSWNKIAARL
RSVHGIVVNEDGAHPAPVRFVFHLHITSEATAKLAAAINDEIAQ

>57
IDLRSDTVTKPSRPMLEAALSARTGDDVMGEDPTVLELEETVADLFRKEKGLYVPTGTMS
NLVAIMSHCNRRSSEIIIGANCHICLWEGGNTAGLGGVHTRQLLEDETTAQIHRDHISDA
FRSDDDDHFAKTELLCLENSHNMMGGVALPLSYVEDIGHLTTELGIKLHIDGARIFNASV
CHNVPVDNLCKPADSVSVCLSKGLGAPLGSVLVGDTDFIRLAKRARKRCGGGMRQAGVVA
AMGLYAIHNNISRLEADHRRAKRLANELQKNGFYLARNGAVDTNMFYFGLPDNSKVEWKD
YCKILQTKHGVGLTGGYSRGGRLFRAVTHLDISDKDIDRAVEAMVLAARLS
>58
IRSHSEQFASDNSSGICPEAWEYMLRANQGSAPAYGNDEWTQKAADYFRELFEIDCEVFF
AFNGTAANSLSLAALCQSYHSVICHETSHIETDECGAPEFASNGSKLLLAKGENGKLTPQ
SIEAIVTRRADIHYPKPKVISITQATELGTLYTPEELWEIKAVAQKYNLKIHMDGARFAN
AVAALNKSPAEISWKSGVDVLCFCGTKNGMALGEAILFFNRALAEDFDYRCKQAGQLASK
MRFISAPWLGLLETGAWLKNARHANQCAEYLENKLLNITGVEMMFPREANAVFAKLPEHV
IHSLKAKNWQFYTFIGVGGVRFMCSWNTTQSRIDELIGDIQAAIA

>59
MSMNAEQFASDNYSGICPEALEYMLQANLGSAPAYGNDEWTQKVSDYFRELFEINCEVFF
TFNGTAANSLSLAALCQSYHSVICHETAHIETDECGAPEFASNGSKLLLAPGENGKLTAQ
SIEAIVTKRQDIHYPKPKVISITQSTELGTVYSIAELLEIKEVAQKYKLKIHMDGARFAN
AVAAMNKTPAEITWKSGVDVLCFCGTKNGMALGEAILFFNKALAEDFDYRCKQAGQLASK
MRFISAPWLGLLETGAWLKNARHANQCAEYLENQLLQIDGVEIMFTREANAVFVKLPEQA
IQKLKDKNWQFYTFIGVGGVRFMCSWNTTKTRMDELVRDIQAAIA

>60
MDSNLEQFASDNSSGICPEALEYTIKANQGSVPAYGNDIWTQKATDSFRELFEIDCEIFF
VFNGTAANSLALASLCQSYHSVICHETAHIETDECGAPEFASNGSKLLLAKGQNGKLTPD
AIEEIINKRADIHYPKPKVISLTQATELGTIYSIDELIAIKSMAKKCNLKIHMDGARFAN
AVVSINKSPAELTWKSGVDVLCFCGTKNGMAMGEAIIFFNQALAEDFAYRCKQAGQLASK
MRFIAAPWLGLLETGAWLKNALHANQCAEYLENQLLQLEGVEIMFPREANSVFVKLPELA
IAQLREKNWQFYTFIGVGGVRFMCSWNTTQARMDELVGDIKEAIGR

>61
MLEQFASDNYSGICPEVMAYMNRANQGNAVAYGNDEWTQKAADYFRTLFETDCDVFFVFN
GTAANSLSLATLCQSYHSVICHEVAHIETDECGAPELASHGSKLLLGRGENGKLTPESIA
EIVNKRVDIHYPKPRAISITQPTEVGTLYSIEELMAIQDVAQQYGLKIHMDGARFANAIV
AMNQTPADLSWKCGVDVLCFCGTKNGMAVGEAIIFFNKSLAEDFAYRCKQAGQLASKMRF
IAAPFLGLLETGAWRKNAQHANECAEYLEERLSVIPEIEFMFPREVNSVFVKMPERVIQS
LREKSWHFYTFIGVGGVRFVCAWDTTKARIDELVNDIKDAIAI

>62
MLEQLEQFASDNYSGMCPEVLDALMSANQGSSPAYGNDQWTEKAANAFRELFEIDCEVFF
VFNGTAANSLALAALCQSYHSVIAHEVAHIETDECGAPEFFSNGSKLLLSSGENGKLTPE
GIAKIVTKRQDIHFPKPKVISLTQATEVGTVYQIEELQAIKAVADQYQLKIHMDGARFAN
AVVSLGKSPAEITWRSGVDVLCFSGTKNGMAMGESVIFFDRTLAEDFAYRCKQAGQLASK
MRFIAAPWLGLLNRDTWLKNARHANECAAYLASQLAAIPELTLMFPTEVNGVFVEMPEGL
IQALREKGWLFYNFIGVGGIRLMCSWATTRKRIDELVADIKSAIIS

>63
MNFASDNVTGISPEIMAALSAANAGVAMPYGQDEYTQRLKTQFNDLFETDVTVFPVATGS
AANALALSVMTPPFGAIYCHTESHINVDECGAPEFYTGGAKLVTLPGSQGKILATDLATV
LAQAGAGVVHHVQPAAVSLTQATEAGTVYTPDEIGALAEVAKSHRLYLHIDGARFANAVA
SLGCSPADLTWRAGVDVLCFGATKNGAMAAEAVVFFNQTLAETFGYRRKRSGHLFSKMRF
LSAQLLAYIEQDLWLNNANHANQMAQKLAQGLVELPGVELCYPVQANEIFIQLPKALISS
LLADGFYFYPWGGENSTTVRLVTAFNTSEADVMAFIEAARRYSSE

>64
MNFCSDNVAEVCPEIMAALIAANEGAMMPYGEDEYTQRLEAKFSSIFETPVTVFPVATGS
AANALSLSAIAPGYGAIYCHSESHINVDECGAPEFYTGGAKLVTLNGTDAQINPSELSKT
LEKAGAGVVHHVQPAAVSITQATEAGTVYLPEEIGEIAKITHAHNLYLHIDGARFANAVV
SLDCAPADITWRAGVDVLCFGATKNGAMAAEAVIFFNQDLAKTFAYRRKRSGNLFSKMRY
LSSQLEAYITDDLWLKNAFHANQMATRLAQGLVDLPLVKLCHPVEANEVFAEIPKSVVNG
LRGDGFQFYVWEEGAFPVIRLVTAFNTKKEDVDALLKSIQRHLRV

>65
LDFRSDTVTKPCLRMREAMFKAEVGDDYYRDDPTVQELERFAADLFGKEAALLTSSGTQS
NLIAIMSHCQRGEAFIVGHRSHAYLRELGGAAMIAGVQPQVVHNRADGKLSLEDIKASLF
PASILFAPVRLIAVENTIDGMVLPLDYLKDVADLATEHGLQVHTDGARIFNAAAAIGCPV
SNIACFSNTVSFCLSKGLGAPVGSVLVGSAQTIEKARRHRQMLGGGMRQAGILAAAGLFA
LQNNVSRLERDHARAKELADALSNLPGLTVETPQTNIVFCNVDASVQGKFSEFLKKNGIR
VSGTPDRQRWVTHLGIDDVALATTANLLTQMQGNCN

>66
IDLRSDTVTQPTPEMRQVMLAAPVGDDVLGDDPSVNELETYVAALLGKKAAVYMPSGTMT
NQVALRSHTQPGDEIVLEAEAHIYYYEGGGPAALSGVSCRLLSGEGGIFSAAAVEKSLRP
VDPHFPRTRLVCVENTHNRSGGRVFPLETIQEIAGVCQRNDLQLHMDGARFWNACVATGL
SPQEYAAPFDTVSVCFSKGLGAPVGSALVGSAQKIATARRFRKMFGGGMRQAGMIAAGAL
YAVQHQFERLAEDHYNAQRLAKKLKGIKGISVDPALVETNIVNFELTKFSASDVIKTLAK
EGVAVLATGPRKIRAVTNLMVSTEQIDQAVEAIARSVW

>67
IDLRSDTITQPTPAMRLAIANAAVGDDVLGDDPTVKELEADVAQLLGKEAAVYMPSGTMT
NQVAVRLHTQPGDEIVLESEAHIYYYESGAPAALSGVMCRLVSGRQGVFAADDLVNVLRS
GNPHYPPTTLVCIENTHNRGGGTIFPLEAIQAIAEVCQQRGLAIHMDGARLWNACEATGI
TPTDYAEPFDTVSVCFSKGLGAPVGSALAGSSETIDRARRFRKMFGGAMRQAGTIAAGAL
YALQHHRDRLCEDRENAQLLARQLVEVDGIEVDLSTVQTNIVVFRCLDVPAPLLAQALGD
RGVKLFARDRHSLRAVTSLMVTREHILQVKDKVADALTEVC

>68
IDLRSDTITQPTRGMREAIATAEVGDDVLGDDPTVQQLERFVAGLLGKEAAVFMPSGTMT
NQIALRTHTEPGDEVILESQAHIYYYEGGGPAALSGVMCRLINGDRGVFTAADVETVLRP
ADVHFPKTKLVCLENTHNRGGGKIYPLSEIDAIAQVCKQHGLKLHLDGARLWNACIATNT
PEDKYVKAFDSVSVCFSKGLGAPIGSALAGSHDFIERARRFRKMFGGGMRQAGMMAAGAI
YALKHHRAHLKEDHLNAQTLAKGLAEIPGIIIDPIAVETNIVVFHTKMLRAQVLVDRLKL
KGVYVLPVGPKSIRAVTNLMVTEEQILQVPDLVDLSLNES

>69
IDLRSDTITKPTPAMREAMAKAEVGDDVFGDDPTVNALEAYVAELLGKEEAVYMPSGTMT

NQVALRVHTEPGDEVILESEAHIYYYEGGAPAALSGVMCRLIKGNKGIFSATDVERVLRP
DNYHFPKTKLVCLENTHNRGGGRIFPLSEIEAIAHICQERNLKLHLDGARLWNACAATSI
SEADYAKPFDTVSVCFSKGLGAPVGSALVGSKELIKRARRFRKMFGGGMRQAGIIAAGAL
YGLKHHRERLLEDHVNAKILAKGLQQIDGIAIDPEDVQTNIVIFQTKAIPAETLAQNLQE
KGVALLAIGSHSLRAVTNLMVTQEQIQAVPELVEAAMRS

>70
DFRSDTVTWPTPAMVEAMATAEVGDDVYGEDPTVNELESLAASLLGKAAGLFVTSGTQGN
LIAALTHAQRGDEAILGEDAHTFCWEAGGIAVLGSITTRPLPTDSRGRMDVDRIKASLRV
DNPHLPHSRLILLENSSGGNNGAAIEPSYFESIGALAKQHDLAVHLDGARLFNAATALRL
DPTMITAHVDSVSVCLSKGLCAPVGSVLVGSEAFIHQARRHRKLLGGGMRQAGVLAAAGI
IALKTMSQRLQIDHDNAQTLAQGLATIPGIAIDPSVVETNMVFFDLAPEVAIAPEELIKA
LRTDYGLYMGGYGDRTLRAVTHYWIEQPQVERLVEAIRTILTGAA

>71
VDLRSDTVTRPTRAMRAAMFEAEVGDDVYGEDPTVNRLQERVAHLLGKEKGLFFPSGTMC
NQVAIATHTRPGQEIICDRDCHVFNYERAGASHLGGVQLHPLDGRHGVLSAQQVRSAVRP
IELHEPPTGLILLENTHNRGGGTVYPQDVIEQIAAVAGAAGIPMHLDGARLLNASVAGSH
SPATLAAPFDSVYFCLSKGLGCPAGSVLAGKADFIAQATRLRKAFGGGMRQVGFLAAAGL
YALDHHIERLADDHRRARQLAVHLKDLNAFDVELDAVHTNIVMVDVHADCTAAAVAADLT
AHGVRVSVFGPRRLRLVTHLDIDDAGVERTVAVFKRLYG

>72
MSTPRTTATAAKPKPYSFVNDYSVGMHPKILDLMARDNMTQHAGYGQDSHCAKAARLIGE
LLERPDADVHFISGGTQTNLIACSLALRPWEAVIATQLGHISTHETGAIEATGHKVVTAP
CPDGKLRVADIESALHENRSEHMVIPKLVYISNTTEVGTQYTKQELEDISASCKEHGLYL
FLDGARLASALSSPVNDLTLADIARLTDMFYIGATKAGGMFGEALIIILNDALKPNARHLI
KQRGALMAKGWLLGIQFEVLMKDNLFFELGAHSNKMAAILKAGLEACGIRLAWPSASNQL
FPILENTMIAELNNDFDMYTVEPLKDGTCIMRLCTSWATEEKECHRFVEVLKRLVASTA

>73
MVEAELPPQYTTASNDLRSDTFTTPTKEMIAAAMNASIGDAVYGEDVDTVKLEQHVAKLA
GKEAGIFCVSGTLSNQIALRTHLYQPPYSILCDYRAHVYTHEAAGLAILSQAMVVPVIPS
NGDYMTLEDIKSHYIPDDGDIHGAPTKVISLENTLHGIVYPLEELVRIKAWSMENGLKLH
CDGARIWNAAAETGVPLKQFGEIFDSISICLSKSMGAPMGSILVGNTKFIKKCNHFRKQQ
GGGVRQSGMMARMALCNVDDDWKSKLLYSHKLAHSLADFCEKNGIPLESPADTNFVFIDL
RKARMDPDVLVKKGLKYNVKLMGGRVSFHYQVTEQTLEKVKLAILETFEYAKQHPFDQNG
PTQIYRSESTEIDIEGNAIKEIKTYKY

>74
MTDMELPPAYITASNDLRSDTFTTPTEAMMKAALTASVGDAVYGEDIDTVKLEQKVAKLA
GFEAGLFCVSGTLSNQIALRTHLYQPPYSVLCDYRAHVYTHEAAGLAILSQAMVVPVIPS
NGNYLTLQDIKRHYVPDDGDIHAAPTKVISLENTLHGIVYPLEELIRIKGWCMENGLKLH
CDGARIWNAAVQANIPLKQYGEIFDSISICLSKSIGAPMGSVLVGDVKFIKKCNHFRKQQ
GGGVRQSGIMARMAMVAIEDNWKERLHRSHSLAHNLAAFCKKQGIPLESPADTNFVFVDL
RKARMDPDVLVKKGLKYNVKLMGGRVSFHYQIEDDVLERVKLALSEAFEYAKQHPCDQDG
PTQVYRSASTEIDVHGNAIQEIKTYKY

>75
DFRSDVVTVPVEEMMTSILEASVNDDIYDPEGDPSVKALEARLVELTGMEAALWAVSGTQ

GNQICLRTHLTQPPHSVLLDHRAHVHCWESGALPVISQASVTTVHAKNGVHLTLEDVKKN
IIADGNIHFPPTRVVSLENTLSGTILPLADAQAISDYVRSFPVPEGQKPIAMHLDGARVF
DGVIGEGVDLKAYAACFDSISICLAKGIGAPMGSVILGKKSFIERAKWFRKMLGGGTRQP
GMMAAAALSALEYSIPRFPSVHAMTKDAAARLEAVGYMFTLPVQTNMILLDLEAVEIPPA
AFVEYCAKEDISVFPMARLVFHHQTSEGAVDKLVTALTRLMEDKRNGVTLNDGEAHGGYS

>76
MIESSFQDDMYDATGDESVNRLQQRLVELTGKEAAMWALSGTMGNQICLRTHLTQPPHTV
LLDYRAHVHCWETGAMPVMSQAAVTQVHPKNGLHLTLEDVKKHIIADGNIHFPPTRVVSL
ENSLSGTILPLKDAKEISDFVRNFPVEEGEKPIAMHLDGARLFDAVAAEGVSLKEYCACF
DSISICLAKGLGAPMGSIIVGSKKFIHRARLFRKMFGGGTRQPGMMAAAALCALEYTMPL
LPKVHALTKRTADSLREAGYKITLPVQTNMIVLDLEEVGIPPAAFVEYGKRAGVTVFPTG
RLVFHHQISAAAASRLVDALTSLMQDKKAGRQLENHKVTGGYM

>77
MTADEEETFTTFNEFRSDTFTVPTRAMVEDGFMNATFGDSVYQEDETTLRLESKMCEITG
KPAALFCVSGTMSNQIGLRANLVQPPYSILCDYRAHVFLHEAGGLATLSQAMVHPVRPSN
GNYLTFEDVLGNVTYDDDGDIHAAPTKVISLENTLHGIIIPIEEIRKISEFCRENDIRLH
LDGARLWNASVATGISIKEYCSYFDSVSLCLSKSLGAPIGSVLVGDEKFIRKANHFKKQS
GGGIRQAGIMSAMAIHAIDYNLSKLELSHNYAKQIGDFCQEHGIKLESPVDTSLVFLDLK
ANKMDPNRLVELGRTKYNVKLMGQRIACHFQLSQESVDNVKKCILECLEYHQKHPHKDDG
RNNKKMYSLDAIKK

>78
MTEFELPPKYITAANDLRSDTFTTPTAEMMEAALEASIGDAVYGEDVDTVRLEQTVARMA
GKEAGLFCVSGTLSNQIAIRTHLMQPPYSILCDYRAHVYTHEAAGLAILSQAMVVPVVPS
NGDYLTLEDIKSHYVPDDGDIHGAPTRLISLENTLHGIVYPLEELVRIKAWCMENGLKLH
CDGARIWNAAAQSGVPLKQYGEIFDSISICLSKSMGAPIGSVLVGNLKFVKKATHFRKQQ
GGGIRQSGMMARMALVNINNDWKSQLLYSHSLAHELAEYCEAKGIPLESPADTNFVFINL
KAARMDPDVLVKKGLKYNVKLMGGRVSFHYQVTRDTLEKVKLAISEAFDYAKEHPFDCNG
PTQIYRSESTEVDVDGNAIREIKTYKY

>79
MNQDMELPEAYTSASNDFRSDTFTTPTREMIEAALTATIGDAVYQEDIDTLKLEQHVAKL
AGMEAGMFCVSGTLSNQIALRTHLTQPPYSILCDYRAHVYTHEAAGLAILSQAMVTPVIP
SNGNYLTLEDIKKHYIPDDGDIHGAPTKVISLENTLHGIIHPLEELVRIKAWCMENDLRL
HCDGARIWNASAESGVPLKQYGELFDSISICLSKSMGAPMGSILVGSHKFIKKANHFRKQ
QGGGVRQSGMMCKMAMVAIQGDWKGKMRRSHRMAHELARFCAEHGIPLESPADTNFVFLD
LQKSKMNPDVLVKKSLKYGCKLMGGRVSFHYQISEESLEKIKQAILEAFEYSKKNPYDEN
GPTKIYRSESADAVGEIKTYKY

>80
MTEFELPPKYITAANDLRSDTFTTPTAEMMEAALEASIGDAVYGEDVDTVRLEQTVARMA
GKEAGLFCVSGTLSNQIAIRTHLMQPPYSILCDYRAHVYTHEAAGLAILSQAMVVPVVPS
NGDYLTLEDIKSHYVPDDGDIHGAPTRLISLENTLHGIVYPLEELVRIKAWCMENGLKLH
CDGARIWNAAAQSGVPLKQYGEIFDSISICLSKSMGAPIGSVLVGNLKFVKKATHFRKQQ
GGGIRQSGMMARIALVNINNDWKSQLLYSHSLAHELAEYCEAKGIPLESPADTNFVFINL
KAARMDPDVLVKKGLKYNVKLMGGRVSFHYQVTRDTLEKVKLAISEAFDYAKEHPFDCNG
PTQIYRSESTEVDVDGNAIREIKTYKY

>81
DFRSDTVTRPTEQMLAAIAATTLQDDDFRQDPTTLGLEAWMAELTGKAAGLFVVSGTMGN
QLGVRAHLQSPPHSVLCDARSHLVTHEAGGVASLSGAMVSCVTPVNGRYMTQADLEAHVN
RGTLITDCPTRLVVLEIPLGGVILPLDKCRRISEWARAQGIALHLDGARLWEAVAAGAGS
LRDYCACFDSVSLCFSKGLGAPIGSVLVGSETLRERARWIRKSIGGGMRQAGVVCAAARV
AVEATFLGGLLKRSHARARDIATFWEIHGGRLTYPTETNMVWLDLEAVGWTPERLIRRGA
ELGLRFMGARLVVHYQIGDEAIGRLQDLMLEILVS

>82
DFRTDTITMPTDEMFELMRDASRGDDVYGEDQATNDLQDKIAKMAGKEAGLFCVSGTMTN
QLAIRTHLTQPPHSIILDARSHVHLYEAGGVAMHSQASSHAVPPRNGHHLTLEEILDNAV
FGEDIHSAPTKLVSLENTLSGMVFPQEEIVRISDCMRSNGIIMHCDGARMWEAAVKTGLS
LEELCRPFDTVSLCLSKGLGAPVGSVLVGPRKFIEKVKWFRKAFGGGIRQCGSLAVAADY
CVDNHFLKLKGTHELASYLAKSLADLGAQLLLPVETNMLWLDPSSLGFSIADLAARAKTR
GITLGSNRIVVHHQITREACEDLVALVAELKDE

>83
VNFLSDTVTCPTVGMRQVIAAAEVGDDVFGADPSVKRLEKVAAERLGKPAALYVPSGTMS
NLIAIGTHCRRGDEVICGDKAHIFLYEGGGASAYMGVSLHTVPNQPDGTLDIKDIHNAVR
DDDPHYPRTRLVEIENTQNTCGGRVLPLSYIREVEQFCQERDLRLHVDGARLANASVASG
IPMDELARGADSVSLCLSKGLGAPVGSILAGSEEFIHHARRLRKSLGGGMRQAGIIASAG
LYALENQFDRLEEDHVNAQALAHGISSIPGVEIDPSTVDTNIVFFTLTKDAKLDATTLVQ
KLGSEKGVLVGAYADGNRVRAVTNLHISAEDVEYTISSVRALLN

>84
VNFLSDTVTRPSAAMRQVIAAAEVGDDMFGADPSVKRLEKVAAEQLGKAAALYVPSGTMS
NLIAIGTHCRRGDEVICGDKSHIFLYEGAGASAYMGVSLHTVPNQADGTLDIKDIHNAVR
DDDPHYPRTKLVEIENTQNTCGGRVLPLSYIREVEQLCHERDLRLHVDGARLANASVASG
TPMDELVRGADSVSLCLSKGLGAPVGSVLAGSEEFIHQAKRFRKSLGGGMRQAGIIASAG
LYALENQFDRLVEDHVNAQALAHGISSIPGVETNLDTVDTNIVFFALTPDAKLNATTLVQ
KLESEKGVLVGAYADGNRVRAVTNLHISSQDVEYTVASIRALLS

>85
IDLRSDTVTRPDDRMREASANAEVGDDVYREDPTVNELEERAAEIVGTEAALYVPTGTMG
NQIAVRTHTERGQEVLCERESHVVKWELGGMAQLSGLQVRTVEGDDRGVVSPERVRESYV
AEDLHRPGTGLLTLENTHNSKGGAAIEVEKIRETAETAHGLDVPVHLDGARVFNAAAALG
VEAREIVAPVDSVMFCLSKGLGAPVGSMLAGSQEFVDRARRNRKLLGGGMRQAGIIAAPG
ALALENRHRLTDDHENAHRLAAGLDGIEGLSVLEPETNIVLVDCEGTGHTAEEFLAAVES
TGVLGVAFGEYVVRFCTHWDVDDGDVEEAISAVDRAL

>86
IDLRSDTVTTPGEAMRQSAADADVGDDVYGEDPTVNELEARAADAVGMEAALYVPSGTMG
NQIAARVHTERGQEVLVEQESHVYKYELGGFAQHSQLQVRTYAGGANGCPTPEQVREGYV
EEELHRPGTGLLCLENTHNVKGGVPVPRDELAAAADAASNLGLPVHVDGARVFNAATALD
VDAADLLAPADSVMFCLSKGLGAPVGSMLAGSEAFVERARRTRKLMGGGMRQAGVIAAPG
LLALENRERLDVDHENARRLAAELDDLEGLSVPVPETNIVLVDTTETGLSAAEFLENCEA
EGVLGSEFGEYTVRFCTHLDVDAADVDECVAAVERAR

>87
LDFRSDTVTNPTPEMREAAAKAPLGDDVYREDPSINRLEKISAEILGKEAGLFVTSGTMG

NAVAILAHTQRGDEIILEEKSHIFMNEVGGLAVMGSLMARTISGDLGWMKPEDIRAAVRA
ENIHYPKTSLVCIENTHNSAGGIALTVDQMKMDWDVSKENELGVHLDGARVFNAAIALDV
NVKELTQYADTVQICLSKGLSAPVGSVVVGSYDLIEKARKYRKMLGGGMRQAGIIAAPGI
IAITKMVDRLAEDHENAKVLSEGLRNLGIKIVNPVQTNMVYIDLSSIGWTGKEFTEACAK
IGWKIRGASPIVRLCTHYGIEREDIDTFLEGMAKLVP

>88
IDLRSDTVTKPDDAMREAARTADVGDDVYGEDPTVNELEARVAEAVGKDAALYVPSGTMG
NQIAARVHTERGQEVLADRESHVVAYELGGFAQHANLQVRTLETDRGVPTPEQIADEYVT
EDLHRPGTGLLCLENTHNARGGLAIDPERIAAAAEAARERHVPVHLDGARVFNAAMALDV
PVTEITDPVDSVSVCLSKGLGAPVGSVLAGDEAFVERARRVRKLLGGGMRQAGIIAGPGL
EALENVSELESDHENAQALADGLAAVPGFDVREPETNIVLADISGTGLETAAVVERLRDR
DVLASEFGPATVRFCTHRDVSRADIGRALEKITETFE

>89
IDLRSDTVTLPSDEMREAARDAAVGDDVYGDDPTVNELEARAAELVGKEAALFVPSGTMG
NQIAARVHADPGQEALVDEKAHVYKWEVGGFAQLSGLQVRAYDAGERAAPTPAQVRDHAR
EESLHVAGTGVLCLENTHNARGGVAVPKADIDAAADAARDLGIPVHLDGARLFNACVALD
EDPTAMVERVDTVMCCLSKGLGAPVGSILAGPEAFIEEAVRVRKQFGGGMRQAGLIAAPG
LVALDNVDRLADDHANATVLAEGLDAVSGLSVPTPDTNIVVVDSEGAGLTAEEFVELCDD
VGVLGGTFGRYHTRFTTNLNVSRADVERAVDLVADAVESR

>90
IDLRSDTVTLPSDEMREAARDAAVGDDVYGDDPTVNELEARAAELVGKEAALFVPSGTMG
NQIAARVHADPGQEALVDEKAHVYEWEVGGFAQLSGLQVRAYDAGERAAPTPAQVRDHAR
EESLHVAGTGVLCLENTHNARGGVAVPKADIDAAADAARDLGIPVHLDGARLFNACVALD
EDPTAMVERVDTVMCCLSKGLGAPVGSMLAGPEAFIEEAVRVRKQFGGGMRQAGLIAAPG
LVALDNVDRLADDHANATVLAEGLDAVSGLSVPTPDTNIVVVDSEGAGLTAEEFVELCDD
VGVLGGTFGQYHTRFTTNLNVSRADVERAVDLVADAVESR

>91
IDLRSDTVTTPSNAMRDAARDADVGDDVYGEDPTVNELEAAVADRLGFEAAVFVPTGTMG
NQIAIRTHTEPGQEILVDREAHVFNWEVGGLALHSGLQTRPFEETVRRDGRERGVPTPEA
IESGYVEESVHRAGTGLVSLENTHNGRGGLAIAPERIDAAAEAAHDLGVPVHLDGARLFN
AAVAHDVPAERFTRQVDSAMVCLSKGLGAPIGSMLAGSAEFVEAARRNRKLLGGGMRQAG
IVAAPGLLALDSVDRLADDHRNADRLAVGLADLPGLDVVAPETNIVLVDVSDAAQDAAAF
KQACADAGVACTTMDETTARFCTHRNVDKADVDEALSLVADVVQD

>92
IDLRSDTVTTPSEAMREAARDAEVGDDVYRDDPTVNELERRAAEAVGTEAALYVPSGTMA
NQIAVHVHTEPGQELILERESHIYRWELAGAAKLSGTQTRTLDAGERCVPTPEAVREGLV
DEDLHRPGTGLLALENTHNYRGGTAIPVDRISAAAEVARDAGVPVHLDGARVFNAAVALG
VDASEIVDPVDSVTFCLSKGLGAPVGSILAGDEAFVEAARRVRKLFGGGMRQAGMIAAPG
LLALENVDRLADDHANAERLAAGLDALDGVRAPEPDTNIVVAHTEDAGIAAADLVTACKD
AGVGCVEFDDYATRFTTHLDVDGDDVDAAIDRIGDVVAELA

>93
MSNNKGFASDNHSGIHPDILKAIISANVGHANAYGNDDYTKRAVKKFEEYFGSNIEVYFV
YNGTAANILGLKTLTDSFNSIICAETAHLNVHECCGPENFIGCKLITIPTVDGKLTINQI
KPHIFGFEDPHRAQPKIISITQPTELGTVYTPEEIRKLSNFAHRNRMLIHIDGARLCNAA

AYLDVEMKDITGNVGVDILSFGGTKNGMMFGEAVIFFNKQSSKNFVFIRKQGMQLPSKMR
FISSQFETLLSNDLWLKNAKHSNRMAQLLYKEIRDVPQIKITQKVETNAIFAIVPKKYIH
LLQKEYFFHVNNERISEVRWMCSFDTTKKDIMNFSEIIKKTVV

>94
VDLRSDTVTRPTDAMREAMCNAEVDDDVLGYDPTARRLEEEMAKMMGKEAALFVPSGTMG
NLISVMVHCDVRGSEVILGDNCHIHVYENGGISTIGGVHPKTVKNEEDGTMDLEAIEAAI
RDPKGSTFYPSTRLICLENTHANSGGRCLSVEYTEKVGEIAKRHGVKLHIDGARLFNASI
ALGVPVHKLVKAADSVQVCLSKGLGAPVGSVIVGSQSFIEKAKTVRKTLGGGMRQIGVLC
AAALVALQENLPKLQHDHKKAKLLAEGLNQMKGIRVNVAAVETNMIFMDMEDGSRFTAEK
LRKNLEENGILLIRGNSSRIRIVIHHQITTSDVHYTLSCFQQAMLTMQ

>95
VDLRSDTVTKPTESMRSAMANAEVDDDVLGNDPTALRLEKEVAEIAGKEAAMFVPSGTMG
NLISVLVHCDERGSEVILGDDSHIHIYENGGVSSLGGVHPRTVKNEEDGTMEIGAIEAAV
RSPKGDLHHPVTKLICLENTQANCGGRCLPIEYIDKVGELAKKHGLKLHIDGARIFNASV
ALGVPVKRIVQAADSVSICLSKGIGAPVGSVIVGSKKFITKARWLRKTLGGGMRQIGLLC
AAALVALHENVAKLEDDHKKARVLAEGLNRIERLRVNVAAVETNIIYVDIPEDPKFGAEE
ACKSLEDVGVLVIPQATFRIRIVLHHQISDVDVEYVLSCFEKIFHS

>96
VDLRSDTVTRPGPAMRRAMAEAVVGDDDYGEDPTVHELQEKAAELLGVERTLFVPTNTMA
NLISVMGHCRRRGSQVLLGQECHLHVYEQGGVAQIAGVHSHPLPDLPYGTLDLNELERAL
TRGSGSPYHPVCELVYLENTHSSAGGRVLPVDYLRQVCLLAHAHGARVHMDGARLMNAAV
ALRIPPARLVEHCDSVSFCFSKGLGAPVGALVGGSKDFIGEAWRLRKALGGGMRQAGVLA
AAALVGLAEAEEVLPRDHENAQRFAKGLQDLASPICSVDPATVETNMVLVQVAGLPPSEL
CQRLQAVSAEEVAQTGRAVRVLLFPWTEQSVRAVWHRDVSAQDTELALKKWEFVLRQLR

>97
MNGETSRPPALGFSSDNIAGASPEVAQALVKHSSGQAGPYGTDELTAQVKRKFCEIFERD
VEVFLVPTGTAANALCLSAMTPPWGNIYCHPASHINNDECGAPEFFSNGAKLMTVDGPAA
KLDIVRLRERTREKVGDVHTTQPACVSITQATEVGSIYTLDEIEAIGDVCKSSSLGLHMD
GSRFANALVSLGCSPAEMTWKAGVDALSFGATKNGVLAAEAIVLFNTSLATEMSYRRKRA
GHLSSKMRFLSAQIDAYLTDDLWLRNARKANAAAQRLAQGLEGLGGVEVLGGTEANILFC
RLDSAMIDALLKAGFGFYHDRWGPNVVRFVTSFATTAEDVDHLLNQVRLAADR

>98
LAAIDELVAEEEAEDARVLHLTANETVLSPRARAVLASPLTSRYLLEHLDMRGPSPARLG
NLLLRGLDRIGTIEESATEVCRRLFGARYAEFRCLSGLHAMQTTFAALSRPGDTVMRVAT
KDGGHFLTELICRSFGRRSCTYVFDDTMTIDLERTREVVEKERPSLLFVDAMNYLFPFPI
AELKAIAGDVPLVFDASHTLGLIAGGRFQDPLREGADLLQANTHKTFFGPQKGIILGNDR
SLMEELGYTLSTGMVSSQHTASTVALLIALHEMWYDGREYAAQVIDNARRLAGALRDRGV
PVVAEERGFTANHMFFVDTRPLGSGPAVIQRLVRAGVSANRAVAFNHLDTIRFGVQEITR
RGYDHDDLDEAADLVAAVLLERQEPERIR

>99
MGASGKTSLGVDPLMLGRAIIDADRRAAHALNLVPSENRISPLASLPLGSDFYNRYFFNT
DGDPLFWEFRGGEEIAHIEALGIDALRRMASARYCNVRPISGMSAMIITVAALSRPGSTV
VSVDQNSGGHYATSALLGRFGRESRLLGGGGGRVDESRLADLLAPGDVDLVYVDVQNCVR
TPDFRAMSAVVKDVSPGTRLYVDASHYLGLVFGGHVVNPLTCGADAFGGSTHKSFPGPHK

GVIFTNSDDVDEKLRAAQFDLLSSHHFAETLALALAALEVEQHIGEYARATNDNARRLAR
ALADAGFRVHGDSSAGYTDTHQVWVELDNTADAYALSNRLADVGIRVNLHSTLPGVPGVH
LRLGSNEVTFEGAGPRAIERLADALVTARERALEPRTVSEIRRRFGAPFYTDSEK

>100
VDRLLKSWLSLTEESREALNLVPSENRMSPLAMLPLSSDFYNRYFFNDRLDSGFWQFRGG
QSASHFEVDVALPSLRRLTASEYVNLRPISGLNTMLIAVAGLGGVPGSAVVSIGHDWGGH
YATSALVERLGLRSVTVRVERGKVDASELERVLRQDAPTLVYLDLQNSLDPLDVPPVAAA
IRTHSPGTLLHVDASHVLGLILGGAIPNPLDAGADSVGGSTHKTFPGPHKGILFTRDAEI
ADRLRRAQFNLLSSHHFAETISLGLAALEFENFGAAYAEQVLRNATAFGRALRHRGFDVI
GDPERPTGTHQVWVRIGDAPRTDHVAENLYLAGIRVNVQTDLPGLPGAMFRLGVSEVTFE
GADEDAMGLLADAFVHAADGRPGRAAGIRADIRAGMTRPYFYTGAE

>101
MTDTNELRKVLHRFRSQQEKAEFSVNLVPSENKLSPLAQLPLRSDYYNRYFFNDALDPGF
WQFRGGQDVAEMETELTVDHLSRLARAPHVNERPISGLSAMMIAMAGLGGKPGGTVVSID
AASGGHYATASMARRLGFESATVPVVRGQVDEQRLEQVLCRHEPELIYLDLQNSRHELEV
SRVADLVGRHSRQTLLHVDCSHTMGLVLGGALGNPLDAGANTMGGSTHKTFPGPHKGVLF
TRTPELHQRMREAQFTMLSSHHFAETLALGLASAEFSHFGPAYAEQVIGNAQLFSKLLAS
EGFDVATDEDGHTTSTHQIWVKIGDAEQTDRISQSLYDHGIRVNVQVDLPGMPGPVLRLG
ISELTFVGGREAAVHALAREFSNARAGVRRDGSGSRRVREQCGSPFHFVDYP

>102
MTDIRELRKVVDRFRAQERKAAASINLVPSENKLSPLAQMPLSTDYYNRYFFNDELDPGF
WQFRGGQEVAKIQTELARGHLSRLARAPYVNERPISGLSAMMMAMAGLGGPPGGTVVSID
AASGGHYATADMARRLGFESATVPVVRGRVDEQWFGQVLREHVPELVYLDLQNSRHELEV
SRVAELIEAHSPHTILHVDCSHTMGLILGGALSNPLDAGAHTMGGSTHKSFPGPHKGVLF
TRSPELHQRLKHAQFTMLSSHHFAETLALGLAAAEFRHFGHAYAEQVVANARLLGKLLAA
DGFDVTADENGHATSTHQLWVRIGDAEQTDRFSKYLYDHGIRVNVQVDLPGLPGPVLRLG
VNELTFLGGHEAAVHALAEEFSHARDGVRRDGEGSQRVREQYGPPFYFVEFS

>103
MTGIKELRDVVDRFRAEERKAATAVNLVPSENRLSPLAQLPLSTDYYNRYFFNDALDPGF
WQFRGGQEVAEIQTELARGHLSRLSRAPHVNERPISGLSAMMMALAGLGGKPGGTVVSVG
AESGGHYATAGMARRLGFESATVPVAHGQVDEQRLGQLLRERTPQLLYLDLQNSRHELEV
SRVAELIKEYSPSTLLHVDCSHTMGLILGSALGNPLDAGADTMGGSTHKTFPGPHKGVLF
TRSPELHQRLKDAQFTMLSSHHFAETLSLGLAAAEFHHFGQAYAEQVIANARLFSKLLAA
DGFDVAADENGHATSTHQVWVKIGDAERTDRISQALYEHGIRVNVQVDLPGLPGPALRLG
VNELTFTGGREAAVHALAEEFGNARAGVRRDGDGARRVCEQSGPPFYFAEFS

>104
MFSRDLTIAKYDADLFAAMEQEAVRQEEHIELIASENYTSPAVMEAQGSVLTNKYAEGYP
GKRYYGGCEYVDVVEQLAIDRAKELFGADYANVQPHAGSQANSAVYLALLQGGDTILGMS
LAHGGHLTHGASVSSSGKLYNAVQYGIDANGLIDYDEVERLAVEHKPKMIVAGFSAYSQI
LDFPRFREIADKVGAYLFVDMAHVAGLVAAGVYPNPVPYADVVTTTTHKTLRGPRGGLIL
ARANAEIEKKLNSAVFPGAQGGPLEHVIAAKAICFKEALQPEFKTYQQQVVKNAKAMAGV
FIERGFDVVSGGTENHLFLLSLIKQDISGKDADAALGKAFITVNKNSVPNDPRSPFVTSG
LRFGTPAVTTRGFKEAECKELAGWICDILADLNNEAVIDAVREKVKAICKQLPVYGA

>105

MFSKQDQIQGYDDALLAAMNAEEQRQEDHIELIASENYTSKRVMQAQGSGLTNKYAEGYP
GKRYYGGCEHVDKVEALAIERAKQLFGADYANVQPHSGSSANSAVYLALINPGDTILGMS
LAHGGHLTHGAKVSSSGKLYNAVQYGINTDTGLIDYDEVERLAVEHQPKMVVAGFSAYSK
TLDFPRFRAIADKVGALLFVDMAHVAGLVAAGLYPNPLPYADVVTTTTHKTLRGPRGGLI
LAKANEAIEKKLNAAVFPGAQGGPLMHVIAGKAVCFKEAQEPGFKVYQQQVIDNAQAMAE
VFIKRGYDVVSGGTDNHLFLVSLIRQGLTGKDADAALGRAHITVNKNAVPNDPQSPFVTS
GLRIGTPAVTTRGFKVAQCIELAGWICDILDNLGDADVEANVAKHVSALCADFPVYR

>106
MFSKHDQIRGYDDELLAAMDAEEARQEDHLELIASENYTSKRVMQAQGSGLTNKYAEGYP
GKRYYGGCEHVDKVERLAIDRARQLFGADYANVQPHSGSSANAAVYLALLNAGDTILGMS
LAHGGHLTHGAKVSSSGKLYNAVQYGLDTATGLIDYDEVERLAVEHKPKMIVAGFSAYSK
TLDFPRFRAIADKVGALLFVDMAHVAGLVAAGLYPNPIPFADVVTTTTHKTLRGPRGGLI
LARANEEIEKKLNSAVFPGAQGGPLMHVIAAKAVCFKEALEPGFKDYQAQVIRNAKAMAE
VFIGRGYDVVSGGTDNHLMLISLVRQGLTGKEADAALGRVGITVNKNAVPNDPQSPFVTS
GIRIGTPAITTRGLQEAQSRELAGWICDILDHLGDADVEAKVATQVAGLCADFPVYR

>107
MFSKHDQLQGYDDELLAAMDAEDRRQEDHIELIASENYASKRVMQAQGGGLTNKYAEGYP
GKRYYGGCEHVDKVERLAIDRARQLFGADYANVQPHSGSSANAAVYLALLNAGDTILGMS
LAHGGHLTHGAKVSSSGKLYNAVQYGLDTATGLIDYDEVERLAVEHKPKMIVAGFSAYSK
TLDFPRFRAIADKVGALLFVDMAHVAGLVAAGLYPNPIPFADVVTTTTHKTLRGPRGGLI
LARANEEIEKKLNSAVFPGAQGGPLMHVIAAKAVCFKEALEPGFKDYQAQVIRNAKAMAE
VFIGRGYDVVSGGTDNHLMLISLVKQGLTGKAADAALGAAHITVNKNAVPNDPQSPFVTS
GIRIGTPAVTTRGFREGECRELAGWICDILDDIDNPEVGERVRGQVGEFCRHFPVYAD

>108
MFSRDLTLARYDAELFAAMEQEAQRQEEHIELIASENYTSPAVMEAQGSVLTNKYAEGYP
HKRYYGGCEYVDIVEQLAIDRAKQLFGADYANVQPHAGSQANAAVYLALLSAGDTILGMS
LAHGGHLTHGASVSSSGKLYNAVQYGIDANGLIDYDEVERLAVEHKPKMIVAGFSAYSQV
LDFARFRAIADKVGAYLFVDMAHVAGLVAAGVYPNPVPFADVVTTTTHKTLRGPRGGLIL
ARANEEIEKKLNSAVFPSAQGGPLEHVIAAKAVCFKEALQPEFKTYQQQVLKNAQSMAQV
FLDRGFDVVSGGTQNHLFLLSLIKQDITGKDADAALGRAFITVNKNSVPNDPRSPFVTSG
LRIGTPAVTTRGFKEAECRELAGWICDILENMGDESVVDGVREKVKAICAKFPVYGN

>109
MFSKKDQIQGYDDALLAAMNAEEQRQEDHIELIASENYTSKRVMEAQGSGLTNKYAEGYP
GKRYYGGCEHVDKVEALAIERAKQLFGADYANVQPHSGSSANSAVYLALLNAGDTILGMS
LAHGGHLTHGAKVSSSGKLYNAVQYGINTDTGLIDYDEVERLAVEHKPKMVVAGFSAYSK
TLDFPRFRAIADKVGALLFVDMAHVAGLVAAGLYPNPLPYADVVTTTTHKTLRGPRGGLI
LAKSNEEIEKKLNAAVFPGAQGGPLMHVIAGKAVCFKEAQEPGFKVYQQQVIDNAQAMAS
VFIKRGYDVVSGGTDNHLFLVSLIRQGLTGKEADAALGRAHITVNKNAVPNDPQSPFVTS
GLRIGTPAVTTRGFKVPQCIELAGWICDILDNLGDADVEANVAKHVSALCADFPVYR

>110
MFSRDLTIAKYDADLFAAMEQEAVRQEEHIELIASENYTSPAVMEAQGSVLTNKYAEGYP
GKRYYGGCEYVDVVEQLAIDRAKELFGADYANVQPHAGSQANSAVYLALLQGGDTILGMS
LAHGGHLTHGASVSSSGKLYNAVQYGIDANGLIDYDEVERLAVEHKPKMIVAGFSAYSQI
LDFPRFRAIADKVGAYLFVDMAHVAGLVAAGVYPNPVPYADVVTTTTHKTLRGPRGGLIL
ARANAEIEKKLNSAVFPGAQGGPLEHVIAAKAICFKEALQPEFKTYQQQVVKNAQTMASV

FIERGFDVVSGGTENHLFLLSLIKQDISGKDADAALGKAFITVNKNSVPNDPRSPFVTSG
LRFGTPAVTTRGFKEAECKELAGWICDILADLNNEAVIDAVREKVKAICKKLPVYGA

>111
MFSRDLTIAKYDAELFEAMQQEALRQEEHIELIASENYTSPAVMEAQGSVLTNKYAEGYP
GKRYYGGCEYVDVVEQLAIDRAKELFGADYANVQPHAGSQANAAVYLALLSAGDTILGMS
LAHGGHLTHGASVSSSGKLYNAIQYGIDGNGLIDYDEVERLAVEHKPKMIVAGFSAYSQV
LDFARFRAIADKVGAYLFVDMAHVAGLVAAGVYPNPVPFADVVTTTTHKTLRGPRGGLIL
ARANADIEKKLNSAVFPGAQGGPLEHVIAAKAICFKEALQPEFKAYQQQVVKNAQAMASV
FIERGFDVVSGGTQNHLFLLSLIKQEISGKDADAALGKAFITVNKNSVPNDPRSPFVTSG
LRFGTPAVTTRGFKEAECKELAGWICDILADLNNEAVIDAVREKVKAICKKLPVYGN

>112
MFSKQDQIQGYDDALLAAMNAEEQRQEDHIELIASENYTSKRVMQAQGSGLTNKYAEGYP
GKRYYGGCEHVDKVEALAIERAKQLFGADYANVQPHSGSSANGAVYLALLQAGDTILGMS
LAHGGHLTHGAKVSSSGKLYNAVQYGIDTNTGLIDYDEVERLAVEHKPKMIVAGFSAYSK
TLDFPRFRAIADKVGALLFVDMAHVAGLVAAGLYPNPIPFADVVTTTTHKTLRGPRGGLI
LAKSNEEIEKKLNAAVFPGAQGGPLMHVIAAKAVCFKEALEPGFKAYQQQVIENAQAMAQ
VFIDRGYDVVSGGTDNHLFLVSLIRQGLTGKDADAALGRAHITVNKNAVPNDPQSPFVTS
GLRIGTPAVTTRGFKVAQCVALAGWICDILDNLGDADVEADVAKNVAALCADFPVYR

>113
MFSRDLTIAKYDADLFAAMEQEAQRQEEHIELIASENYTSPAVMEAQGSVLTNKYAEGYP
GKRYYGGCEYVDVVEQLAIDRAKELFGADYANVQPHAGSQANAAVYLALLQGGDTILGMS
LAHGGHLTHGAAVSSSGKLYNAIQYGIDANGLIDYDEVERLAVEHKPKMIVAGFSAYSQI
LDFPRFREIADKVGAYLFVDMAHVAGLVAAGVYPNPVPFADVVTTTTHKTLRGPRGGLIL
ARANADIEKKLNSAVFPGAQGGPLEHVIAAKAICFKEALQPEFKAYQEQVVKNAQAMAEV
FIARGFDVVSGGTKNHLFLLSLIKQDISGKDADAALGKAFITVNKNSVPNDPRSPFVTSG
LRFGTPAVTTRGFKEAECKELAGWICDILADLNNEAVIDAVREKVKAICKKLPVYGA

>114
MFSKQDQIQGYDDALLAAINAEEQRQEDHIELIASENYTSKRVMQAQGSGLTNKYAEGYP
GKRYYGGCEHVDKVEALAIERAKQLFGADYANVQPHSGSSANSEVYLALLQAGDTILGMS
LAHGGHLTHGAKVSSSGKLYNAVQYGIDTRTGLIDYDEVERLAVEHKPKMIVAGFSAYSK
TLDFPRFRQIADKVGALLFVDMAHVAGLVAAGLYPNPLPYADVVTTTTHKTLRGPRGGLI
LAKANEEIEKKLNAAVFPGAQGGPLMHVIAAKAVCFKEALEPGFKAYQQQVIDNAQAMAG
VFIKRGYDVVSGGTDNHLFLVSLIRQGLTGKDADAALGRAHITVNKNAVPNDPQSPFVTS
GLRIGTPAVTTRGFKVTQCTELAGWICDILDHLGDADVEANVARQVAALCADFPVYR

>115
MAGKRPFAGARFHDEIERTAALIACRVFNAEHANLQPHSCSQANQSVYHALLEPGDNVLA
LNFKAGGHLTHGHKVNFSGMFFNFRHYGVDEATDLIDYDLAEQDAIRFKPKLIVCGSSSY
PRLFDARRLREISDKVGALLMFDLSHEAGLIACGAIPNPVPLADVATMSMDKTMRGAHGA
IILCTAKIAQKIDKGVHPGTQSSFPISRLTQTAQALLHSQTAEFREYANRVLDNALLLEQ
HFLCIPNLLVTGGTDKHYLVLNTKAAFGIDGVLAEQRLEAISVLSSRQTLPGDRTSRIDD
AGGIRLGTAWITSRGYELDEVSALATIIIEALSPSFDDAKKHHLLSRVNTLIATDKPKDV
WRNS

>116
MFSRDLTIAKYDADLFAAMEQEALRQEEHIELIASENYTSPAVMEAQGSVLTNKYAEGYP

GKRYYGGCEYVDVVEQLAIDRAKELFGADYANVQPHAGSQANSAVYLALLQGGDTILGMS
LAHGGHLTHGASVSSSGKLYNAVQYGIDGNGMIDYDEVERLAVEHKPKMIVAGFSAYSQI
LDFPRFRAIADKVGAYLFVDMAHVAGLVAAGVYPNPVPFADVVTTTTHKTLRGPRGGLIL
ARANADIEKKLNSAVFPGSQGGPLEHVIAAKAICFKEALQPEFKTYQQQVVKNAKAMAGV
FIERGFDVVSGGTENHLFLLSLIKQDISGKDADAALGRAFITVNKNSVPNDPRSPFVTSG
LRFGTPAVTTRGFKETECKELAGWICDILADLNNEAVIDAVREKVKAICAKLPVYGA

>117
MFSKQDQIQGYDDALLSAMNAEEQRQEDHIELIASENYTSKRVMQAQGSGLTNKYAEGYP
GKRYYGGCEHVDKVEQLAIERARQLFGADYANVQPHSGSQANAAVYLALLQAGDTVLGMS
LAHGGHLTHGAKVSFSGKLYNAVQYGIDTTTGLIDYDEVERIAVECQPKMLIAGFSAYSK
TLDFPRFRAIADKVGAYLFVDMAHVAGLVAAGLYPNPLPYADVVTTTTHKTLRGPRGGLI
LAKANEELEKKFNSAVFPGGQGGPLMHVIAAKAVCFKEAMEPGFKTYQQQVIDNAQAMAQ
VFITRGFDVVSGGTDNHLFLVSLIRQGLTGKEADAALGRAHITVNKNSVPNDPQSPFVTS
GLRIGTPAVTTRGFKVTQCIELAGWICDILDNLGAADVEANVASQVAALCADFPVYR

>118
MLKREMNIADYDAELWQAMEQEKVRQEEHIELIASENYTSPRVMQAQGSQLTNKYAEGYP
GKRYYGGCEYVDIVEQLAIDRAKELFGADYANVQPHSGSQANFAVYTALLEPGDTVLGMN
LAHGGHLTHGSPVNFSGKLYNIVPYGIDATGHIDYADLEKQAKEHKPKMIIGGFSAYSGV
VDWAKMREIADSIGAYLFVDMAHVAGLVAAGVYPNPVPHAHVVTTTTHKTLAGPRGGLIL
AKGGSEELYKKLNSAVFPGGQGGPLMHVIAGKAVALKEAMEPEFKTYQQQVAKNAKAMVE
VFLERGYKVVSGGTDNHLFLVDLVDKNLTGKEADAALGRANITVNKNSVPNDPKSPFVTS
GIRVGTPAITRRGFKEAEAKELAGWMCDVLDSINDEAVIERIKGKVLDICARYPVYA

>119
MFSKQDQIQGYDDALLAAMNAEEQRQEDHIELIASENYTSKRVMQAQGSGLTNKYAEGYP
GKRYYGGCEHVDKVEALAIERAKQLFGADYANVQPHSGSSANAAVYLALLQAGDTILGMS
LAHGGHLTHGAKVSSSGKLYNAVQYGIDTNTGLIDYDEVERLAVEHKPKMIVAGFSAYSK
TLDFPRFRAIADKVGALLFVDMAHVAGLVAAGLYPNPIPFADVVTTTTHKTLRGPRGGLI
LAKSNEEIEKKLNAAVFPGAQGGPLMHVIAAKAVCFKEALEPEFKAYQQQVIENAQAMAQ
VFIDRGYDVVSGGTDNHLFLVSLIRQGLTGKDADAALGRAHITVNKNAVPNDPQSPFVTS
GLRIGTPAVTTRGFKVAQCVALAGWICDILDNLGDADVEADVAKNVAALCADFPVYR

>120
MFNRDMNIADYDPELWQSITDEVQRQEDHIELIASENYTSPRVMEAQGSQLTNKYAEGYP
GKRYYGGCEYVDVAESLAIERAKSLFGADYANVQPHSGSQANAAVYQALCAPGDTILGMS
LAHGGHLTHGSHVSFSGKMYNAVQYGITPETGILDYAEIERLAVEHKPTMIIAGFSAYSG
IVDWAKFREIADKVGAYLFVDMAHVAGLVAAGLYPNPVPFADVVTTTTHKTLGGPRGGLI
LAKANEAIEKKLNSAVFPGQQGGPLMHVIAAKAVAFKECAEPEFAVYQQQVLDNAKAMVK
SFLARGYKIVSGGTENHLFLVDLIAQDITGKEADAALGNAHITVNKNSVPNDPRSPFVTS
GLRIGTPALARRGVNAQQSAELALWMCDVLDAIKDEAKLATTITAVKVKVAALCKACPVY
G

>121
FFSAHLAETDPEIAKAISQELGRQQHEIELIASENIVSRAVLEAQGSVLTNKYAEGYPGR
RYYGGCQFVDIAEELAIDRAKRLFGCGFANVQPNSGSQANQGVFMALMQPGDTFLGLDLA
AGGHLTHGAPPNVSGKWFKPVSYTVRREDQRIDMEQVERLAQEHKPKVIIAGGSGYPRHW
DFAKFREIADSVGAYFFVDMAHFAGLVAAGLHPSPFPHAHVATTTTHKTLRGPRGGMILT
NDEALAKKFNSAIFPGLQGGPLMHVIAAKAAAFGEALKPEFKIYAKQVIDNARALADTII

SGGYDITSGGTDNHLMLVDLQKKGLTGKAAEAALSRADITCNKNGVPFDPQKPTITSGIR
LGTPASTTRGFGVAEFKQVGSLIVQVLDGIAEKGDGGDAAVEAAVKEKVHALTDRFPIYA

>122
FFSQSLAERDASVRGAILKELERQQSQVELIASENIVSRAVLDAQGSVLTNKYAEGYPGK
RYYGGCEFADEVEALAIERVKRLFNAGHANVQPHSGAQANGAVMLALAKPGDTVLGMSLD
AGGHLTHGAKPALSGKWFNALQYGVSRDTMLIDYDQVEALAQQHKPSLIIAGFSAYPRKL
DFARFRAIADSVGAKLMVDMAHIAGVIAAGRHANPVEHAHVVTSTTHKTLRGPRGGFVLT
NDEEIAKKINSAVFPGLQGGPLMHVIAGKAVAFGEALTDDFKTYIDRVLANAQALGDVLK
AGGVDLVTGGTDNHLLLVDLRPKGLKGAQVEQALERAGITCNKNGIPFDPEKPTITSGIR
LGTPAGTTRGFGAAEFREVGRLILEVFEALRTNPEGDHATEQRVRREIFALCERFPIY

>123
MLKREMNIADYDAELWQAMEQEKVRQEEHIELIASENYTSPRVMQAQGSQLTNKYAEGYP
GKRYYGGCEYVDVVEQLAIDRAKELFGADYANVQPHSGSQANFAVYTALLQPGDTVLGMN
LAQGGHLTHGSPVNFSGKLYNIVPYGIDESGKIDYDEMAKLAKEHKPKMIIGGFSAYSGV
VDWAKMREIADSIGAYLFVDMAHVAGLIAAGVYPNPVPHAHVVTTTTHKTLAGPRGGLIL
AKGGDEELYKKLNSAVFPSAQGGPLMHVIAGKAVALKEAMEPEFKVYQQQVAKNAKAMVE
VFLNRGYKVVSGGTENHLFLLDLVDKNLTGKEADAALGRANITVNKNSVPNDPKSPFVTS
GIRIGSPAVTRRGFKEAEVKELAGWMCDVLDNINDEATIERVKAKVLDICARFPVYA

>124
SNAMSLEMFDKEIFDLTNKELERQCEGLEMIASENFTLPEVMEVMGSILTNKYAEGYPGK
RYYGGCEFVDEIETLAIERCKKLFNCKFANVQPNSGSQANQGVYAALINPGDKILGMDLS
HGGHLTHGAKVSSSGKMYESCFYGVELDGRIDYEKVREIAKKEKPKLIVCGASAYARVID
FAKFREIADEIGAYLFADIAHIAGLVVAGEHPSPFPYAHVVSSTTHKTLRGPRGGIIMTN
DEELAKKINSAIFPGIQGGPLMHVIAAKAVGFKFNLSDEWKVYAKQVRTNAQVLANVLMD
RKFKLVSDGTDNHLVLMSFLDREFSGKDADLALGNAGITANKNTVPGEIRSPFITSGLRL
GTPALTARGFKEKEMEIVSNYIADILDDVNNEKLQENIKQELKKLASNFIIYERAMF

>125
IFNNNLHETDKEINEIIKHEKLRQSSVIELIASENFVSPAVLEAQGALLTNKYAEGYPSK
RFYNGCEEVDKAENLAIERVKKLFNCKYANVQPHSGSQANQAVYLALLQPGDTVLGMSLD
SGGHLTHGAAPNMSGKWFNAVSYSVNKETYLIDYDEIERLADLHKPKLLIAGFSAYPRNI
DFAKFREIVDKVGAYFMADIAHIAGLVATGEHQSPIPYAHAVTSTTHXTLRGPRGGLILS
NDEEIGHKINSALFPGLQGGPLMHIIAAKAVAFLENLQPEYKSYIQQVISNAKALASSLQ
ERGYDILTGGTDNHIVLVDLRKDGITGKLAANSLDRAGITCNKNAIPFDETSPFITSGIR
LGTPACTTRGFKEKDFVLVGHMVADILDGLKNNEDNSALEQQVLNEVTKLIELFPFYG

>126
MFDRAQSTIANVDPEIFAAIEQENRRQEDHIELIASENYTSPAVMAAQGSQLTNKYAEGY
PGKRYYGGCEYVDVVEQLAIDRVKQLFGAEAANVQPNSGSQANQGVFFAMLKPGDTIMGM
SLAHGGHLTHGSPVNMSGKWFNVVSYGLNENEDIDYDAAEKLANEHKPKLIVAGASAFAL
KIDFERLAKIAKSVGAYLMVDMAHYAGLIAAGVYPNPVPHADFVTTTTHKSLRGPRGGVI
LMKAEYEKPINSAIFPGIQGGPLMHVIAAKAVAFKEALSPEFKEYQQKVVENARVLAETL
VKRGLRIVSGRTESHVMLVDLRAKHITGKAAEAALGAAHITVNKNAIPNDPEKPFVTSGI
RLGSPAMTTRGFGPAEAEQVGNLIADVLENPEDAATIERVRAQVAELTKRFPVYR

>127
MLKREMNIADYDAELWQAMEQEKVRQEEHIELIASENYTSPRVMQAQGSQLTNKYAEGYP

GKRYYGGCEYVDIVEQLAIDRAKELFGADYANVQPHSGSQANFAVYTALLEPGDTVLGMN
LAHGGHLTHGSPVNFSGKLYNIVPYGIDATGHIDYADLEKQAKEHKPKMIIGGFSAYSGV
VDWAKMREIADSIGAYLFVDMAHVAGLVAAGVYPNPVPHAHVVTTTTHKTLAGPRGGLIL
AKGGSEELYKKLNSAVFPGGQGGPLMHVIAGKAVALKEAMEPEFKTYQQQVAKNAKAMVE
VFLERGYKVVSGGTDNHLFLVDLVDKNLTGKEADAALGRANITVNKNSVPNDPKSPFVTS
GIRVGTPAITRRGFKEAEAKELAGWMCDVLDSINDEAVIERIKGKVLDICARYPVYA

>128
MFSRDVRLETYDPELAKAIAAEAGRQEDHVELIASENYCSPLVMEAQGSQLTNKYAEGYP
GKRYYGGCEFVDIAEQLAIDRIKQVFGADYANVQPHSGSQANQAVYLALLQPGDTILGMS
LAHGGHLTHGAKVNVSGKLFYAVQYGVNEQGLIDYDEVQRLATEHKPKMVVAGFSAYSQK
IDWARFRAIADSVGAYLFVDMAHIAGLVAAGVYPSPMEHAHVVTSTTHKTLRGPRGGIIV
AKGASEELQKKLQSIVFPGIQGGPLMHVIAAKAVAFKEALEPAFKTYQQQVVKNAQAMAN
TLIARGYKIVSGGTENHLMLVDMIGRDVSGKDAEAALGKAHITVNKNSVPNDPRSPFVTS
GLRLGTPAITTRGYQEQDSIDLANWIADVLDAPTDEAVLAKVRDAVTAQCKRYPVYG

>129
YAEKSPALWDAIRQEEKRQQNTIELIASENIVSDAVREAQGSVLTNKYAEGYPGRRYYGG
CQYIDQVEQLAIDYAKKLFNAKFANVQPHSGSQANMAVYQALLKPGDVILGMGMDAGGHL
THGAKVNFSGKEYKSYEYGLNVETEELDFDQIRKVALEVKPKLIVAGASAYSRIIDWQKF
RDIADEVGAYLMVDMAHIAGLVATDQHPSPIPVADIVTTTTHKTLRGPRGGMILSNNLEI
GKKINSALFPGIQGGPLEHVIAGKAQAFYEDLQPQFTDYIKQVVKNAKAMAEVFDESENI
RVVSGGTDNHLMIIDITDTGLTGKDAQNLLDFVNITTNKESIPGDKRSPFITSGLRIGTP
AITSRGFNEEDARKTASLIIEILSDPDNEATIEHVKKEVHELTKKHPVE

>130
MDVLAALERKPSLNLFPIENRLSPRASAALATDAVNRYPYSETPVAVYGDVTGLAEVYAY
CEDLAKRFFGARHAGVQFLSGLHTMHTVLTALTPPGGRVLVLAPEDGGHYATVTICRGFG
YEVEFLPFDRRTLEIDYAVLAARLSRRPADVIYLDASSILRFIDARALRLAAPDALICLD
ASHILGLLPVAPQTLVLDGGFDSISGSTHKTFPGPQKGLLVTDSDVVAEKVAARMPFTAS
SSHSASVGSLAISLEELLPHRTAYAHQVIANARALAGLLAERGFDVAGGAFGHTDTHQVW
VHFPEGNTPHEWGRLLTRANIRSTSVVLPSSAAPGLRLGTQELTRWGMTETDMAPVADLL
ERLLLRGDDAETVAKEVVELARAFPGVAFV

>131
MVAPLAEVDPDIAELLGKELGRQRDTLEMIASENFVPRSVLQAQGSVLTNKYAEGLPGRR
YYDGCEHVDVVENIARDRAKALFGADFANVQPHSGAQANAAVLHALMSPGERLLGLDLAN
GGHLTHGMRLNFSGKLYETGFYGVDATTHLIDMDAVRAKALEFRPKVLIAGWSAYPRILD
FAAFRSIADEVGAKLWVDMAHFAGLVAVGLHPSPVPHADVVSTTVHKTLGGGRSGLILGK
QEFATAINSAVFPGQQGGPLMHVIAGKAVALKIATTPEFTDRQQRTLAGARILADRLTAA
DVTKAGVSVVSGGTDVHLVLVDLRNSPFDGQAAEDLLHEVGITVNRNVVPNDPRPPMVTS
GLRIGTPALATRGFGEAEFTEVADIIATVLTTGGSVDVAALRQQVTRLARDFPLYGGLED
WSLAGR

>132
MSAPLAEVDPDIAELLAKELGRQRDTLEMIASENFVPRAVLQAQGSVLTNKYAEGLPGRR
YYGGCEHVDVVENLARDRAKALFGAEFANVQPHSGAQANAAVLHALMSPGERLLGLDLAN
GGHLTHGMRLNFSGKLYENGFYGVDPATHLIDMDAVRATALEFRPKVIIAGWSAYPRVLD
FAAFRSIADEVGAKLLVDMAHFAGLVAAGLHPSPVPHADVVSTTVHKTLGGGRSGLIVGK
QQYAKAINSAVFPGQQGGPLMHVIAGKAVALKIAATPEFADRQRRTLSGARIIADRLMAP

DVAKAGVSVVSGGTDVHLVLVDLRDSPLDGQAAEDLLHEVGITVNRNAVPNDPRPPMVTS
GLRIGTPALATRGFGDTEFTEVADIIATALATGSSVDVSALKDRATRLARAFPLYDGLEE
WSLVGR

>133
TLNDSLTAFDPDIAALIDGELRRQESGLEMIASENYAPLAVMQAQGSVLTNKYAEGYPGR
RYYGGCEFVDGVEQLAIDRVKALFGAEYANVQPHSGATANAATMHALLNPGDTILGLSLA
HGGHLTHGMRINFSGKLYHATAYEVSKEDYLVDMDAVAEAARTHRPKMIIAGWSAYPRQL
DFARFRAIADEVDAVLMVDMAHFAGLVAAGVHPSPVPHAHVVTSTTHKTLGGPRGGIILC
NDPAIAKKINSAVFPGQQGGPLEHVIAAKATAFKMAAQPEFAQRQQRCLDGARILAGRLT
QPDVAERGIAVLTGGTDVHLVLVDLRDAELDGQQAEDRLAAVDITVNRNAVPFDPRPPMI
TSGLRIGTPALAARGFSHNDFRAVADLIAAALTATNDDQLGPLRAQVQRLAARYPLYPEL
HRT

>134
SMSAPLAEVDPDIAELLAKELGRQRDTLEMIASENFVPRAVLQAQGSVLTNKYAEGLPGR
RYYGGCEHVDVVENLARDRAKALFGAEFANVQPHSGAQANAAVLHALMSPGERLLGLDLA
NGGHLTHGMRLNFSGKLYENGFYGVDPATHLIDMDAVRATALEFRPKVIIAGWSAYPRVL
DFAAFRSIADEVGAKLLVDMAHFAGLVAAGLHPSPVPHADVVSTTVHXTLGGGRSGLIVG
KQQYAKAINSAVFPGQQGGPLMHVIAGKAVALKIAATPEFADRQRRTLSGARIIADRLMA
PDVAKAGVSVVSGGTDVHLVLVDLRDSPLDGQAAEDLLHEVGITVNRNAVPNDPRPPMVT
SGLRIGTPALATRGFGDTEFTEVADIIATALATGSSVDVSALKDRATRLARAFPLYDGLE
EWSLVGR

>135
VRYQPLNELDPEVAAAIAGELARQRDTLEMIASENFVPRSVLQAQGSVLTNKYAEGYPGR
RYYGGCEQVDIIEDLARDRAKALFGAEFANVQPHSGAQANAAVLMTLAEPGDKIMGLSLA
HGGHLTHGMKLNFSGKLYEVVAYGVDPETMRVDMDQVREIALKEQPKVIIAGWSAYPRHL
DFEAFQSIAAEVGAKLWVDMAHFAGLVAAGLHPSPVPYSDVVSSTVHKTLGGPRSGIILA
KQEYAKKLNSSVFPGQQGGPLMHAVAAKATSLKIAGTEQFRDRQARTLEGARILAERLTA
SDAKAAGVDVLTGGTDVHLVLADLRNSQMDGQQAEDLLHEVGITVNRNAVPFDPRPPMVT
SGLRIGTPALATRGFDIPAFTEVADIIGTALANGKSADIESLRGRVAKLAADYPLYEGLE
DWTIV

>136
SSVGVLLRQDPELAEILFAEGRRQSTTLQLIAAENFTSPAVLAALGSPLANKYAEGYPGA
RHHGGCEIVDVAERLAAQRAQALFGAEHANVQSHSGSSAVLAAYAALLRPGDTVLALGLP
YGGHLTHGSPANFSGRWFDFVGYGVDAETGLIDHDQVRTLARARRPKAIVCGSIAYPRHL
DYAAFRDIADEVGAYLIADAAHPIGLVAGGAAPSPVPYADIVCATTHKVLRGPRGGMILC
GSELAERVDRAVFPFTQGGAQMHTIAAKAVAFGEAATPAFAAYAHQVVANARALAAHLAA
EGLVVTTGGTDTHLLTADPAPLGVDGKTARGLLAAAGIVLDCCALPHADARGLRLGTAAV
TTQGMGEREMRAVATLVAGVLRGTTDPAAARADVRDLTAEFPPYP

>137
MKHLPAQDEQVFNAIKNERERQQTKIELIASENFVSEAVMEAQGSVLTNKYAEGYPGKRY
YGGCEHVDVVEDIARDRAKEIFGAEHVNVQPHSGAQANMAVYFTILEQGDTVLGMNLSHG
GHLTHGSPVNFSGVQYNFVEYGVDKETQYIDYDDVREKALAHKPKLIVAGASAYPRTIDF
KKFREIADEVGAYFMVDMAHIAGLVAAGLHPNPVPYADFVTTTTHKTLRGPRGGMILCRE
EFGKKIDKSIFPGIQGGPLMHVIAAKAVSFGEVLQDDFKTYAQNVISNAKRLAEALTKEG
IQLVSGGTDNHLILVDLRSLGLTGKVAEHVLDEIGITSNKNAIPYDPEKPFVTSGIRLGT

AAVTSRGFDGDALEEVGAIIALALKNHEDEGKLEEARQRVAALTDKFPLYKE

>138
MKYLPQQDPQVFAAIEQERKRQHAKIELIASENFVSRAVMEAQGSVLTNKYAEGYPGRRY
YGGCEYVDIVEELARERAKQLFGAEHANVQPHSGAQANMAVYFTVLEHGDTVLGMNLSHG
GHLTHGSPVNFSGVQYNFVAYGVDPETHVIDYDDVREKARLHRPKLIVAAASAYPRIIDF
AKFREIADEVGAYLMVDMAHIAGLVAAGLHPNPVPYAHFVTTTTHKTLRGPRGGMILCQE
QFAKQIDKAIFPGIQGGPLMHVIAAKAVAFGEALQDDFKAYAKRVVDNAKRLASALQNEG
FTLVSGGTDNHLLLVDLRPQQLTGKTAEKVLDEVGITVNKNTIPYDPESPFVTSGIRIGT
AAVTTRGFGLEEMDEIAAIIGLVLKNVGSEQALEEARQRVAALTDPTSRSAAGT

>139
FDKEDYKAFDPELWNAIDAEAERQQNNIELIASENVVSKAVMAAQGTLLTNKYAEGYPGK
RYYGGTAVIDVVETLAIERAKKLFGAKFANVQPHSGSQANAAVYMSLIQPGDTVMGMDLS
AGGHLTHGAPVSFSGKTYNFVSYNVDKESELLDYDAILAQAKEVRPKLIVAGASAYSRII
DFAKFREIADAVGAYLMVDMAHIAGLVASGHHPSPVPYAHVTTTTTHKTLRGPRGGLILT
DDEDIAKKLNSAVFPGLQGGPLEHVIAAKAVALKEALDPAFKEYGENVIKNAAAMADVFN
QHPDFRVISGGTNNHLFLVDVTKVVENGKVAQNVLEEVNITLNKNSIPYEQLSPFKTSGI
RVGSPAITSRGMGEAESRQIAEWMVEALENHDKPEVLERIRGDVKVLTDAFPLY

>140
FDKEDFESFDPELWAAIHAEEIRQQQNIELIASENIVSKAVMAAQGSVLTNKYAEGYPGK
RYYGGTEAVDVVENLAIERAKELFGAKFANVQPHSGSQANAAAYMALIQPGDTVLGMDLN
AGGHLTHGASVNFSGKTYHFVPYGVNSETELLDYDEILKIAKQVQPKLIVAGASAYSRLI
DFAKFREIADSVGAKLMVDMAHIAGLVATGAHPNPLPYADVVTTTTHKTLRGPRGGMILT
NDEALAKKINSAIFPGTQGGPLEHVIAAKAVAFKEALDPEFTTYIEQVIKNTQAMAEEFA
KVEGLRLIAGGSDNHLLNLKVLDLGINGKEAQDLLDSVHITLNKEAIPDETLSPFKTSGV
RIGAAAITSRGFKEVEAKKVAQLVSEALVNHDNQEKLAEVRKAALELTRQFPL

>141
VWNRSLAETDPEIARAIALEITRQGAKLELIASENFVSRAVLEAQGSVLTNKYAEGYPGA
RYYGGCEYVDIVESVAIRRAKEIFGAGHANVQPHSGAQANMAAYFAFLEPGDTIMGMRLA
HGGHLTHGAKINFSGRYFRYVPYGVEEETGRIDYDRMHAIAREHRPKLIVGGASAYPREL
DFARMRAIADDVGALLMIDMAHIAGLIAAGLHMSPVPYADVVTTTTHKTLRGPRGGMILC
PEEYAAAIDKAVFPGIQGGPLMHVIAAKAVALGEAQRPEFKTYQEQIVKNARALAQALQE
RGFELVAGGTDTHLILVDLRNKGLTGAVAEDLLDRVDVTVNKNMVPFDPQPPRVTSGIRI
GTPAVTTRGMKEDSMVQIAEVISLTLDHPEEGAVQARAKAIVAELCAAHPFLKL

>142
MDIEIIRKTDPEIADAIEKELIRQRNKIELIASENFVSRAVMEAMGSPLTNKYAEGYPNK
RYYGGCEYVDIAEELARERLKKLFGAEHANVQPHSGAQANMAAYFALIKPGDTVLGMDLA
HGGHLTHGSKVNFSGQIYNFVSYGVREDTGYIDYDEVERVAKKHKPKLIVAGASAYPRII
DFKRFREIADKVGAYLMVDMAHIAGLVAAGLHPNPVPYADVVTTTTHKTLRGPRGGAILC
KEEYAKAIDKALFPGTQGGPLMHIIAAKAVCFKEALTDEFKEYQKRIVENAKALANALME
RGINLVSGGTDNHLMLLDLRNTGITGKELETRLDEVNITCNKNAIPFDPLGPNVTSGVRL
GTPAVTTRGMKPEDMVEIADIIVNVIRDENYKEKAKERVANLLKKYPLYED

>143
MRHLFNTDAEIYEAIVKEYERQFYHLELIASENFTSLAVMEAQGSVMTNKYAEGLPHKRY
YGGCEFVDIAEDLAIERAKALFDAEHANVQPHSGTQANMAVYMAVLKPGDTIMGMDLSHG

GHLTHGAKVNFSGKIYNAVYYGVHPETHLIDYDQLYRLAKEHKPKLIVGGASAYPRVIDW
AKLREIADSVGAYLMVDMAHYAGLIAGGVYPNPVPYAHFVTSTTHKTLRGPRSGFILCKK
EFAKDIDKSVFPGIQGGPLMHVIAAKAVAFKEAMSQEFKEYARQVVANARVLAEEFIKEG
FKVVSGGTDSHIVLLDLRDTGLTGREVEEALGKANITVNKNAVPFDPLPPVKTSGIRLGT
PAMTTRGMKEDQMRIIARLISKVIKNIGDEKVIEYVRQEVIEMCEQFPLYPELREEIN

>144
MVSTLKRDEALFELIALEEKRQREGLELIASENFVSKQVREAVGSVLTNKYAEGYPGARY
YGGCEVIDRVESLAIERAKALFGAAWANVQPHSGSQANMAVYMALMEPGDTLMGMDLAAG
GHLTHGSRVNFSGKLYKVVSYGVRPDTELIDLEEVRRLALEHRPKVIVAGASAYPRFWDF
KAFREIADEVGAYLVVDMAHFAGLVAAGLHPNPLPYAHVVTSTTHKTLRGPRGGLILSND
PELGKRIDKLIFPGIQGGPLEHVIAGKAVAFFEALQPEFKEYSRLVVENAKRLAEELARR
GYRIVTGGTDNHLFLVDLRPKGLTGKEAEERLDAVGITVNKNAIPFDPKPPRVTSGIRIG
TPAITTRGFTPEEMPLVAELIDRALLEGPSEALREEVRRLALAHPMP

>145
TNFDFLAQGDPAIAAIIGRELQRQQEHLELIASENFASPAVMAAQGSVLTNKYAEGLPSK
RYYGGCEFVDQAEELAIERAKELFGAAHANVQPHSGAQANFAVFLTLLQPGDTFLGMDLS
HGGHLTHGSPVNVSGKWFNAGHYGVNRETERLDYDAIRELALQHRPKLIICGYSAYPRTI
DFAKFREIADEVGAYLLADMAHIAGLVAAGLHPSPIPHCDVVTTTTHKTLRGPRGGLILT
RDAELGKKLDKSVFPGTQGGPLEHVIAAKAVAFGEALRPEFKTYSAQVIANAQALARQLQ
ARGLKIVSDGTDNHLLLVDLRSIGMTGKVADLLVSDVNITANKNTVPFDPESPFVTSGIR
LGTAAMTTRGFKEAEFAIVADIIADRLLNPEDSSMEDSCRRRVLELCQRFPLYPHLS

>146
THIDWLVQTDPLVAEMVQREVQRQQQHLELIASENFTSPAVMAAQGTVLTNKYAEGLPGK
RYYGGCEFVDEVEQLAIDRAKELFGAAHANVQPHSGAQANFAVFLALLNPGDTIMGMDLS
HGGHLTHGSPVNVSGKWFNVVHYGVHPETERLDMDQVRDLARQHRPKLIICGYSAYPRVI
PFAEFRQIADEVGAYLMADIAHIAGLVASGYHPNPVPLCDVVTTTTHKTLRGPRGGLILT
RDEDLGKKLDKAVFPGTQGGPLEHVIAAKAVAFGEALKPEFKAYCGQVIRNAQALAAGLQ
ARQLRLVSGGTDNHLMLIDLRSVNLTGKEADRLMGEIHITTNKNTIPFDPASPFVTSGLR
LGTPALTTRGFTEVEFAEVAEIISDRLHAPEDEAIKNRCRERVAALCAQFPLYPHLQ

>147
FNNDPLQKYDKELFDLLEKEKNRQIETINLIASENLTNTAVRECLGDRISNKYSEGYPHK
RYYGGNDYVDKIEELCYKRALEAFNVSEEEWGVNVQPLSGSAANVQALYALVGVKGKIMG
MHLCSGGHLTHGFFDEKKKVSITSDLFESKLYKCNSEGYVDMESVRNLALSFQPKVIICG
YTSYPRDIDYKGFREICDEVNAYLFADISHISSFVACNLLNNPFTYADVVTTTTHKILRG
PRSALIFFNKKRNPGIDQKINSSVFPSFQGGPHNNKIAAVACQLKEVNTPEFKEYTKQVL
LNSKALAECLLKRNLDLVTNGTDNHLIVVDLRKYNITGSKLQETCNAINIALNKNTIPSD
VDCVSPSGIRIGTPALTTRGCKEKDMEFIADMLLKAILLTDELQQKYGKKLVDFKKGLVN
NPKIDELKKEVVQWAKNLPFA

>148
MFNNEPLEQIDKELHDILADEEKRQRETINLIASENLTNGAVRECLGNRVSNKYSEGYPK
KRYYGGNDFIDKIEELCQKRALEAFNVSDEEWGVNVQPLSGSAANVQALYALVGVKGKIM
GMHLCSGGHLTHGFFDEKKKVSITSDMFESKLYKCNSQGYVDLDAVREMALSFKPKVIIC
GYTSYPRDIDYQQFRQICDEVNAYLFADISHISSFVACNILNNPFLHADVVTTTTHKILR
GPRSALIFFNKKRNPGIEQKINSAVFPSFQGGPHNNKIAAVACQLKEVHSPAFKEYTQQV
LLNSKALAKALISKQIDLVTNGTDNHLIVVDLRKFSITGSKLQETCNAINVSLNKNTIPS

DVDCVSPSGVRIGTPAMTTRGAKEKDMEFIADVLARAIKITVDLQEQYGKKLVDFKKGLP
GNAQLQQLKQEVVTWAGALPFP

>149
MPYTLSDAHHKLITSHLVDTDPEVDSIIKDEIERQKHSIDLIASENFTSTSVFDALGTPL
SNKYSEGYPGARYYGGNEHIDRMEILCQQRALKAFHVTPDKWGVNVQTLSGSPANLQVYQ
AIMKPHERLMGLYLPDGGHLSHGYATENRKISAVSTYFESFPYRVNPETGIIDYDTLEKN
AILYRPKVLVAGTSAYCRLIDYKRMREIADKCGAYLMVDMAHISGLIAAGVIPSPFEYAD
IVTTTTHKSLRGPRGAMIFFRRGVRSINPKTGKEVLYDLENPINFSVFPGHQGGPHNHTI
AALATALKQAATPEFKEYQTQVLKNAKALESEFKNLGYRLVSNGTDSHMVLVSLREKGVD
GARVEYICEKINIALNKNSIPGDKSALVPGGVRIGAPAMTTRGMGEEDFHRIVQYINKAV
EFAQQVQQSLPKDACRLKDFKAKVDEGSDVLNTWKKEIYDWAGEYPLAV

>150
MATYALSESHKELMEVHLADFDPEIAEIIKKEIQRQRESILLIASENVTSRAVFDALGTP
MSNKYSEGYPGARYYGGNQHIDAVELTCQARALKVFNLDPEKWGVNVQTLSGSPANLQVY
QALMKPHDRLMGLDLPHGGHLSHGYQTPQRKISAVSTYFETFPYRVNSETGIIDYDTLEA
NAQLYRPKILVAGTSAYCRLIDYARMRKIADSVGAYLVVDMAHISGLIAAGVIPSPFEYA
DVVTTTTHKSLRGPRGAMIFFRKGVRSTDPKTGKEILYDLEGPINFSVFPGHQGGPHNHT
ITALAVALKQASTPEFRQYQEQTIKNAKALEVAFKEYGYKLVADGTDSHMVLVDLRPNGI
DGARVETVLEQINIACNKNAVPGDKSALSPGGIRVGAPAMTTRGLGEEDFKRVVGYIDQA
IKISKSIQASLPKEANKLKDFKAKASSETIPEILNLRKEISAWASTFPLPV

>151
MATYALSPAHRNQMEVSLVDSDPEIAQIMEKEIQRQRESIVLIASENFTSHAVFDALGSP
MSNKYSEGYPGARYYGGNQHIDAIELTCQARALKAFNLDPAKWGVNVQCLSGSPANLQVY
QALMRPHDRLMGLDLPHGGHLSHGYQTPARKISAVSTYFETFPYRVNLETGIIDYDQLEA
NAELYRPKCLVAGTSAYCRLIDYARMRKIADKVGAYLIVDMAHISGLIAAGVIPSPFEYA
DVVTTTTHKSLRGPRGAMIFFRKGVRSTDPKTGKDIMYDLEGPINFSVFPGHQGGPHNHT
ITALAVALKQVDTPEFKQYQQQVIKNAKALEEEFKALGHKLVSDGTDSHMVLVDLRNKSL
DGARVEAVLEQINIACNKNSIPGDKSALTPCGIRIGAPAMTTRGMGEEDFKRIARYIDQS
INLCKKVQSELPKEANKLKDFKAKVASETVPEILSLRKEVAEWASTFPLPV

>152
HSNAAQTQTGEANRGWTGQESLSDSDPEMWELLQREKDRQCRGLELIASENFCSRAALEA
LGSCLNNKYSEGYPGKRYYGGAEVVDEIELLCQRRALEAFDLDPAQWGVNVQPYSGSPAN
LAVYTALLQPHDRIMGLDLPDGGHLTHGYMSDVKRISATSIFFESMPYKLNPKTGLIDYN
QLALTARLFRPRLIIAGTSAYARLIDYARMREVCDEVKAHLLADMAHISGLVAAKVIPSP
FKHADIVTTTTHKTLRGARSGLIFYRKGVKAVDPKTGREIPYTFEDRINFAVFPSLQGGP
HNHAIAAVAVALKQACTPMFREYSLQVLKNARAMADALLERGYSLVSGGTDNHLVLVDLR
PKGLDGARAERVLELVSITANKNTCPGDRSAITPGGLRLGAPALTSRQFREDDFRRVVDF
IDEGVNIGLEVKSKTAKLQDFKSFLLKDSETSQRLANLRQRVEQFARAFPMPGFDEH

>153
PRDAALWSSHEQMLAQPLKDSDAEVYDIIKKESNRQRVGLELIASENFASRAVLEALGSC
LNNKYSEGYPGQRYYGGTEHIDELETLCQKRALQAYGLDPQCWGVNVQPYSGSPANFAVY
TALVEPHGRIMGLDLPDGGHLTHGFMTDKKKISATSIFFESMAYKVNPDTGYIDYDRLEE
NARLFHPKLIIAGTSCYSRNLDYGRLRKIADENGAYLMADMAHISGLVVAGVVPSPFEHC
HVVTTTTHKTLRGCRAGMIFYRRGVRSVDPKTGKEILYNLESLINSAVFPGLQGGPHNHA
IAGVAVALKQAMTPEFKEYQRQVVANCRALSAALVELGYKIVTGGSDNHLILVDLRSKGT

DGGRAEKVLEACSIACNKNTCPGDKSALRPSGLRLGTPALTSRGLLEKDFQKVAHFIHRG
IELTVQIQDDTGPRATLKEFKEKLAGDEKHQRAVRALRQEVESFAALFPLPGLPGF

>154
DRDATLWASHEKMLSQPLKDSDAEVYSIIKKESNRQRVGLELIASENFASRAVLEALGSS
LNNKYSEGYPGQRYYGGTEFIDELEMLCQKRALQAYHLDPQCWGVNVQPYSGSPANFAVY
TALVEPHGRIMGLDLPDGGHLTHGFMTDKKKISATSIFFESMPYKVYPETGYINYDQLEE
NASLFHPKLIIAGTSCYSRNLDYARLRKIADDNGAYLMADMAHISGLVAAGVVPSPFEHC
HVVTTTTHKTLRGCRAGMIFYRKGVRSVDPKTGKETYYELESLINSAVFPGLQGGPHNHA
IAGVAVALKQAMTTEFKIYQLQVLANCRALSDALTELGYKIVTGGSDNHLILMDLRSKGT
DGGRAEKVLEACSIACNKNTCPGDKSALRPSGLRLGTPALTSRGLLEEDFQKVAHFIHRG
IELTLQIQSHMATKATLKEFKEKLAGDEKIQSAVATLREEVENFASNFSLPGLPDF

>155
DADLWSSHDAMLAQPLKDSDVEVYNIIKKESNRQRVGLELIASENFASRAVLEALGSCLN
NKYSEGYPGQRYYGGTEFIDELETLCQKRALQAYKLDPQCWGVNVQPYSGSPANFAVYTA
LVEPHGRIMGLDLPDGGHLTHGFMTDKKKISATSIFFESMPYKVNPDTGYINYDQLEENA
RLFHPKLIIAGTSCYSRNLEYARLRKIADENGAYLMADMAHISGLVAAGVVPSPFEHCHV
VTTTTHKTLRGCRAGMIFYRKGVKSVDPATGKEILYNLESLINSAVFPGLQGGPHNHAIA
GVAVALKQAMTLEFKVYQHQVVANCRALSEALTELGYKIVTGGSDNHLILVDLRSKGTDG
GRAEKVLEACSIACNKNTCPGDRSALRPSGLRLGTPALTSRGLLEKDFQKVAHFIHRGIE
LTLQIQSDTGVAATLKEFKERLAGDKYQAAVQALREEVESFASLFPLPGL

>156
FFNSSVHDTDPLIAQALDDERARQKNQIELIASENIVSQAVLDALGHEMTNKTLEGYPGN
RFHGGGQFVDVVEQAAIDRAKQLFNCGYANVQPHSGTQANLAVFFLLVKPGDRILSLDLA
AGGHLSHGMKGNLSGRWFEAHNYNVDPQNEVINYDEMERIAEEVKPKLLITGGSAYPREL
DFARMAQIAKKVGAFFMVDMAHIAGLVAGGAHPSPFPHADIVTCTTTKTLRGPRGGLILT
NNEEWYKKLQTAVFPGVQGSLHSNVLAAKAICLGEALRPEFRDYVAQVVKNAKVLAETLT
SRGIRIVSGGTDTHIVLLDLSSKGLNGKQAEDALARANITSNKNPIPNDSPRPAEWVGMR
LGVSAATTRGMKEDEFRKLGNVVADLLEAESAGNGPEAAEKAKVTVRELTEAFPVYAH

>157
YFNTPVHERDPLVAQALDNERKRQQDQIELIASENIVSRAVLDALGHEMTNKTLEGYPGN
RFHGGGQFVDVVEQAAIDRAKELFGCAYANVQPHSGTQANLAVFFLLLKPGDKVLSLDLA
AGGHLSHGMKGNLSGRWFESHNYNVDPETEVIDYDEMERIAEEVRPTLLITGGSAYPREL
DFERMGKIAKKVGAWFLVDMAHIAGLVAGGAHPSPFPHADIVTCTTTKTLRGPRGGLILT
NNEAWFKKLQSAVFPGVQGSLHSNVLAAKAVCLGEALRPDFKVYAAQVKANARVLAETLI
ARGVRIVSGGTDTHIVLVDLSSKGLNGKQAEDLLARANITANKNPIPNDSPRPAEWVGMR
LGVSAATTRGMKEDEFRTLGTVIADLIEAEAAGNADGVVEGAKAKVATLTAAFPVYAH

>158
IGWDALTRKDPALGALLDAEAAYQTAHLSMVASASVAAPSVLCAHGTVMSNVTAEGYPGA
RYHPGAVHFDEIERLAVERARHVFGATYANVQPHSCSSANLAVLGALLPRGGRILALDLG
SGGHLTHGADASVSGKYYDVAHYGVEPDGRLDYDRILDRALTHRPDVVIAGASAYPRWID
FSRFRAVADRVGAWLLADISHIAGLVAAGLHPSPVDHAHATTTSTYKQLGGPRGGLILLG
KDHRATAPDGRTELWKLMQRAVFPGFQGTPDPSAIAAKARALAIASGGAFQATMKRVVDD
AKALADALVELGYDVLTGGTDNHMVLVDVRAAGLTGIVAERALEECGILVNRNKIPGDPL
PPGVGGGLRFGTNILAQRGMGPFEVRECARIVHNVLSSIQAGDATHYELDGALRDAVRDR
VRALCARFPLQFDDE

>159
MKGPSEFPREDFASDNHAEAHPAVLAALQAAAHGPAPAYGGDRWTAAAEAGLRDLAGPAA
VPLLVFNATAANVLGLSVLLGPGDAIICAEHSHLAMDECGAVEAMLGRKLLPVPAPDGKL
TPAAIEALLQRCTDVRTPRPSTVALTQATELGTCYTLAELAELREFCDRRGLRIHMDGAR
LANAVAHLNCEVADIARFADALSFGFTKNGALGVDALLLMTPGTAERAPYLRRQQGQLAS
KMRFLAAQVTALVDGGLWLDNARHANGMAGHLAAELEPLPGLRLTQRVQSNAVFVELPRA
AADSLRERWAFHLSPTGEARLMTSHATGRTDVSDLVAEVKTCLKQ

>160
MLVASSSVAPPSVLACAGSSLANLTMEGYPGRRYHAGAEVADEVERLALDRARALFGARA
ANVQPHSGSSANLAVLTGLLEPGDTILGLGLDCGGHLSHGSAASVTGRYFRSVHYRTGAD
DLLDYEQIAGLAGEHRPKVIIAGASAYPRQIDFARFREIADTVGAYLIADISHIAGLVAA
GLHPSPVDHAHLTTTSTYKQLYGPRGGLILLGRDADAPGPDGRLPLRKLADRAVFPLVQG
TPDLGNVAAKARALHAAAQPAFRALMARVLDTATAIAEQLDRRGLCVITGGTDTHMVLAD
LRPEHVSGADAEEALEACGILVNRNRVPGDDTPPRVTGGIRIGTNTLAARGVSPETAAQC
AELVADVVEELRTAGAVHPSTIAKIRAEVRRICAEHPVPGYAKPRIP

>161
MTVGAGGKTSADADPLMLVRAIADADRRAAHALNLVPSENRISPLASLPLASDFYNRYFF
NTDGDPLFWEFRGGEDIAHIEALGAAALRRMASARYCNVRPISGMSAMILTVAALSPPGS
TVVSVDQNSGGHYATPALLGRLGRRSRLLNCKDGEVDESELAEVLAPGDVALVYVDVQNC
VRVPDFRRMSDVIREVSPGTRLYVDASHYLGLVLGGLLANPLDCGADAFGGSTHKSFPGP
HKGVIFTNAEDVDESLRSAQFDLVSSHHFAETLALSLAALEVEDRMGDYARATNDNARRL
AGALADAGFRVYGDSATGYTDTHQVWVELDGVAAAYALSNRLAEGGIRVNLQSSMPGMSG
VHLRLGSNEVTFEGAGPQAIEELAGALVTARERALGPRTVHEIRGRFGAPFYTDPEKLKV
EAGL

>162
LLALLGEIEKEQRINEAAVNLVPSENRISPWAGAPLRTDFYNRYFFNDSLDPQGWQFRGG
EGIGRLEKELALPALRRLGRADHVNIRPVSGMSAMLVVLLGLGGEPGDGVVCVDAETGGH
YATGRQIAMLGRRPLPVRVVAGRVDLDALRTALTSCHVPLVYLDLQNSLWELDVAGVAEV
IARTSPRTVLHVDCSHTLGLILGGSHKNPLDLGADTTGGSTHKTFPGPQKGVLFTRDENL
SRKIRDAQFFTISSHHFAETLALALAAAEFEHFGAAYSRQVLINARAFAHRLRERGFGVV
EGGPQLTDTHQVWVRLPLEESADAFSAQLASLGIRVNVQTELPDIPEPALRLGVSEITLN
GGREPAMETLAEIFALVRAGEATKAVDLFQVLPHEMGEPYFFTGLP

>163
MTVGADGKTAADVDPLMLGRAIVDADRRAAHALNLVPSENRISPLAALPLGSDFYNRYFF
NTAGDPLFWEFRGGEDIAHIEALGAQALRRMASAQYCNVRPISGMSAMILTVAALSAPGR
TVVSIDQNSGGHYATPALLGRMGRHSRLLGCKDGQVDESELADVLAPGDVDLVYVDVQNC
VRVPDFGLMADVVNQVSPATRLYVDASHYLGLVLGGLVENPLACGADAFGGSTHKSFPGP
HKGVIFTNAEDVDESLRSAQFDMVSSHHFAETLALSLSALEVEPRIGDYAWATTDNARRL
ACALADAGFRVYGDSRSGYTDTHQVWVELDGTADAYALSNRLAEAGIRVNLQSSMPGMSG
VHLRLGSNEVTFEGAGPQAIEQLAGALATARERALEPRTVSEIRGRFGAPFYTDPEKLKL
EAGL

>164
MTVRAAGKTSADADPLALARAIADADRRAASALNLVPSENRVSPLASLPLASDFYNRYFF
NTDGDPLFWEFRGGEDIAHIEALGAAALRRMAMARYCNLRPISGMSAMILTVAALSKPGS

TVVSVDQDSGGHYATPALLGRLGRRSRLLTCKDGAVDESELADVLAPGGVDLVYVDVQNC
VRVPDFRLMSDVIRNVSPGTRLYVDASHYLGLVLGGLVDNPLDCGADAYGGSTHKSFPGP
HKGVIFTNAEDVDESLRSAQFDLVSSHHFAETLALSLAALEVEDRIGDYARATNDNARRL
AGALAEAGFRVCGDTGTGYTDTHQVWVELAGTDEAYALSNRLAEAGIRVNLQSSMPGMSG
VHLRLGSNEVTFEGAGPQAIEELAGALVQARERALGPHTVSEIRGRFGAPFYTDPEKLKV
EAGL

>A
MNARKAPEFPAWPQYDDAERNGLVRALEQGQWWRMGGDEVNSFEREFAAHHGAAHALAVT
NGTHALELALQVMGVGPGTEVIVPAFTFISSSQAAQRLGAVTVPVDVDAATYNLDPEAVA
AAVTPRTKVIMPVHMAGLMADMDALAKISADTGVPLLQDAAHAHGARWQGKRVGELDSIA
TFSFQNGKLMTAGEGGAVVFPDGETEKYETAFLRHSCGRPRDDRRYFHKIAGSNMRLNEF
SASVLRAQLARLDEQIAVRDERWTLLSRLLGAIDGVVPQGGDVRADRNSHYMAMFRIPGL
TEERRNALVDRLVEAGLPAFAAFRAIYRTDAFWELGAPDESVDAIARRCPNTDAISSDCV
WLHHRVLLAGEPELHATAEIIADAVARA

>B
MGSSPDAGIDFPAWPQHDDAERAALLRALDQGQWWRVGGSEVDEFEREFAEYHGAGHALA
VTNGTHALELALQVLDVGPGTEVIVPAFTFISSSQAVQRLGAVAVPVDVDPDTYCLDVAA
AEDAVTSRTSAIMPVHMAGQFADMDRLDKLSASTGVPVVQDAAHAHGAHWRGKRVGELGS
IATFSFQNGKLMTAGEGGAVLFADQAQWEKAFVLHSCGRPKGDRGYFHLTSGSNFRMNEF
SAAVLRAQLGRLDSQIATRQARWPVLSALLAGIDGVVPQTVDPRSDRNPSYMAMFRMPGV
TEERRNAVVDELVRRGIPAFMAFRAVYRTQAFWETGAPDLTPEELAARCPVSEEITRDCV
WLHHRVLLGAEEQVRRLAAVVADVVAGA

>C
MNARQAPEFPRWPQYDETERDGLIRALEQGQWWRMGGDEVDSFEREFAEHHGAPHALAVT
NGTHALELALQCLGVGPGTEVIVPAFTFISSSQAAQRLGAVAVPVDVHPDTYCIDAAAVA
AAVTPRTRAIMPVHMAGLIADMDALAKISADTGVPLLQDAAHAHGARWQGKRVGELGSVA
AFSFQNGKLMTAGEGGALLFPDTELYEAAFLRHSCGRPRDDRRYLHRTAGSNLRMNEFSA
AVLRAQLSRLDQQIALREERWTLLSRLLAAIDGVVPQGGDVRVDRKSHYMAMFRVPGLGE
ERRNALVDRLVEAGLPAFAAFRAIYRTDAFWEIGAPDETPDAIAARCPHTEAISQDCVWL
HHRVLLAGEAEMHATAEIIADLVARA

>D
MLDKELDLDFPAWPQYDDTERAGLVRALEQGQWWRIGGSEVDEFEREFAEANGAPHALAV
TTGTHALELALQVLGVGPGTEVVVPAFTFISSSQAAQRLGAVAVPVDVDLDTYCLDPEAV
AAAITPRTAAIMPVHMAGQICDMDALGKLSADSGVPLLHDAAHAHGGRWRDQGVSALGTM
AAFSFQNGKLMTAGEGGAVTFPDSEQYETAFLRHSCGRPRTDRTYRHQTSGSNFRMNEFT
ASVLRAQLARLDGQIDTREQRWPVLAGQLARITGVLPQATDDRCTRNPHYMAMFRVPGIS
EQQRNELVDALVARGLPAFAAFRAIHRTKAFWETGAPDEPVEAIARRCPNSEALSTDCVW
LHHRTLLGTEEQMHAVAEVVSDALAAS

>E
MTPTSGDDVLSFPSWPQHGAEERAGLLRALDQKGWWRDAGQEVDLFEREFADHHGAPHAI
ATTNGTHALELALGVMGIGPGDEVIVPAFTFISSSLAVQRMGAVPVPADVRPDTYCLDAD
AAAALVTPRTKAIMPVHMAGQFADMDALEKLSVATGVPVLQDAAHAHGAQWQGRRVGELG
SIAAFSFQNGKLMTAGEGGALLLPDDESFHEAFLQHCCGRPPGDRVYRHLTQGSNYRMNE
FSASVLRAQLKRLKDQLRIREERWAQLRTALAAIDGVVPQGRDERGDLHSHYMAMVRLPG
ISARRRLALVDALVERGVPAFVGFPPVYRTEGFARGPAPADAEELAKSCPVAEEIGSDCL

WLHHRVLLADVTTLDRLAEVFSGLVGAL

>F
MSSGVQLGSAFRVWPQYDDAERTGLIRALEQGQWWRMGGGEVERFEREFAEYHGGEHALA
VTNGTHALELALEVMGVGPGTEVIVPAFTFISSSQAAQRLGAVVVPVDVDPETYCIDPAE
AAKAITPRTRAIMPVHMAGQLADMDALEKVAADSGVPLIQDAAHAQGATWNGRRLGELGS
VAAFSFQNGKLMTAGEGGAVLFPTAEMAEHAFLRHSCGRPRNDRGYFHRTSGSNFRLNEF
SASVLRAQLARLDGQIRTREERWPLLSSLLAEIPGVVPQRLDRRPDRNPHYMAMFRVPRI
TEERRARVVDTLVERGVPAFVAFRSVYRTDAFWEMGAPDLSVDELARLPPLRGLTTDCLW
LHHRTLLGTEEQMHEVAAVIADVLGS

>G
MNARPAPEFPTWPQYDDEERTGLIRALEQGQWWRMGGEEVSSFEQEFADFHGAPHAFAVT
NGTHAFELALQVMGAGPGTEVIVPAFTFISSSQAAQRIGAVAVPVDVDPDTYNIDVAAAA
AAVTPRTRVIMPVHMAGLMADMDALGKLSADTGVAILQDAAHAQGARWQGKRVGELGTVA
AFSFQNGKLMTAGEGGAVLFPENDLYEAAFLRHSCGRPRTDRHYKHQVAGTNMRLNEFSA
AVLRAQLRRLPAQTELRDRRWALLSRLLGAVDGVVPQGGDVRADQNTHYMAMFRIPGITE
ADRNTLVDRLVEAGLPAFAAFRASTAPTPSGENRRPRRRPWSSVAERCPHSRGHQQRTAS
GCTTGSSSPTSGTWNSAAEIIADAVAAI

>H
MGSSPDAGIDFPAWPQHDDAERAALLRALDQGQWWRVGGSEVDEFEREFAEYHGAGHALA
VTNGTHALELALQVLDVGPGTEVIVPAFTFISSSQAVQRLGAVAVPVDVDPDTYCLDVAA
AEDAVTSRTSAIMPVHMAGQFADMDRLDKLSASTGVPVVQDAAHAHGAHWRGKRVGELGS
IATFSFQNGKLMTAGEGGAVLFADQAQWEKAFVLHSCGRPKGDRGYFHLTSGSNFRMNEF
SAAVLRAQLGRLDSQIATRQARWPVLSALLAGIDGVVPQTVDPRSDRNPSYMAMFRMPGV
TEERRNAVVDELVRRGIPAFMAFRAVYRTQAFWETGAPDLTPEELAARCPVSEEITRDCV
WLHHRVLLGAEEQVRRLAAVVADVVAGA

>I
MNARRTPEFPTWPQYDDGERTGLIRALEQGQWWRMGGSEVDSFEGEFADFHGAPHALAVT
NGTHALELALQCLGVGPGTEVIVPAFTFISSSQAAQRLGAVAVPVDVDLDTYNIDVAAAA
SAVTPLTKAIMPVHMAGLIADMDALGELSADTGVPLLQDAAHAHGARWQGKRVGELGTVA
SFSFQNGKLMTAGEGGALLLPDEETYEAAFLRHSCGRSRTDRRYMHQTAGTNMRLNEFSA
AVLRAQLGRLDAQITLRDQRWTLLSRLLGEIDGVVPQGSDPRADRNSHYMAMFRIPGISE
EARNALVDTLVEAGLPAFAAFRAIYRTDAFWETAAPDTTVDKLAESCPHTEAISTDCIWL
HHRVLLASEEALHTTAEIIADAVAAR

>J
MRLRSELPAWPQYGDEEREALIRALDQGQWWRIGGGEVDAFEAEFAAAHGSEHALAVTNG
THALELALEVLGVGADSEVIVPAFTFISSSQAAQRLGAVAVPVDVDPDTYCIDPSAVEAA
IGPKTRAIMPVHMAGQMCDMDALGKLSADSGVPLIQDAAHAHGARWRGQKVGELGSVAAF
SFQNGKLMTAGEGGAVLFPDAEMYERGFVRHSCGRPRTDRGYFHRTSGSNFRLNEFSASV
LRAQLTRLDGQITTREQRWPVLSRLLAEIPGVVPQSRDDRGDRNPHYMAMFRVPGITEER
RAKVVDTLIERGVPAFVAFRAVYRTDAFWEVAAPDLTVDELARRCPHSEALTRDCLWLHH
RVLLGSEEQMHEVAAVVADVLAGA

Supplementary References

1. Herbert, R. B. & Knaggs, A. R. Biosynthesis of the antibiotic obafluorin from *p*-aminophenylalanine and glycine (glyoxylate). *J*. *Chem*. *Soc*., *Perkin Trans*. *1* 109-113 (1992).
2. Voisard, C., Bull. C.T., Keel, C., Laville, J., Maurhofer, M., Schnider, U., Défago, G. & Haas, D. Biocontrol of root diseases by *Pseudomonas fluorescens* CHA0: current concepts and experimental approaches. In Molecular Ecology of Rhizosphere Microorganisms (O'Gara, F., Dowling, D. N. and Boesten, B., eds). Weinheim, Germany: VCH, pp. 67-89 (1994).
3. Hoang, T. T., Karkhoff-Schweizer, R. R., Kutchma, A. J. & Schweizer, H. P. A broad-host-range Flp-FRT recombination system for site-specific excision of chromosomally-located DNA sequences: application for isolation of unmarked *Pseudomonas aeruginosa* mutants. *Gene* **212**, 77-86 (1998).
4. Gay, P., Le Coq, D., Steinmetz, M., Berkelman, T. & Kado, C. I. Positive selection for entrapment of insertion sequence elements in gram-negative bacteria. *J. Bacteriol*. **164**, 918-921 (1985).
5. El-Sayed, A. K., Hothersall, J., Cooper, S. M., Stephens, E., Simpson, T. J. & Thomas, C. M. Characterization of the mupirocin biosynthesis gene cluster from *Pseudomonas fluorescens* NCIMB 10586, *Chem. Biol.* **10**, 419-430 (2003).
6. Mattheus, W., Gao, L. -J., Herdewijn, P., Landuyt, B., Verhaegen, J., Masschelein, J., Volckaert, G. & Lavigne, R. Isolation and purification of a new kalimantacin/batumin-related polyketide antibiotic and elucidation of its biosynthesis gene cluster. *Chem. Biol*. **17**, 149-159 (2010).
7. Deng, H., Cross, S. M., McGlinchy, R. P., Hamilton, J. T. & O'Hagan, D. In vitro reconstituted biotransformation of 4-fluorothreonine from fluoride ion: application of the fluorinase. *Chem. Biol.* **15**, 1268-1276 (2008).
8. Barnard-Britson, S., Chi, X., Nonaka, K., Spork, A. P., Tibrewal, M., Goswami, A., Pahari, P., Ducho, C., Rohr, J. & Van Lanen, S. G. Amalgamation of nucleosides and amino acids in antibiotic biosynthesis: discovery of an L-threonine:uridine-5'-aldehyde transaldolase. *J. Am. Chem. Soc.* **134**, 18514-18517 (2012).
